# Phylogenomics and sequence-structure-function relationships in the GmrSD family of Type IV restriction enzymes

**DOI:** 10.1186/s12859-015-0773-z

**Published:** 2015-10-23

**Authors:** Magdalena A. Machnicka, Katarzyna H. Kaminska, Stanislaw Dunin-Horkawicz, Janusz M. Bujnicki

**Affiliations:** 1 Laboratory of Bioinformatics and Protein Engineering, International Institute of Molecular and Cell Biology in Warsaw, ul. Ks. Trojdena 4, PL-02-109 Warsaw, Poland; 2 Institute of Molecular Biology and Biotechnology, Faculty of Biology, Adam Mickiewicz University, ul. Umultowska 89, PL-61-614 Poznan, Poland

**Keywords:** Restriction-Modification systems, Modification-Dependent systems, Type IV, Comparative genomics, Defense islands, Fold recognition, HNH endonuclease, ParB/Srx fold

## Abstract

**Background:**

GmrSD is a modification-dependent restriction endonuclease that specifically targets and cleaves glucosylated hydroxymethylcytosine (glc-HMC) modified DNA. It is encoded either as two separate single-domain GmrS and GmrD proteins or as a single protein carrying both domains. Previous studies suggested that GmrS acts as endonuclease and NTPase whereas GmrD binds DNA.

**Methods:**

In this work we applied homology detection, sequence conservation analysis, fold recognition and homology modeling methods to study sequence-structure-function relationships in the GmrSD restriction endonucleases family. We also analyzed the phylogeny and genomic context of the family members.

**Results:**

Results of our comparative genomics study show that GmrS exhibits similarity to proteins from the ParB/Srx fold which can have both NTPase and nuclease activity. In contrast to the previous studies though, we attribute the nuclease activity also to GmrD as we found it to contain the HNH endonuclease motif. We revealed residues potentially important for structure and function in both domains. Moreover, we found that GmrSD systems exist predominantly as a fused, double-domain form rather than as a heterodimer and that their homologs are often encoded in regions enriched in defense and gene mobility-related elements. Finally, phylogenetic reconstructions of GmrS and GmrD domains revealed that they coevolved and only few GmrSD systems appear to be assembled from distantly related GmrS and GmrD components.

**Conclusions:**

Our study provides insight into sequence-structure-function relationships in the yet poorly characterized family of Type IV restriction enzymes. Comparative genomics allowed to propose possible role of GmrD domain in the function of the GmrSD enzyme and possible active sites of both GmrS and GmrD domains. Presented results can guide further experimental characterization of these enzymes.

**Electronic supplementary material:**

The online version of this article (doi:10.1186/s12859-015-0773-z) contains supplementary material, which is available to authorized users.

## Background

Restriction systems are protein complexes able to recognize and destroy foreign DNA based on sequence and modification patterns. They are found in Archaea, Bacteria, Eukaryota, bacteriophages, and some viruses infecting algae [1–3]. Restriction systems can be divided into two main groups: Restriction-Modification systems (RMs) and Modification-Dependent systems (MDs), depending on their enzymatic activity and the way they recognize foreign DNA. Typically, RMs comprise two enzymatic activities targeting the same sequences in DNA: modification (e.g. methylation) and endonucleolytic cleavage, where modification protects the DNA against the cleavage. MDs exert only the cleavage activity and typically they target modified DNA sequences. Restriction enzymes are classified into four Types, with Types I, II and III encompassing all RMs and a few MDs (those that exhibit sequence specificity are grouped in Subtype IIM), and Type IV encompassing the majority of MDs [4].

RM systems genes are often found to form clusters with other factors involved in the defense against invading DNA and with mobility-related elements, such as integrases and transposases [5]. RM systems were found on plasmids, in prophages, integrons and transposons [6] while their mobility was predicted by bioinformatics analysis of genome rearrangements [7] as well as demonstrated *in vivo* [8]. A recent systematic study has proven that clustering of the defense and mobility-related genes within genomic islands in bacterial and archeal genomes (so called defense islands (DIs)) is statistically significant [9].

GmrSD is a Type IV MDs, which targets DNA that contains glucosylated hydroxymethylcytosine (HMC)—a base modification characteristic for T-even phages from the *Myoviridae* family [10]. The first GmrSD system (GmrSD CT) was discovered in *E. coli* CT596 strain and described as a heterodimer encoded by two genes: *gmrS* and *gmrD* [11]. GmrS and GmrD together act as a restriction endonuclease on DNA containing several different types of sugar-modified HMCs. GmrSD recognizes substrates with glc-HMCs containing glucose linked through an α- or β-glycosidic bond as well as gentiobiose and possibly mannose. It requires presence of calcium ions and UTP, GTP or CTP hydrolysis for cleavage. High concentrations of ATP were suggested to inhibit GmrSD activity [12].

In previous reports, GmrS domain has been shown to have a UTPase activity [12]. It was also suggested to act as an endonuclease and this activity was attributed to the presence of putative endonuclease motifs of the LAGLIDADG and HNH families. At the same time the GmrD domain was proposed to contain motifs characteristic for DNA-binding proteins [11].

In this work we present results of a bioinformatics analysis of sequence-structure-function relationships in the GmrSD protein family. We identified homologs of GmrSD proteins and analyzed their phylogenetic distribution, sequence conservation and phylogenetic relationships. Our results indicate that GmrS lacks LAGLIDADG and HNH motifs and instead belongs to the ParB/Srx fold. Moreover, its UTPase activity may be tightly associated with the nuclease activity, similarly to *Arabidopsis thaliana* sulfiredoxin and the plasmid fertility inhibition protein Osa, members of the ParB/Srx fold [13, 14]. Interestingly, we identified the HNH motif in GmrD, suggesting that both domains my confer the nuclease activity to the GmrSD complex. We also analyzed the phylogeny of GmrS and GmrD domains and the genomic context of *gmrSD, gmrS* and *gmrD* genes. Results of these analyses support the hypothesis that the GmrSD system is a part of mobile genetic elements present within genomic defense islands.

## Methods

### Sequence analyses

GmrS and GmrD domains were identified as members of PF03235 (DUF262) and PF07510 (DUF1524) PFAM [15] families using HHpred server [16], with 100 % probability and 5e^−34^ E-value for GmrS and 99.9 % probability and 5.4e^−24^ E-value for GmrD. Seed alignments of PF03235 (DUF262) and PF07510 (DUF1524) PFAM families [15] were used as queries in searches of the nonredundant sequence database clustered at 90 % sequence identity (nr90) provided with the HHsuite package [17, 18], ftp://toolkit.genzentrum.lmu.de/pub/HH-suite/databases/ (version from November 19th 2012)). For each alignment three PSI-BLAST [16] iterations with a stringent E-value threshold 10^−15^ were carried out, and the resulting position specific substitution matrices (PSSMs) were used as queries for searches with more permissive parameters (10 iterations, E-value threshold = 0.002). Sequences obtained by PSI-BLAST were then used as queries in searching the PFAM database with HHsearch in order to confirm the presence of the DUF262 and DUF1524 domains and to identify additional domains. A given match was considered true positive with E-value below 0.001 and sequence coverage above 80 % of the tentative domain length.

Preliminary multiple sequence alignments of single-domain proteins (DUF262 or DUF1524 domain only) were build using the MAFFT program with L-INS-i algorithm, while double-domain proteins were aligned with E-INS-i algorithm [19, 20]. Incomplete sequences were removed and correctness of domains assignments was checked based on the sequence conservation patterns. The final set of double-domain proteins was again aligned with MAFFT using E-INS-i algorithm. Then the GmrS domain only-containing sequences were added to one copy of this alignment with the --add option of the MAFFT program and L-INS-i algorithm and the GmrD domain only-containing sequences were added in the same way to a second copy. These two alignments were merged into one containing both two-domain and single-domain sequences. Stretches of gaps of equal length were inserted into the two-domain sequences so that the longer representatives of single-domain sequences could fit in the alignment. Finally, GmrS domain-containing and GmrD-containing sequences from each organism with only one homologue of each type in fully sequenced genome were merged into double-domain-like sequences. Any possible additional domains present in GmrSD proteins are located in the C-terminal, highly variable part of the final alignment (Additional file 1).

A collection of Python scripts using BioPython 1.6 library, BioEdit [21], Jalview [22] and UGENE 1.11.4 [23] programs were used for manipulations and visualizations of multiple sequence alignments.

### Protein fold recognition

Domain assignment, secondary structure prediction and fold-recognition (FR) analyses of selected GmrSD proteins were carried out via the GeneSilico metaserver (for references to original methods see http://genesilico.pl/meta2) [20] and HHpred server [16]. The scores reported by original methods mentioned in the manuscript have the following meaning: FFAS reports a negative Z-score. The predictions with scores lower than −9.5 contain <3 % of false positives [24] and absolute values between 14 and 7 denote moderate similarity between query and hit [25]. The HHsearch scores are probabilities for match to be a true positive (true homolog). COMA and SAM_T08 report E-values as scores. Descriptions of the scores are additionally provided in Additional file 2. Sequences of GmrS and GmrD domains of the GmrSD enzyme from *E. coli* CT596 (originally reported by Bair et al. [11]) were submitted to the metaserver as two independent queries.

### Protein structure modeling and model assessment

Homology models of the catalytic cores of GmrS and GmrD domains were constructed based on the FR results. The sequences used for modeling were from *E. coli* CT596 (originally reported by Bair et al. [11]), GenBank accessions were: 21327769 (GmrS) and 21327771 (GmrD). Target-template alignments obtained from fold recognition servers were used to prepare input modeling projects for MODELLER [26] and SWISS-MODEL [27]. The projects were prepared using Swiss-Pdb Viewer [28]. The predicted accuracy of modeled structures was calculated with the model quality assessment program MetaMQAPII [26].

For GmrS domain target-template alignment optimization was assisted by modeling of the DUF262 and DndB domains using the I-TASSER server [29]. The target-template alignment was inferred from the structure-based “consensus” alignment of preliminary GmrS models obtained using the DUF262 and DndB domain models with the 1XW4 and 1VK1 template structures. The final project was submitted to MODELLER with restraints on secondary structure based on the results from MetaServer. The resulting model was scored by the MetaMQAPII method for predicted structure quality assessment. Long insertions (residues 48–58 and 97–134) were modeled *de novo* and low-quality regions were optimized with REFINER [30] and Model/Refine Loop protocol in UCSF Chimera [31].

Molecular graphics and analyses were performed with the UCSF Chimera package [32]. 

### Protein-DNA complexes prediction

Protein-DNA docking was performed using the NPdock server [32]. The best scoring models reported by the server were chosen. For GmrS domain restraints on the protein interface were applied, based on the prediction of DNA binding residues done using the RBscore server [33] and based on the analysis of sequence conservation. The docked DNA structure is the idealized B-DNA model provided in the NPdock server example data.

The distribution of electrostatic potential was calculated using the PDB2PQR server [34] and the APBS software package [35].

### Genomic neighborhood analysis

Genomic context of GmrSD system proteins from 277 fully sequenced genomes was analyzed (available at the NCBI ftp website (ftp://ftp.ncbi.nih.gov/genomes/Bacteria/) on May 31st 2013). The GmrSD neighborhood has been defined as 10 open reading frames (ORFs) upstream and downstream of a given *gmrsd*-like gene. For 389 GmrSD homologs 7657 sequences of proteins encoded within these boundaries were obtained. These sequences were used as queries to search the PFAM database [15] with HHsearch [17, 18] with E-value threshold of 10e-3. Only top-scoring and non-overlapping matches were considered in further analyses. PFAM annotations for genomes containing at least one GmrSD homolog were retrieved from the PFAM proteomes resource (ftp://ftp.ebi.ac.uk/pub/databases/Pfam/current_release/proteomes/) and were used to calculate background frequencies of the individual domains (domain annotations at PFAM proteomes were available for 231 genomes out of the 277 analyzed). Statistical significance of the enrichment of PFAM domains in GmrSD genomic neighborhood was evaluated using the test of equal or given proportions implemented in the R package. Confidence intervals for the difference of probabilities of finding each domain in GmrSD genomic neighborhood and in domains set obtained from PFAM proteomes were calculated. A Bonferroni correction was used to control the familywise error rate: individual confidence levels of the confidence intervals returned for each PFAM domain were set to $1-\frac{0.05}{number\  of\  PFAM\  domains}$, since the number of tested hypothesis was equal to the number of different PFAM domains. All domains were ranked based on ascending value of the lower boundary of this confidence interval. A domain was recognized as “enriched” in the GmrSD genomic neighborhood if the confidence interval was above zero. 

### Phylogenetic analysis

A set of representative sequences used for phylogenetic analysis was selected from a multiple sequence alignment of the GmrSD family based on a preliminary Neighbour Joining tree calculated in Jalview using BLOSUM62 substitution matrix. The preliminary tree was evenly sampled in order to collect the representative group of sequences and – if possible – the sequences from model organisms were selected. Manually chosen conserved regions of the multiple sequence alignment were used for tree calculations. To examine coevolution of the GmrS and GmrD domains the alignment was divided into parts representing each domain (121 GmrS domain sequences and 134 GmrD domain sequences). FastTree [36, 37] with WAG substitution model and default options was used to calculate three approximately-maximum-likelihood phylogenetic trees: for GmrS domains sequences, GmrD domains sequences, and for sequences which contain both domains (both encoded as double-domain and from merged pairs of single-domain proteins). The reliability of each split in the trees was estimated by computing local support values with the Shimodaira-Hasegawa test. Splits with SH-like support values below 0.8 were collapsed into polytomies and leaves that corresponded to either GmrS or GmrD proteins, for which we could not assign the other counterpart (GmrD or GmrS, respectively), were removed. The resulting GmrS and GmrD domains trees were visualized as a tanglegram using Dendroscope 3 [38]. The double-domain proteins tree was visualized using Archaeopteryx [39]. Alignments used for tree calculations are available as Additional files 3, 4 and 5).

Analysis of positive and negative selection was performed for three subgroups of GmrSD sequences, chosen based on the phylogenetic tree of double-domain GmrSD proteins and merged GmrS-GmrD protein pairs (Additional file 6). The main criterion for the choice of sequences was low divergence (we used a criterion of all branch lengths being smaller than one). We also wanted one group to contain the GmrS and GmrD sequences from *E. coli* CT596. Coding DNA alignments were build based on protein alignments using the Pal2Nal server [40]. Finally, the detection of sites under positive or negative selection was performed using the SLAC, FEL, REL [41] and MEME [42] methods provided by the Datamonkey server [43].

A tree of life used to present the phylogenetic distribution of GmrSD homologs was created using the tree generator provided by the iTOL application [44].

## Results and discussion

### GmrSD family comprises mainly fusion proteins with conserved GmrS-like and GmrD-like domains

**Fig. 1:**
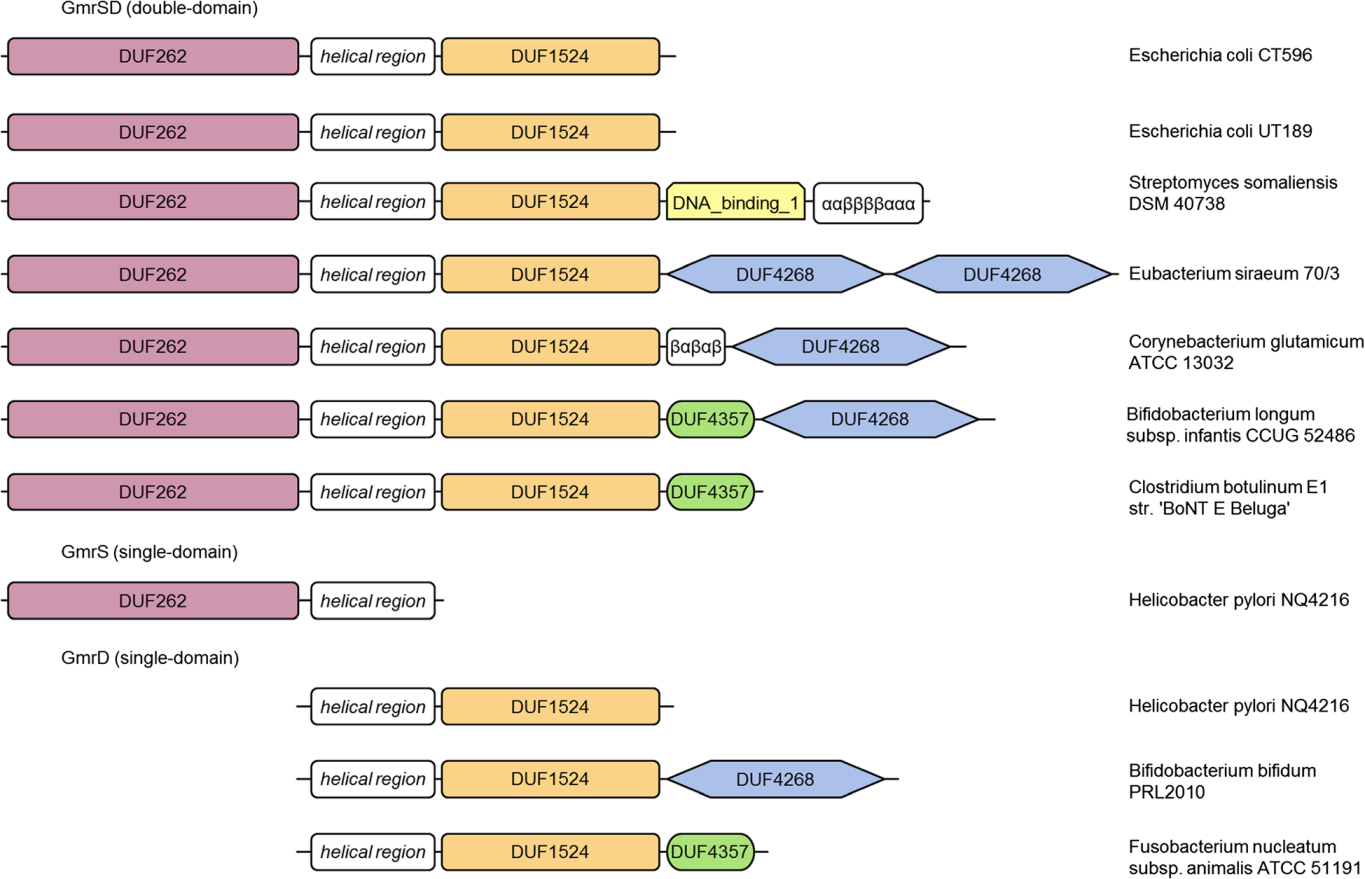
Domain architectures of the GmrSD family members. Predicted domains are shown using different colors and shapes. Regions with no domain assignment, which may or may not form independent domains, are indicated as white boxes with the corresponding patterns of predicted secondary structure (α-helices and β-strands)

We initiated the sequence analysis of the GmrSD system by identifying GmrS and GmrD proteins as members of PF03235 (DUF262) and PF07510 (DUF1524) PFAM [15] families, respectively. We collected GmrSD family members using PSI-BLAST [45] and verified the presence of DUF262 and DUF1524 domains using HHsearch (see Methods for details). We found that some of GmrSD homologs appear to have not only DUF262 and/or DUF1524 but also one or two additional domains (see Fig. 1).

Among additional domains found in GmrSD homologs we identified: DUF4268 (PF14088), DUF4357 (PF14267), and DNA_binding_1 domain (PF01035). DUF4268 (PF14088) and DUF4357 (PF14267) are domains of unknown function found in Bacteria and Archaea. DUF4268 is most often associated with DUF262 (GmrS) and DUF1524 (GmrD), while DUF4357 was also found in proteins containing the GIY-YIG nuclease superfamily domain [46] and Bacteriophage T5 Orf172 DNA-binding domain. DNA_binding_1 (PF01035) is a DNA binding domain from DNA repair proteins, which remove methyl groups from 6-*O*-methylguanine to one of their own cysteine residues [47]. We found PF01035 in several GmrSD protein homologs, mainly from various *Streptomyces* species.

**Fig. 2:**
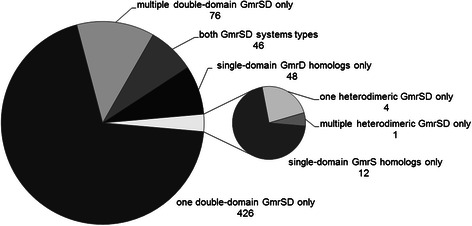
GmrSD protein sets present in fully sequenced genomes

Altogether, we gathered 1119 GmrSD homologs (both single- and double-domain proteins) from 769 organisms, 613 of which have fully sequenced genomes (according to the GOLD database v.4 [48]). Among these 613 organisms 15 belong to Archaea, 595 to Bacteria and three are eukaryotic. 862 sequences represent the double-domain form of the GmrSD system. A number of genomes encode more than one complete GmrSD system and/or its incomplete variants (only GmrS or only GmrD protein) (Fig. 2). Our results show that a fused, double-domain form of GmrSD system is much more abundant than the one comprising separate, single-domain GmrS and GmrD proteins. The requirement for the presence of both subunits for the full activity of the enzyme could have promoted the evolution of fusion proteins. In cases in which separate GmrS and GmrD homologs are located very close to each other in the genome, annotation/sequencing errors may have occurred and in reality such entries may represent a double-domain GmrSD rather than heterodimeric GmrSD system [as it turned out for the founding member of the GmrSD family in *E. coli* CT596 (L. W. Black, personal communication)].

**Fig. 3:**
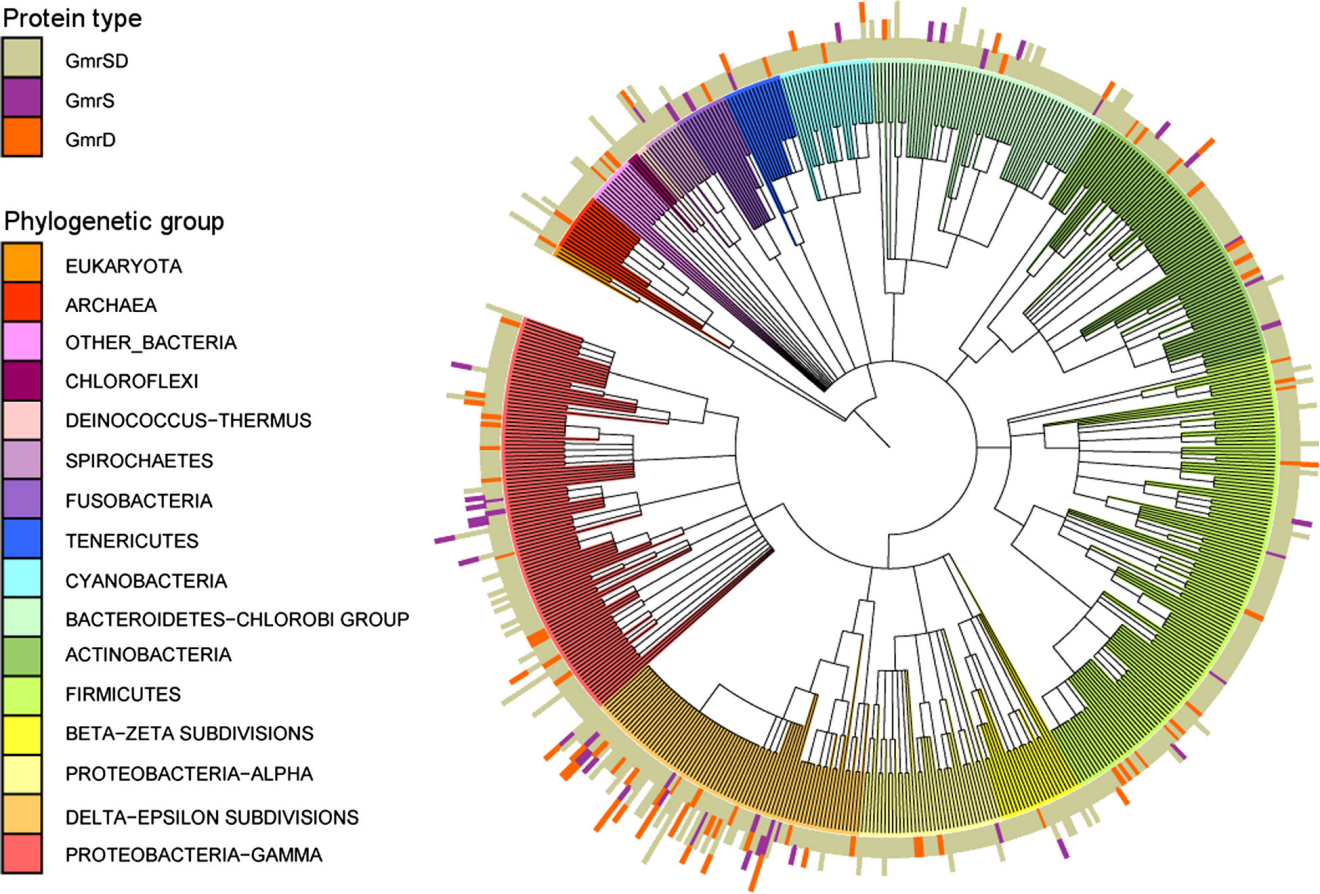
Phylogenetic distribution of GrmSD homologs. Detected presence of at least one of the GmrS, GmrD and/or double-domain proteins homologs was marked on a tree of life generated for organisms with fully sequenced genomes. The tree was created using the iTOL application [44, 73]. The histogram outside the tree illustrates the number of GmrS (in purple), GmrD (in orange) and double-domain (in olive) homologs encoded by each genome. Species were assigned to phylogenetic groups and clades were colored accordingly (see the legend). For clarity, species names are not shown

We also checked the distribution of *gmrSD* system genes in different phyla. Representatives of this family were found across most of the major bacterial lineages as well as in several Archaea and Eukarya (representatives of Bacillariophyta and Chlorophyta phyla and Heterolobosea class) (Fig. 3). The highest number and variety of these proteins per genome can be found in Epsilonproteobacteria, mainly in *Helicobacter* species, which carry up to five GmrSD homologs. This phylogenetic group is also the one with the highest number of heterodimeric GmrSD systems, which in general are rarely present in other groups. At the same time, double-domain GmrSD systems are distributed widely in various prokaryotic phyla.

### Sequence conservation in the GmrSD protein family

**Fig. 4:**
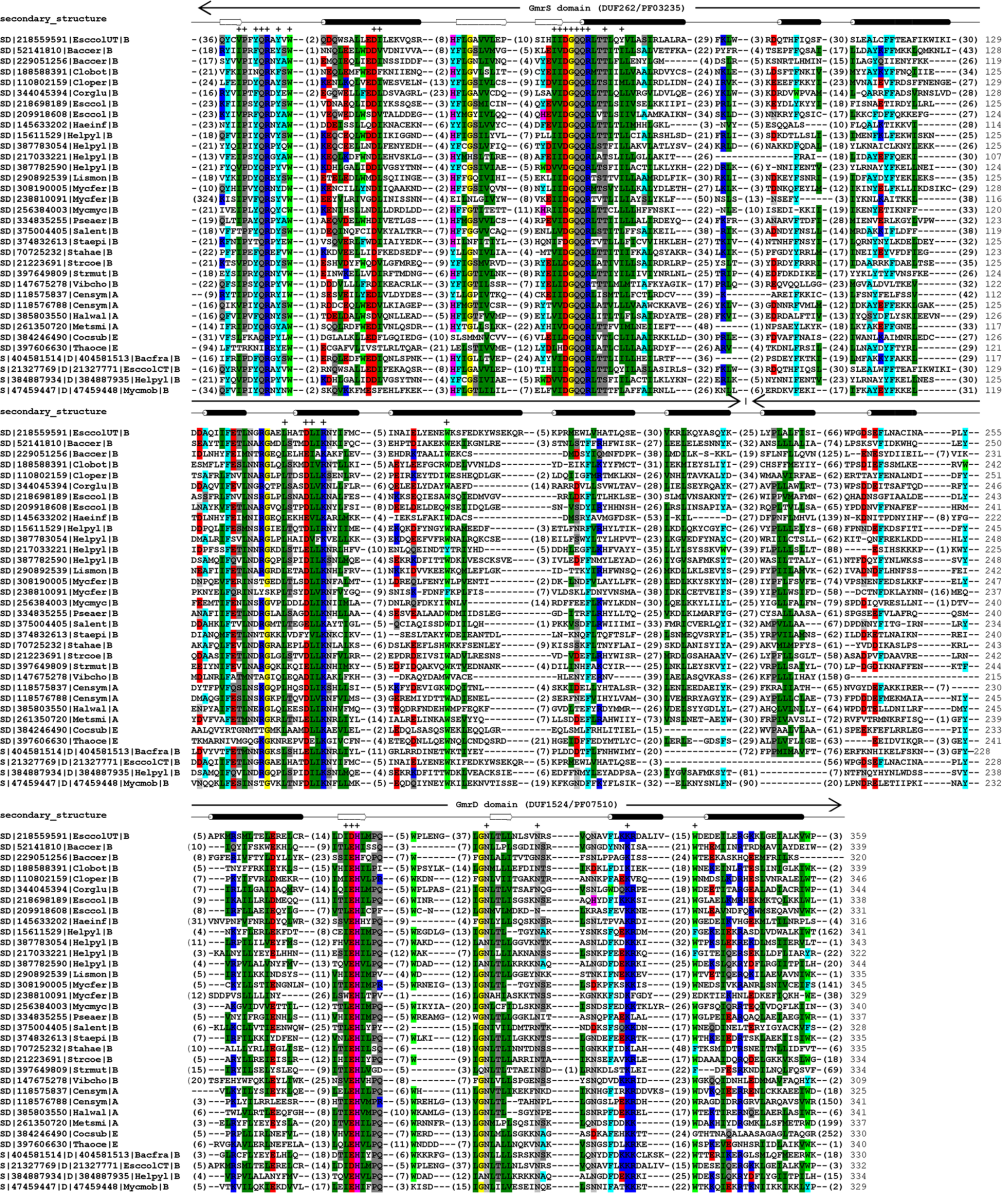
Multiple sequence alignment of representative members of the GmrSD family. The alignment contains double-domain protein sequences (indicated as GmrSD), GmrS-domain only (GmrS) and GmrD-domain only (GmrD). Domain ranges are indicated above the alignment. Genus and species name abbreviations used: Baccer–*Bacillus cereus*, Bacfra–*Bacteroides fragilis*, Censym–*Cenarchaeum symbiosum*, Chlvar–*Chlorella variabilis*, Clobot–*Clostridium botulinum*, Cloper–*Clostridium perfringens*, Cocsub–*Coccomyxa subellipsoidea*, Corglu–*Corynebacterium glutamicum*, Esccol–*Escherichia coli*, Haeinf–*Haemophilus influenzae*, Halwal–*Haloquadratum walsbyi*, Helpyl–*Helicobacter pylori*, Lismon–*Listeria monocytogenes*, Metsmi–*Methanobrevibacter smithii*, Myccap–*Mycoplasma capricolum*, Mycfer–*Mycoplasma fermentans*, Mycmob–*Mycoplasma mobile*, Mycmyc–*Mycoplasma mycoides*, Pseaer–*Pseudomonas aeruginosa*, Salent–*Salmonella enterica*, Staepi–*Staphylococcus epidermidis*, Stahae–*Staphylococcus haemolyticus*, Strcoe–*Streptomyces coelicolor*, Strmut–*Streptococcus mutans*, Strpne–*Streptococcus pneumoniae*, Thaoce–*Thalassiosira oceanica*, Vibcho–*Vibrio cholerae*. Kingdom information: (A) – Archaea, (B) – Bacteria, (E) – Eukaryota. UT, CT – GmrSD UT and GmrSD CT enzymes [11, 74]. Residues conserved in > 95 % sequences are marked by “+”. The color-shading in this figure represents sequence conservation in the selected subset of proteins only. The complete alignment of 1094 sequences is available as Additional file 1. Consensus secondary structure is indicated above the alignment as tubes (helices) and arrows (strands) for the *E. coli* GmrSD UT protein

We constructed a multiple sequence alignment that contains sequenecs of both single- and double-domain GmrSD family members. The alignment contained 862 sequences of double-domain GmrSD proteins, 25 predicted pairs of single-domain GmrS and GmrD proteins and 207 single-domain GmrS and GmrD homologs not organized into pairs (Additional file 1). Figure 4 presents an alignment of GmrSD sequences from selected model organisms.

The analysis of conservation patterns reveals two regions of sequence conservation in the GmrS domain: one corresponding to residues 8–98 in GmrS from *E. coli* CT596 and another one corresponding to residues 181–278. The first region possesses a highly conserved motif (I/V)(I/V)DGQQRLTT(I/L/V)xLL, in which the underlined DGQQR sub-motif is conserved in nearly 100 % of sequences. It resembles the DGQHR signature motif of the so-called DGQHR domains (TIGR03187/PF14072). However, none of the identified conserved regions contains previously suggested LAGLIDADG and HNH endonuclease motifs [11].

The analysis of the GmrD-domain part of the alignment revealed a region of highly conserved residues from position 112 to 190 in GmrD from *E. coli* CT596, which can be divided into three motifs: (I **/** L **/** V)(E **/** D)H(I/L/V)xPQ, L(G/A)NLxLLxxxN, NxxFxxKK (highly conserved residues are underlined). A fourth, weakly conseverd motif Wxxxx(I/L/V)xxRxxxLxxxxxx(I/L/V)W is located between residues 211 and 231.

### GmrS exhibits ParB/Srx fold while GmrD is a putative HNH endonuclease

We carried out domain assignment for GmrS, GmrD and double-domain proteins using HMMPFAM and HHSEARCH_CDD tools via the GeneSilico metaserver [49]. According to the best scored matches to PFAM [15] and CDD [50] databases, GmrS proteins are members of DUF262, while GmrD proteins correspond to DUF1524. Both GmrS and GmrD queries reported matches to COG1479, which covers fusion GmrSD proteins. All double-domain proteins possess DUF262 in their N-terminal part and DUF1524 in C-terminal part, frequently connected by an α-helical linker of 200–400 residues.

DUF262 (PF03235) is a member of the ParB-like superfamily (ParBc) that includes nucleases related to ParB as well as uncharacterized proteins. ParB is a component of the par system, which mediates accurate DNA partition during cell division. This bacterial protein was found to be homologous to a functionally unrelated eukaryotic enzyme sulfiredoxin (Srx) [51, 52]. Moreover, we found that DUF262 domain exhibits in its N-terminal part significant similarity (E-value < 0.001 for HMMPFAM or probability > 60 % for HHSEARCH_CDD) to the DGQHR domain (TIGR03187/PF14072). DGQHR domain is a member of the DndB superfamily, which comprises DNA-sulfur modification-associated bacterial proteins that are likely to be necessary for binding to DNA and recognizing the modification sites [53]. The DGQHR domain has several conserved residues, including a QR pair and an FxxxN motif, but its most characteristic feature is a nearly invariant pentapeptide motif DGQHR. These three motifs can be all identified in the alignment of the GmrS domain (Fig. 4).

The GmrS domain was predicted to be structurally similar to the ParB/Sulfiredoxin fold (d.268.1 in SCOP [54]). Fold recognition methods included in the GeneSilico metaserver (FFAS, HHSEARCH, COMA, and SAM_T08) returned this prediction with scores from -7 to -9.6, from 50 to 86, from 0.007 to 8.5e-05 and from 0.39 to 1.7, respectively. Among the best scored matches, several sulfiredoxins and a ParB-like nuclease structures can be found (represented by [PDB:1XW3, PDB:1YZS, PDB:3HY2, PDB:2RII] and [PDB:1VK1]). The scores of individual methods cannot be directly compared (see the Methods section for details) but they suggest that the prediction is of moderate (for FFAS, HHSEARCH and COMA) or low (SAM_T08) confidence. However, having the results from multiple methods provided by the metaserver we could draw consensus conclusions regarding the predicted fold of the GmrS domain.

**Fig. 5:**
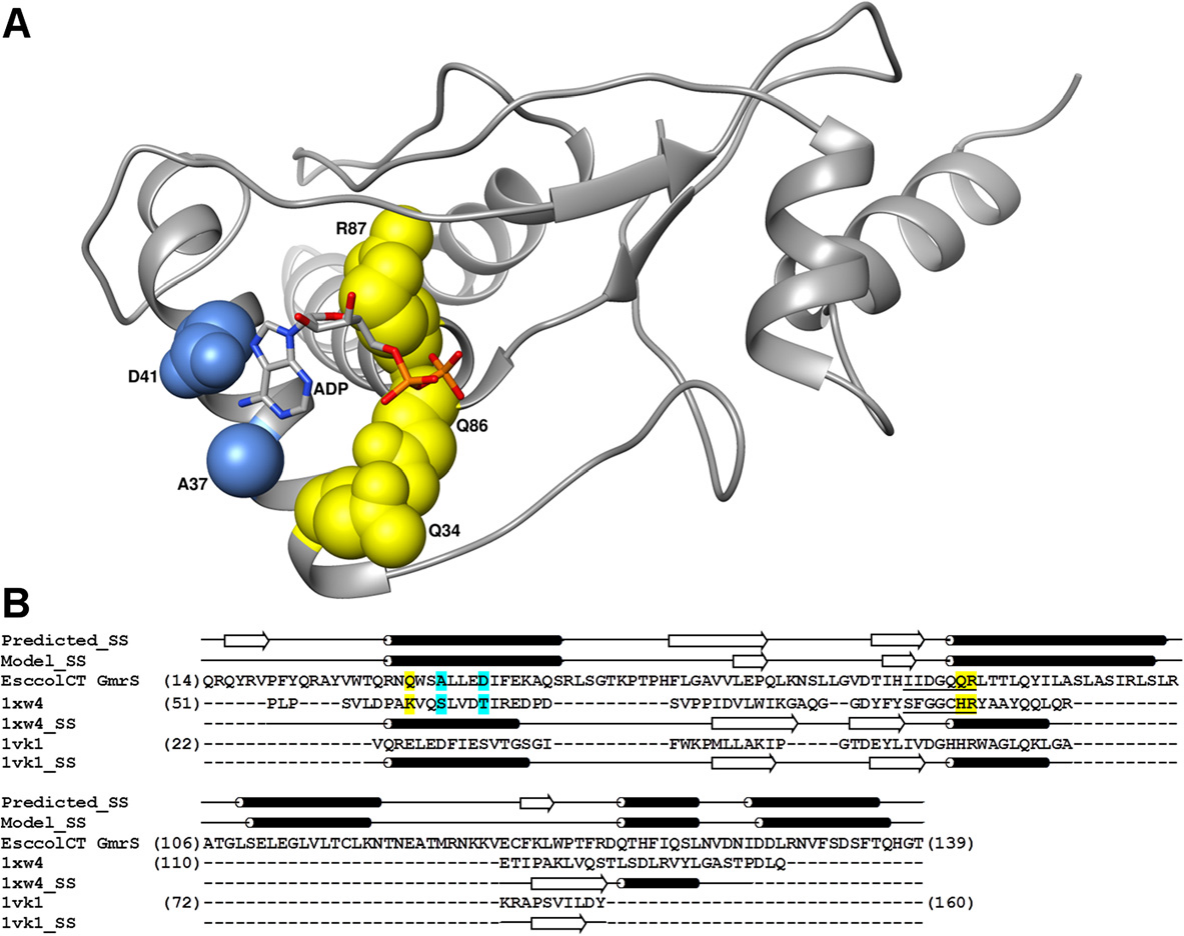
Structural model for the catalytic core of the GmrS domain. **a** The protein backbone is shown in the ribbon representation. Residues predicted to interact with the phosphate groups of the ligand and with the base are shown in the space-filled representation and colored yellow and blue, respectively. The ADP molecule is colored according to the atom type. The coordinates are available from ftp://genesilico.pl/iamb/models/GmrSD/GmrS.pdb. **b** Target-template alignment used for comparative modeling of the GmrS domain from the GmrSD CT enzyme. Consensus secondary structure calculated by the GeneSilico metaserver (“Consensus_SS”) and secondary structure of the GmrS model (“Model_SS”) are indicated above the alignment as tubes (helices) and arrows (strands). Secondary structures of the two templates used for modeling (human sulfiredoxin [PDB:1XW4], and ParB-like nuclease from *Pyrococcus furiosus* [PDB:1VK1]) are indicated below the template sequences. Residues which participate in ATP binding in human sulfiredoxin [56] and their equivalents in the GmrS domain are indicated in blue (adenine binding) and yellow (phosphates binding). Sulfiredoxin signature motif and the most conserved motif of the GmrS domain are underlined

We modeled the conserved core of the GmrS domain using two templates: the crystal structure of the human sulfiredoxin in complex with ADP [PDB:1XW4] and the crystal structure of a ParB-like nuclease from *Pyrococcus furiosus* [PDB:1VK1] (see Methods for details). The modeled GmrS catalytic core is an α/β structure containing five α-helices and two antiparallel β-strands and it comprises residues 15–174 of the GmrSD CT protein (Fig. 5). MetaMQAPII [55] model assessment method predicted that the model exhibits a root mean square deviation (RMSD) from the true structure on the order of 3.4 Å, and a predicted GDT_TS score of 47.7, which indicates a reasonably accurate structure. At the same time MetaMQAPII reported GDT_TS scores of 65.9 and 86.6, and RMSD 3.6 Å and 1.2 Å for the templates used for modeling (1XW4 and VK1, respectively), even though these are experimentally determined structures for which real values are 0 Å RMSD and 100 GDT_TS. The 1XW4 structure represents only a part of the molecule and may be stabilized by the remaining part while 1VK1 may be stabilized by metal ions, which is not taken into account by the model scoring method.

The analysis of the model and sequence conservation of the GmrS domain revealed a putative NTP binding site. We found that in the GmrS/Srx sequence alignment the strongly conserved (I/V)-D-G-Q-Q-R motif of GmrS proteins was consistently aligned to the Srx signature sequence F-(G/S)-G-C-H-R (Fig. 5b) [52]. As it was revealed in a series of crystal structures of the Srx-ATP [PDB:3HY2, PDB:3CYI] and Srx-ADP [PDB:1XW4] complexes, this signature sequence is a part of a nucleotide-binding pocket of Srx proteins [56–58]. The H100 and R101 residues of Srx are the main constituent of the pocket and make hydrogen bonding interactions with β- and γ-phosphate groups. The adenosine ring is bound by S64 and T68, while K61 is responsible for hydrogen-bonding with the α-phosphate (note that these positions are not a part of the signature sequence). The Srx ATP binding motif was reported to resemble motifs found in other proteins such as tyrosine phosphatases and DNA ligases, in which the H100 of Srx is replaced by several main chain amide groups [27, 59, 60]. We propose that the highly conserved (I/V)-D-G-Q-Q-R motif of GmrS proteins is an equivalent of the Srx signature sequence (Fig. 5b) and may participate in NTP binding (Fig. 5a). According to the target-template alignment, A37 and D41 in GmrS correspond to the adenine binding residues in Srx: S64 and T68. However, since the preferred substrate for GmrS is UTP and not ATP, the interaction with the base may differ between these two enzymes.

ParB enzymes such as the one from *E. coli* and ParB-like nuclease from *P. furiosus* were long known to possess a nuclease activity [61, 62]. Quite recently one of the sulfiredoxins (namely the one from *Arabidopsis thaliana*) was reported to have a dual redox-dependent sulfinic acid reductase and redox-independent nuclease function [13]. Afterwards the plasmid fertility inhibition protein Osa was found to exhibit the ATPase and DNase activities and to have a domain of the ParB/Srx fold [14]. The active site of the Osa protein corresponds to the ATPase active site of sulfiredoxins and was shown to be responsible for both Osa activities. Furthermore, the ATP concentration regulates Osa nuclease activity. In high ATP concentrations the nuclease activity is inhibited, possibly due to the presence of a phosphate moiety, retained in the active site after ATP hydrolysis.

The Srx signature motif contains also the C99 residue, which is necessary for the reduction of the cysteine sulfinic acid moiety within the active site of the peroxiredoxins [56]. This catalytically important cysteine residue is in GmrS replaced by Q85. Since GmrS is not predicted to participate in thiosulfinate formation, this residue most probably plays a different role. Moreover, the substitution of this catalytic Cys by Ser in sulfiredoxin from *A. thaliana* did not affect its nuclease activity, while in the Osa protein this position is occupied by Met.

Taking these data into account, we conclude that GmrS may also have two activities: NTPase and nuclease. This notion is supported by the fact that Osa is inhibited by high ATP concentrations, which was also suggested for GmrSD [11]. Unfortunately, the influence of UTP, CTP or GTP on Osa activity is unknown, but we suggest that these nucleotides may not block the nuclease activity.

DUF1524, reported as the best match for the GmrD protein by the domain recognition methods, corresponds to the PF07510 family. It is a member of His-Me finger endonuclease superfamily, which contains a diverse range of endonucleases families, including the HNH motif family. We found that GmrD homologs also show similarities to CRISPR-associated proteins (cas) represented by TIGR01865 family (cas_Csn1) and COG3513 (predicted CRISPR-associated nuclease, containing McrA/HNH-nuclease and RuvC-like nuclease domain).

The 3D structure of the GmrD domain was predicted to be similar to HNH endonucleases from the His-Me finger superfamily. Best matches (mainly from FFAS and HHSEARCH) referred to [PDB:4H9D, PDB:4OGE, PDB:4OO8, PDB:1OUP, PDB:2PU3] and [PDB:2VND] structures and were reported by FFAS with scores ranging from -8.5 to -13.7 and HHSEARCH with probabilities ranging from 64.2 to 85.9 %. [PDB:4H9D] is a HNH endonuclease from deltaproteobacterium *Geobacter metallireducens* assigned to PF01844 PFAM family, a member of His-Me finger endonuclease superfamily. [PDB:4OGE] and [PDB:4OO8] are structures of the HNH endonuclease domain from the Cas9 protein of CRISPR-Cas system. [PDB:1OUP, PDB:2PU3] and [PDB:2VND] are classified as members of Endonuclease I family, also within the His-Me finger endonucleases superfamily (SCOP d.4.1.6). These structures were identified as best possible templates for a part of the target sequence corresponding to residues 75–226 of the GmrD domain from *E. coli* CT596 GmrSD protein. The lengths of aligned fragments were however not always the same and depended mainly on the fold recognition server. Secondary structure prediction for this region revealed an α-β-α-α-β-α pattern.

**Fig. 6:**
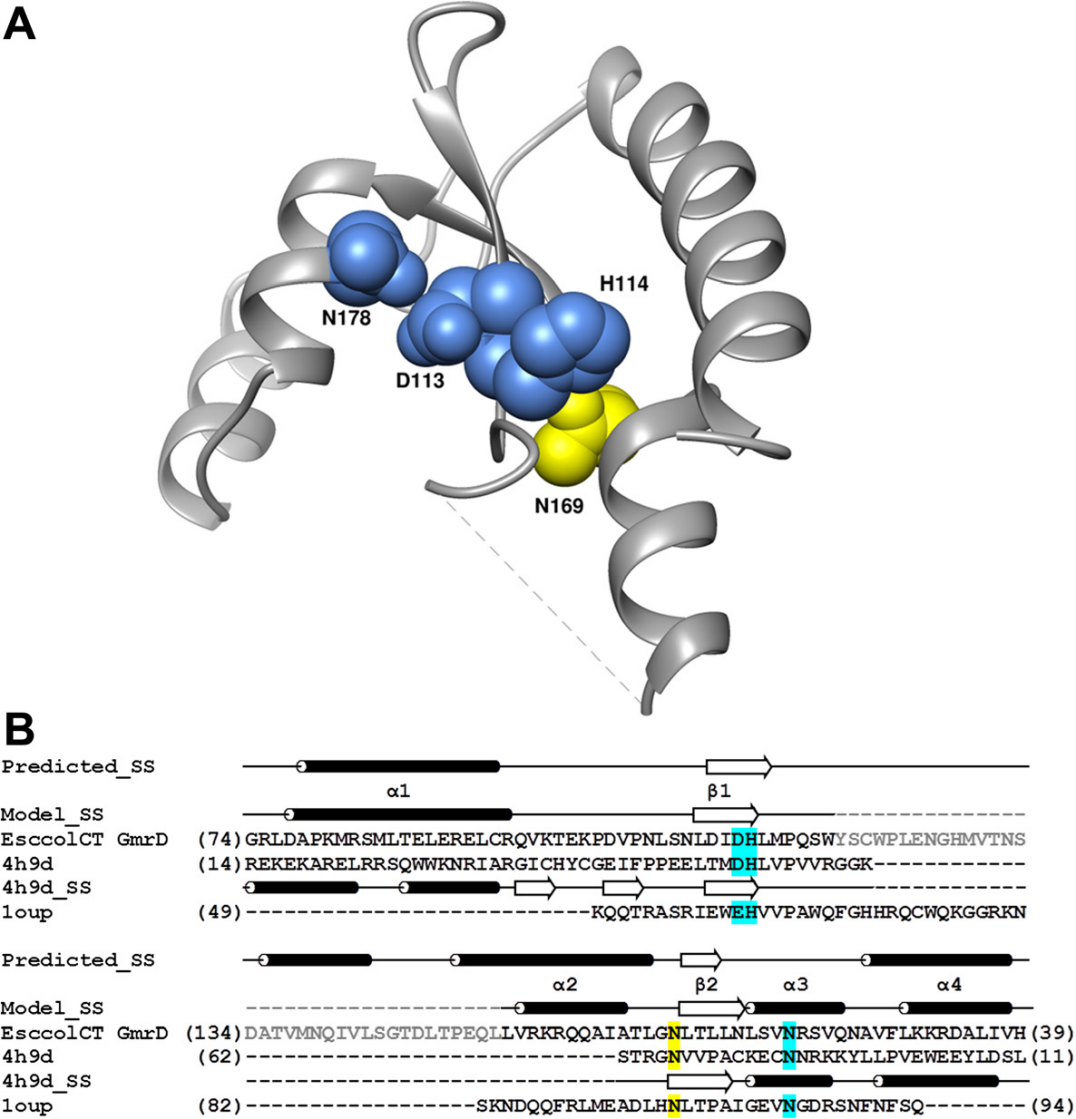
Structural models for the catalytic core of the GmrD domain. **a** The protein backbone is shown in the ribbon representation. Predicted functionally important residues are labeled and shown in the space-filled representation. Residues predicted to be involved in catalysis are colored in blue, N169 predicted to fulfill a crucial structural role – in yellow. The dashed line indicates the location of the long insertion removed from the model. The coordinates are available from ftp://genesilico.pl/iamb/models/GmrSD/GmrD.pdb. **b** Target-template alignment used for comparative modeling of the GmrD domain from the GmrSD CT enzyme. The sequence of Vvn endonuclease [PDB:1OUP] is included in the alignment to indicate the active site residues, although this structure was not used as a template for comparative modeling. Consensus secondary structure calculated by the GeneSilico metaserver (“Predicted_SS”) and secondary structure of the GmrD model (“Model_SS”) are indicated above the alignment as tubes (helices) and arrows (strands). Numbering of the secondary structure elements refers to the model. Secondary structure of the template used for modeling (Mn-dependent Gme HNH nicking endonuclease from *Geobacter metallireducens* GS-15, [PDB:4H9D]) is indicated below the template sequence. Residues predicted to be involved in catalysis are marked in blue, the Asn residue predicted to fulfill a crucial structural role – in yellow. The sequence of the long insertion not included in the model is marked in grey letters

We modeled the GmrD HNH catalytic core (residues 75–196) using the [PDB:4H9D] structure as a template (Fig. 6). For the remaining part of the sequence no modeling templates with high sequence similarity to the query were identified by any fold recognition method used. The long insertion (Y121 – L156) was removed from the model, also due to the lack of an appropriate modeling template. The GmrD catalytic core contains two β-strands and four α-helices. The β-strands form an anti-parallel hairpin. MetaMQAPII predicted the model to have a RMSD from the true structure of about 7.25 Å and a GDT_TS score of 16.95. These relatively low scores may in part result from the specific features of the template structure, for which MetaMQAPII predicts 4.47 Å RMSD and 40.1 GDT_TS (even though it is an experimentally determined structure and real values are 0 Å RMSD and 100 GDT_TS). This structure represents also only a part of the molecule and may be stabilized by either the remaining part and/or metal ions, which is not taken into account by the model scoring method.

Based on the structural and biochemical data available for other HNH endonucleases, residues D113, H114, N169 and N178 were predicted to be crucial for GmrD structure and function. Biochemical data regarding the HNH endonuclease from *G. metallireducens*, whose structure [PDB:4H9D] was used as template, are limited: from the mutagenesis results, it was concluded that H54, N68, and N77 are critical for both enzyme activity and for maintaining proper structural folding, while D53 residue is less important for structure integrity and may contribute to the catalytic site by stabilizing a metal ion [63]. The equivalents of these residues in the GmrD domain are: H114, N169, N178 and D113, respectively (Fig. 6b). Additionally, we compared the structure and sequence of the active site of the periplasmic endonuclease Vvn in complex with the DNA product [PDB:1OUP_A] to the model of the GmrD catalytic core (see the structure based sequence alignment in Fig. 6b). The D113 and N178 residues of GmrD correspond to Vvn E79 and N127, respectively, which coordinate Mg^2+^ [64], hence they may be responsible for the Ca^2+^ ion binding. H80 in Vvn (substituted with A in the [PDB:1OUP] structure) acts as the general base what suggests that H114 in GmrD may play the same role. N169, which is homologous to N68 from [PDB:4H9D], is then most probably involved in maintaining the proper structure of the protein. Overall our model supports the prediction that the GmrD domain possesses an HNH endonuclease active site but it is not sufficient to predict how it interacts with the substrate.

Based on results of our bioinformatics analyses we propose that the HNH motif of the GmrD domain is responsible for the endonucleolytic cleavage of the DNA substrate, while the conserved (I/V)-D-G-Q-Q-R motif of the GmrS domain is responsible for the UTPase activity. It is, however, possible that GmrS has an additional nuclease activity, like some members of the ParB/Srx fold [13, 14, 62]. Our data do not allow us to propose an exact model of GmrSD-DNA interactions and to identify the region of the enzyme that is responsible for the recognition of the glc-HMC containing substrates. We performed protein-DNA docking and electrostatic potential analysis for the GmrS and GmrD domains models (see results in Additional file 7). However, since the models of GmrS and GmrD catalytic cores comprise only parts of the proteins, and we do not have information about the remaining parts, both the electrostatic potential calculation and docking analyses are very speculative. Interestingly, the substrate recognition domain of the PvuRts1I – another Type IV restriction enzyme that cleaves DNA containing glc-HMC does not exhibit sequence or structural similarity to any region of the GmrSD enzyme. In PvuRts1I the substrate recognition and DNA cleavage functions are separated in two protein domains: the N-terminal, PD-(D/E)XK catalytic domain and the C-terminal SRA domain, responsible for substrate recognition [65, 66]. This division of labor between domains supports the notion that the specificity for glc-HMC-containing DNA emerged more than once in the course of evolution and is associated with endonucleases exhibiting different folds.

### Molecular evolutionary analyses and genomic context of GmrSD suggest it being a part of mobile genetic elements

In order to analyze the conservation of GmrSD system genomic neighborhood we compared domain composition of proteins encoded by up to 10 ORFs upstream and downstream from each GmrSD homolog. To assess characteristic features of the domain composition of these proteins, we calculated occurrences of each domain type both in the GmrSD genomic neighborhood and in all complete proteomes of GmrSD system-carrying species, obtained from PFAM. Based on this data we ranked all domains according to the difference in the probability of finding these domains in the neighborhood and in the proteomes. We identified 169 PFAM domains, which are more likely to be found in the GmrSD genomic neighborhood than in any random place in the genome. Twelve most significantly enriched domains are listed in Table 1. A complete list of domains enriched in the GmrSD genomic neighborhood is available as Additional file 8.

**Table 1:** PFAM domains most frequently present in the genomic neighborhood of GmrSD proteins

Domains	Description
Methylase_S, N6_Mtase, HsdM_N, HSDR_N	Elements of Type I RM systems
Helicase_C	Present in a wide variety of helicases and helicase-related proteins. In the GmrSD genomic neighborhood this domain most frequently cooccurs with domains of restriction endonucleases: ResIII and HSDR_N, and with DUF3387. A domain similar to DUF3387, the putative inner membrane protein DUF1819, was found among overrepresented gene families in the genomic neighborhoods of the Pgl defense system [9].
ResIII	Restriction endonuclease domain from the Type III RM systems
Tetratricopeptide repeat (TPR_15)	A structural motif responsible for protein-protein interactions identified in a wide variety of proteins [75].
DUF262	The domain which we identified to be present in the GmrS protein.
SMC_N	Found in the N terminus of structural maintenance of chromosomes (SMC) proteins and in RecF and RecN proteins, which are involved in the DNA metabolism and recombination. Proteins from the GmrSD genomic neighborhood that contain this domain most often have only this domain, which suggests that they are homologs of the DNA replication and repair protein RecF, or they also possess the AAA_23 domain, which is characteristic for the DNA repair protein RecN.
Phage_int_SAM_4 (Phage integrase, N-terminal SAM-like domain)	Found in site-specific tyrosine recombinases characteristic for integrons – genetic elements able to acquire and rearrange open reading frames (ORFs) embedded in gene cassette units and convert them to functional genes by ensuring their correct expression [76].
HTH_19	DNA binding domain. In proteins from the GmrSD neighborhood it is most often a part of transcription regulators.

In general, GmrSD homologs tend to colocalize with other RM systems elements and their neighborhood resembles the defense islands described by Makarova et al. [9, 67]. Genomic neighborhood of the GmrSD proteins is also enriched in genes involved in the DNA metabolism (replication and repair) and phage-related elements. This latter finding corresponds well with the fact that two ORFs identified in the nearest neighborhood of the *gmrS* and *gmrD* genes in the *E. coli* CT596 genome are similar to phage tail fiber assembly genes and a phage invertase [11]. It indicates that this region of the *E. coli* CT596 genome might be a mobile genetic element such as a cryptic prophage.

Among other defense elements in the GmrSD neighborhood Type I RM systems [represented by Methylase_S (PF01420), N6_Mtase (PF02384), HsdM_N (PF12161) and HSDR_N (PF04313) domains] are present most frequently. We also noticed that some of GmrSD homologs found by our sequence searches are annotated as “RloF” or “RloF-like”. Indeed, DUF262 (GmrS domain) has been previously identified within the Type I RM system locus in *Campylobacter jejuni* by Miller et al. [68], who have assigned the name RloF (R-linked ORF F) to the common elements located between genes encoding the specificity subunits (HsdS) and restriction endonuclease subunits (HsdR) of the Type I RM systems.

Type I RMs elements, integrases, helicases and transcription regulators, which we found to be among most important contributors to the GmrSD genomic neighborhood, were reported among protein families overrepresented in the genomic neighborhoods of DUF262 [9, 67], which we identified to be the GmrS domain. Makarova et al. also showed that DUF262 is often associated with the PglZ protein from the Phage Growth Limitation system [69, 70]. However, we did not observe a reciprocal relationship between GmrS and PglZ domains in our studies of the GmrSD genomic context, which may be due to differences in the applied methods.

Finally, we found tetratricopeptide repeat (TPR_15) domains and DUF262 to be enriched in the GmrSD neighborhood. Proteins containing the TPR_15 domain were found to be overrepresented in the genomic neighborhoods of the COG1479 family proteins in defense islands [9]. Indeed, our analysis of domain composition of GmrSD homologs showed that they are COG1479 members, hence our results are in agreement with these published data. The fact that the DUF262 domain can be so frequently found close to GmrSD homologs suggests that GmrS domain-containing proteins tend to be genomic neighbors of other proteins from the family of GmrSD homologs, while GmrD domain-containing proteins localize in separate regions of genomes.

### Coevolution of GmrS and GmrD domains

**Fig. 7:**
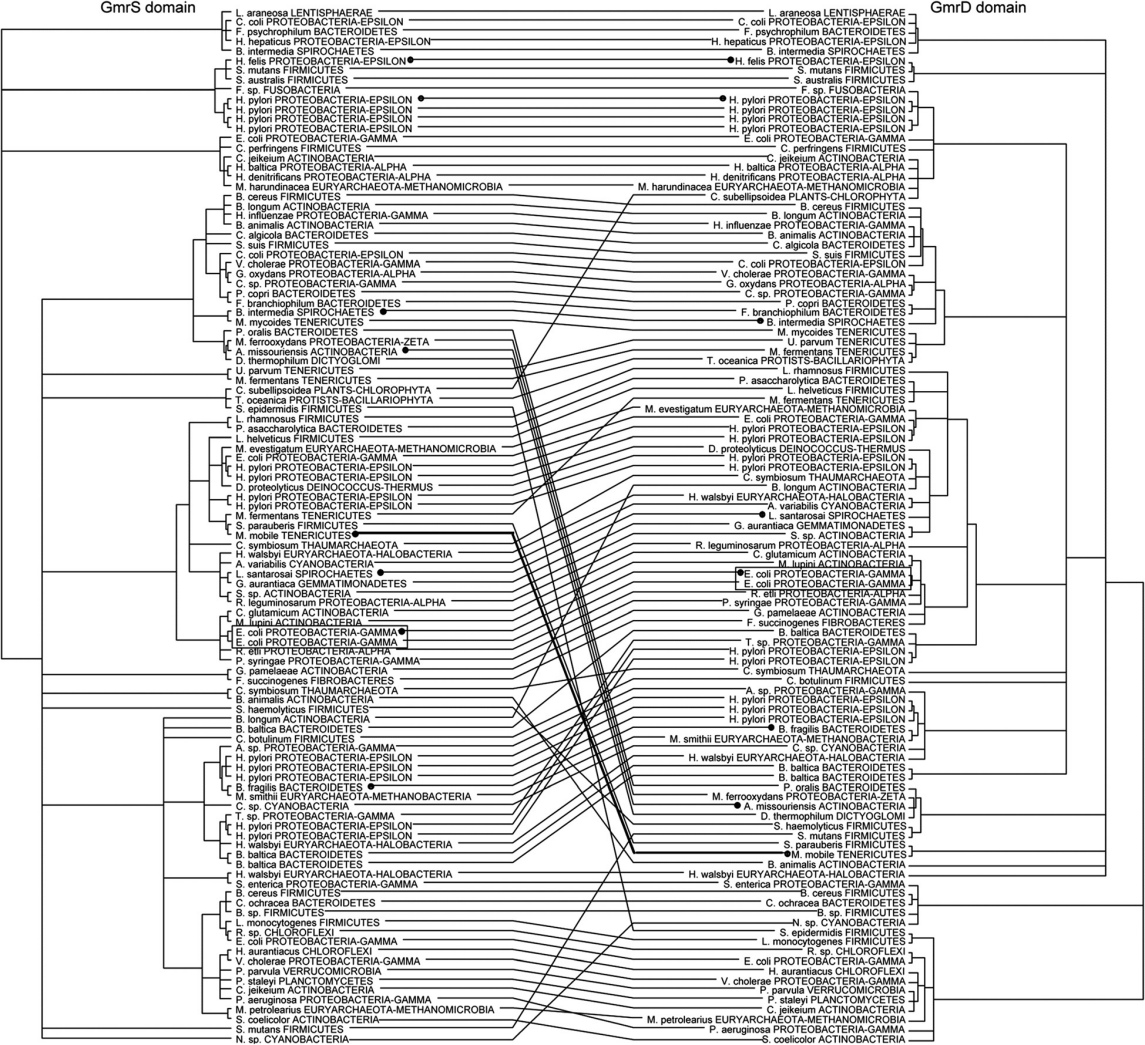
Phylogenetic trees calculated for sequences of GmrS and GmrD domains presented as a tanglegram. The associations were made either between domains from one double-domain protein or between single-domain proteins which may form a functional dimeric GmrSD system. Clades with SH-like support values below 80 % were collapsed. The dots indicate GmrS-GmrD pairs from the heterodimeric GmrSD system. The thick line shows the GmrSD system from *M. mobile* which is an example of possible horizontal gene transfer

To examine coevolution of GmrS and GmrD domains we have built two phylogenetic trees comprising GmrS and GmrD domain sequences from both double-domain and single-domain proteins, which we predict to form functional pairs (see Methods for details). We illustrated the coevolution between the domains by a tanglegram, in which domains derived from one double-domain protein or one predicted functional pair of single-domain proteins are linked (Fig. 7).

The tanglegram reveals very similar topologies of GmrS and GmrD phylogenetic trees, which suggests a coevolution of these two domains. In order to further support this observation we calculated a single phylogenetic tree for double-domain GmrSD proteins and merged GmrS-GmrD protein pairs (Additional file 9). The resulting topology is in good agreement with the individual GmrS and GmrD phylogenetic trees. However, the topologies of these trees do not agree with the universally accepted taxonomy of organisms [71], which suggests that evolution of GmrSD system involved frequent horizontal gene transfer events of the whole system. For example heterodimeric GmrSD system from *Mycoplasma mobile* (a fish pathogen), is most closely related to a double-domain GmrSD system from *Streptococcus parauberis* (etiological agent of mastitis in cows and streptococcosis in farmed fish). This indicates a horizontal transfer of the complete GmrSD enzyme between these two distantly related species dwelling in the same environment.

Interestingly, in some organisms the GmrSD system appears to be assembled from GmrS and GmrD domains acquired from distantly related organisms. For example, GmrS domain of the aforementioned *M. mobile* and *S. parauberis* groups together with GmrS domain of *Mycoplasma fermentas*. However the GmrD domain of *M. fermentas* is localized in a distant part of the tree together with *Lactobacillus helveticus* and *Porphyromonas asaccharolytica*. This suggests that GmrS and GmrD domains originating from distant organisms are “compatible” and may form functional systems.

We performed an analysis of positive and negative selection patterns in GmrSD proteins. Signatures of selection were detected in coding DNA sequences for three subgroups of GmrSD homologs using SLAC, FEL, REL [41] and MEME [42] methods implemented in the Datamonkey server [43] (see Methods section for details). As a result, we obtained a list of codons predicted to be under positive or negative selection. SLAC, FEL, REL methods reported large sets of codons under negative selection. These codons correspond to the majority of conserved residues characteristic for the GmrSD family. On the other hand, only few codons under positive selection have been reported and the majority of them were predicted by only one out of the four methods used. We conclude that for this dataset it is not possible to draw conclusions regarding the pattern of positive selection. Detailed results of this analysis are available as Additional file 6.

## Conclusions

We show that Type IV GmrSD is widespread among Prokaryotes and most often exists in the form of a single double-domain polypeptide. We propose that the GmrS domain has the ParB/Srx fold, while GmrD is an endonuclease from the HNH family. The residues of the highly conserved DGQQR motif in GmrS are most probably responsible for the NTP binding and hydrolysis, although they could also participate in DNA cleavage. The conserved E/D-H-N-N motif in GmrD is predicted to be responsible for the DNA cleavage and proper folding of the nuclease domain (see summarized information about conserved motifs in Table 2). GmrSD systems seem to be parts of the so called defense islands and may exist in mobile genetic elements. The GmrS and GmrD domains tend to coevolve, most probably because of their functional association, which makes the horizontal transfer of only one domain unlikely.

**Table 2:** Conserved motifs of the GmrSD proteins

Domain	Described motif	Homologous motif of known function (protein)	Predicted function
GmrS	QR	Conserved residues present in the DGQHR domain, unknown function	No function prediction
GmrS	I/VDGQQR	FG/SGCHR – signature sequence of sulfiredoxins	NTP binding, endonuclease
GmrS	FxxxN	Conserved residues present in the DGQHR domain, unknown function	No function prediction
GmrD	E/D-H-N-N	D-H-N-N in HNH endonuclease from *G. metallireducens*, E-H-N-N in the periplasmic nuclease Vvn	Endonuclease

After the submission of this manuscript a study of another member of the GmrSD family was published [72], which describes biochemical properties of the GmrSD enzyme from *E. coli* STEC_94 (Eco94GmrSD). Among other results it shows that the conserved D-H-N motif located at the C-terminus of the Eco94GmrSD protein is the potential endonuclease catalytic site (D507A, H508A and N522A variants exhibited no activity). These results partially confirm our predictions, since D507 and H508 of Eco94GmrSD are homologous to D113 and H144 of GmrD CT, which were predicted by us (in this work) to be involved in the catalysis. The influence of residues homologous to GmrD CT N169 and N178 was not studied. The authors did not propose a possible active site responsible for the NTPase activity and reported that they could not detect similarity to any known ATPase or GTPase. Hence, our predictions for GmrS provide additional information to guide further experimental studies. In particular our functional annotation of the DGQQR motif in GmrS remains to be experimentally verified.

## Additional files

Additional file 1:
**The alignment of GmrSD family members.** (FASTA 6123 kb)
Additional file 2:
**Interpretation and relevance of scores reported by the fold recognition methods used in this study.** (DOCX 18 kb)
Additional file 3:
**The alignment of GmrS domain sequences used for tree calculations.** (FASTA 38 kb)
Additional file 4:
**The alignment of GmrD domain sequences used for tree calculations.** (FASTA 35 kb)
Additional file 5:
**The alignment used for the calculations of the double-domain sequences tree.** (FASTA 53 kb)
Additional file 6:
**Results of the positive/negative selection patterns analysis.** (DOCX 139 kb)
Additional file 7:
**Results of protein-DNA docking and electrostatic potential analysis for GmrS and GmrD domains.** (PDF 671 kb)
Additional file 8:
**Results of the genomic context analysis.** (XLS 41 kb)
Additional file 9:
**The phylogenetic tree of double-domain GmrSD sequences**. Description of data: Phylogenetic tree calculated for sequences of double-domain GmrSD proteins and sigle-domain GmrS-GmrD pairs treated as one sequence. Clades with SH-like support values below 80 % were collapsed. The tree was calculated using approximated maximum-likelihood approach implemented in FastTree [67, 68]. (PDF 2577 kb)
