# Supplementary material for: Phylogenomics and sequence-structure-function relationships in the GmrSD family of Type IV restriction enzymes
Source: BMC Bioinformatics. 2015 Oct 23;16:336. doi: 10.1186/s12859-015-0773-z (PMC4619093; doi:10.1186/s12859-015-0773-z)
Supplement: Additional file 2: — Interpretation and relevance of scores reported by the fold recognition methods used in this study. (DOCX 18 kb) [file 12859_2015_773_MOESM2_ESM.docx]

**Supplementary table.** Interpretation and relevance of scores reported by fold recognition methods used in this study.

| **Method** | **Score interpretation** | **Score relevance** |
| --- | --- | --- |
| FFAS | Negative Z-score | Predictions with scores lower than **−9.5** contain <3% of false positives [[1](#_ENREF_1)]. |
| HHSEARCH | Probability for match to be a true positive (in % ) | Predictions with probability **>50%** can be considered relevant (according to the HHpred webserver help page). |
| COMA | E-value | Suggested relevance cut-off: **0.01** (false positives were shown to start accumulating above this value [[2](#_ENREF_2)]). |
| SAM_T08 | E-value | E-value <**0.01** indicate that a good structural template is available for at least part of the target protein [[3](#_ENREF_3)]. |

**References**

1. Jaroszewski L, Rychlewski L, Li Z, Li W, Godzik A: **FFAS03: a server for profile--profile sequence alignments**. *Nucleic acids research* 2005, **33**(Web Server issue):W284-288.

2. Margelevicius M, Venclovas C: **Detection of distant evolutionary relationships between protein families using theory of sequence profile-profile comparison**. *BMC bioinformatics* 2010, **11**:89.

3. Karplus K: **SAM-T08, HMM-based protein structure prediction**. *Nucleic acids research* 2009, **37**(Web Server issue):W492-497.
