# Supplementary material for: Phylogenomics and sequence-structure-function relationships in the GmrSD family of Type IV restriction enzymes
Source: BMC Bioinformatics. 2015 Oct 23;16:336. doi: 10.1186/s12859-015-0773-z (PMC4619093; doi:10.1186/s12859-015-0773-z)
Supplement: Additional file 6: — Results of the positive/negative selection patterns analysis. (DOCX 139 kb) [file 12859_2015_773_MOESM6_ESM.docx]

**Results of detecting signatures of selection from DNA sequences using SLAC, FEL, REL [**[**1**](#_ENREF_1)**] and MEME [**[**2**](#_ENREF_2)**] methods implemented in the Datamonkey server [**[**3**](#_ENREF_3)**]**

The analysis was conducted for 3 subgroups of GmrSD homologs.

In all tables cells marked in violet indicate that codon was reported to be non-neutral according to a given method at the specified significance level. The following significance levels were specified:

| SLAC p-value | 0.25 |
| --- | --- |
| FEL p-value | 0.1 |
| REL Bayes Factor | 50 |
| MEME p-value | 0.1 |

**Table 1.**

**Positively selected sites reported by at least one method for sequence group 1.**

| **Codon** | **SLAC dN-dS** | **SLAC p-value** | **FEL dN-dS** | **FEL p-value** | **REL dN-dS** | **REL Bayes Factor** | **MEME ω^+^** | **MEME p-value** |
| --- | --- | --- | --- | --- | --- | --- | --- | --- |
| 26 | 1.252 | 0.300 | 0.589 | 0.096 | -0.694 | 1.000 | >100 | 0.113 |
| 70 | 1.574 | 0.134 | 0.684 | 0.034 | -0.693 | 1.000 | >100 | 0.243 |
| 115 | 0.737 | 0.410 | 0.878 | 0.088 | -0.694 | 1.000 | >100 | 0.234 |
| 198 | 0.677 | 0.296 | 0.189 | 0.047 | -0.706 | 1.000 | >100 | 0.077 |
| 226 | 0.851 | 0.263 | 0.419 | 0.055 | -0.697 | 1.000 | 0.001 | 0.670 |
| 302 | 0.758 | 0.783 | 0.128 | 0.425 | -0.738 | 1.000 | >100 | 0.038 |
| 399 | 0.396 | 0.550 | 0.130 | 0.717 | -0.720 | 1.000 | >100 | 0.071 |
| 457 | 0.513 | 0.469 | 0.528 | 0.188 | -0.699 | 1.000 | >100 | 0.049 |
| 492 | 0.535 | 0.476 | 0.098 | 0.158 | -0.744 | 1.000 | >100 | 0.042 |
| 547 | 0.018 | 0.731 | 0.016 | 0.901 | -0.782 | 1.000 | >100 | 0.033 |
| 563 | 1.163 | 0.257 | 0.342 | 0.041 | -0.697 | 1.000 | >100 | 0.068 |
| 590 | 1.201 | 0.212 | 0.346 | 0.028 | -0.696 | 1.000 | >100 | 0.051 |
| 626 | -0.269 | 0.841 | -0.131 | 0.660 | -0.736 | 1.000 | >100 | 0.044 |
| 664 | -0.770 | 0.906 | -0.195 | 0.755 | -0.710 | 1.000 | >100 | 0.089 |
| 675 | 0.599 | 0.468 | 0.337 | 0.229 | -0.710 | 1.000 | >100 | 0.067 |
| 685 | 0.530 | 0.828 | 0.130 | 0.586 | -0.746 | 1.000 | >100 | 0.036 |
| 716 | 0.418 | 0.563 | 0.662 | 0.340 | -0.698 | 1.000 | >100 | 0.071 |

**Table 2.**

**Negatively selected sites reported by at least one method for sequence group 1.**

| **Codon** | **SLAC dN-dS** | **SLAC p-value** | **FEL dN-dS** | **FEL p-value** | **REL dN-dS** | **REL Bayes Factor** |
| --- | --- | --- | --- | --- | --- | --- |
| 24 | -0.911 | 0.401 | -2316.050 | 0.039 | -0.635 | 0.000 |
| 27 | -1.111 | 0.210 | -2316.940 | 0.013 | -0.738 | 0.000 |
| 29 | -0.374 | 0.547 | -16.596 | 0.073 | -0.630 | 0.000 |
| 32 | -0.649 | 0.465 | -15.351 | 0.031 | -0.665 | 0.000 |
| 37 | -1.742 | 0.109 | -0.378 | 0.136 | -0.908 | 0.000 |
| 38 | -2.950 | 0.090 | -2309.110 | 0.008 | -0.688 | 0.000 |
| 41 | -1.111 | 0.111 | -0.381 | 0.010 | -0.952 | 0.000 |
| 44 | -2.654 | 0.024 | -0.671 | 0.014 | -0.952 | 0.000 |
| 45 | -1.055 | 0.123 | -0.364 | 0.010 | -0.948 | 0.000 |
| 47 | -2.358 | 0.036 | -116.952 | 0.000 | -0.943 | 0.000 |
| 48 | -1.460 | 0.129 | -0.564 | 0.100 | -0.797 | 0.000 |
| 50 | -1.278 | 0.174 | -23.307 | 0.008 | -0.713 | 0.000 |
| 56 | -1.323 | 0.157 | -247.459 | 0.002 | -0.952 | 0.000 |
| 60 | -1.433 | 0.156 | -25.425 | 0.001 | -0.795 | 0.000 |
| 63 | -2.498 | 0.022 | -0.915 | 0.003 | -0.951 | 0.000 |
| 66 | -2.417 | 0.110 | -2309.150 | 0.043 | -0.622 | 0.000 |
| 69 | -0.309 | 0.555 | -2312.460 | 0.066 | -0.614 | 0.000 |
| 88 | -0.832 | 0.261 | -32.688 | 0.012 | -0.875 | 0.000 |
| 92 | -1.019 | 0.326 | -10.160 | 0.093 | -0.614 | 0.000 |
| 93 | -1.287 | 0.174 | -15.934 | 0.014 | -0.720 | 0.000 |
| 95 | -1.174 | 0.158 | -0.395 | 0.037 | -0.953 | 0.000 |
| 97 | -1.281 | 0.185 | -4.350 | 0.006 | -0.811 | 0.000 |
| 98 | -1.111 | 0.111 | -106.426 | 0.001 | -0.947 | 0.000 |
| 100 | -1.232 | 0.236 | -2302.900 | 0.009 | -0.670 | 0.000 |
| 101 | -1.481 | 0.097 | -6.909 | 0.011 | -0.820 | 0.000 |
| 120 | -1.113 | 0.166 | -427.607 | 0.005 | -0.950 | 0.000 |
| 121 | -1.111 | 0.111 | -60.830 | 0.003 | -0.947 | 0.000 |
| 123 | -1.324 | 0.156 | -0.612 | 0.028 | -0.952 | 0.000 |
| 124 | -1.576 | 0.044 | -9.501 | 0.001 | -0.947 | 0.000 |
| 125 | -0.615 | 0.427 | -3.480 | 0.074 | -0.626 | 0.000 |
| 126 | -1.111 | 0.111 | -0.347 | 0.016 | -0.951 | 0.000 |
| 127 | -1.666 | 0.037 | -0.678 | 0.002 | -0.951 | 0.000 |
| 128 | -1.388 | 0.111 | -0.749 | 0.041 | -0.836 | 0.000 |
| 129 | -1.324 | 0.156 | -0.612 | 0.028 | -0.952 | 0.000 |
| 132 | -1.119 | 0.176 | -0.821 | 0.053 | -0.796 | 0.000 |
| 134 | -1.111 | 0.210 | -1651.490 | 0.037 | -0.679 | 0.000 |
| 140 | -1.718 | 0.147 | 1146.910 | 1.000 | -0.617 | 0.000 |
| 141 | -2.230 | 0.121 | 189.948 | 1.000 | -0.613 | 0.000 |
| 150 | -1.186 | 0.218 | -24.169 | 0.042 | -0.617 | 0.000 |
| 151 | 0.228 | 0.703 | -23.000 | 0.100 | -0.612 | 0.000 |
| 153 | -2.077 | 0.060 | -44.871 | 0.005 | -0.657 | 0.000 |
| 158 | -0.599 | 0.361 | -31.858 | 0.048 | -0.805 | 0.000 |
| 160 | -3.193 | 0.013 | -127.749 | 0.000 | -0.953 | 0.000 |
| 165 | -1.335 | 0.280 | -6.650 | 0.053 | -0.654 | 0.000 |
| 175 | -2.654 | 0.022 | -0.671 | 0.014 | -0.952 | 0.000 |
| 202 | -0.446 | 0.528 | -20.719 | 0.094 | -0.614 | 0.000 |
| 205 | -1.524 | 0.284 | -2315.570 | 0.087 | -0.614 | 0.000 |
| 208 | -0.607 | 0.541 | -34.633 | 0.062 | -0.613 | 0.000 |
| 212 | -3.699 | 0.132 | -3.911 | 0.115 | -0.660 | 0.000 |
| 214 | -13.784 | 0.147 | -110.905 | 0.040 | -0.717 | 0.000 |
| 217 | -0.725 | 0.471 | -2312.330 | 0.030 | -0.616 | 0.000 |
| 220 | -2.199 | 0.153 | -6.679 | 0.143 | -0.613 | 0.000 |
| 223 | -1.000 | 0.362 | -25.674 | 0.012 | -0.705 | 0.000 |
| 224 | -0.833 | 0.259 | -0.393 | 0.090 | -0.891 | 0.000 |
| 247 | -0.896 | 0.333 | -5.861 | 0.072 | -0.854 | 0.000 |
| 283 | -1.536 | 0.090 | -27.424 | 0.001 | -0.881 | 0.000 |
| 289 | -1.320 | 0.171 | -3.836 | 0.012 | -0.742 | 0.000 |
| 292 | -1.929 | 0.120 | -0.505 | 0.325 | -0.663 | 0.000 |
| 295 | -1.057 | 0.193 | -0.351 | 0.145 | -0.824 | 0.000 |
| 299 | -1.384 | 0.129 | -0.872 | 0.003 | -0.927 | 0.000 |
| 303 | -2.489 | 0.022 | -0.916 | 0.003 | -0.951 | 0.000 |
| 304 | -2.489 | 0.022 | -0.916 | 0.003 | -0.951 | 0.000 |
| 306 | -2.654 | 0.024 | -0.671 | 0.014 | -0.952 | 0.000 |
| 308 | -1.564 | 0.056 | -0.887 | 0.003 | -0.949 | 0.000 |
| 309 | -2.489 | 0.022 | -0.916 | 0.004 | -0.950 | 0.000 |
| 310 | -1.977 | 0.082 | -0.443 | 0.118 | -0.900 | 0.000 |
| 312 | -0.781 | 0.282 | -0.984 | 0.005 | -0.931 | 0.000 |
| 313 | -1.170 | 0.158 | -0.442 | 0.038 | -0.952 | 0.000 |
| 316 | -1.219 | 0.198 | -6.647 | 0.025 | -0.690 | 0.000 |
| 319 | -0.617 | 0.397 | -1.344 | 0.002 | -0.926 | 0.000 |
| 321 | -1.388 | 0.111 | -33.715 | 0.006 | -0.890 | 0.000 |
| 323 | -2.486 | 0.022 | -0.651 | 0.015 | -0.951 | 0.000 |
| 324 | -1.011 | 0.134 | -0.465 | 0.013 | -0.939 | 0.000 |
| 326 | -0.264 | 0.555 | -1.264 | 0.075 | -0.843 | 0.000 |
| 327 | -1.603 | 0.115 | -0.484 | 0.030 | -0.953 | 0.000 |
| 330 | -2.498 | 0.022 | -0.915 | 0.004 | -0.950 | 0.000 |
| 333 | -1.386 | 0.113 | -0.631 | 0.030 | -0.880 | 0.000 |
| 344 | -1.854 | 0.027 | -0.955 | 0.002 | -0.932 | 0.000 |
| 345 | -2.836 | 0.058 | -0.591 | 0.121 | -0.855 | 0.000 |
| 352 | -1.170 | 0.158 | -0.442 | 0.045 | -0.950 | 0.000 |
| 370 | -2.443 | 0.059 | -0.902 | 0.030 | -0.903 | 0.000 |
| 371 | -2.413 | 0.063 | -40.553 | 0.003 | -0.882 | 0.000 |
| 372 | -1.552 | 0.165 | -0.412 | 0.681 | -0.615 | 0.000 |
| 373 | -0.589 | 0.393 | -0.905 | 0.049 | -0.859 | 0.000 |
| 375 | -1.111 | 0.121 | -0.564 | 0.010 | -0.947 | 0.000 |
| 381 | -0.876 | 0.213 | -0.196 | 0.039 | -0.946 | 0.000 |
| 385 | -1.433 | 0.104 | -12.204 | 0.003 | -0.835 | 0.000 |
| 395 | -0.913 | 0.293 | -23.796 | 0.027 | -0.647 | 0.000 |
| 397 | -1.977 | 0.085 | -0.510 | 0.121 | -0.875 | 0.000 |
| 427 | -0.884 | 0.251 | -19.073 | 0.003 | -0.932 | 0.000 |
| 430 | -1.547 | 0.192 | -0.696 | 0.154 | -0.686 | 0.000 |
| 434 | -2.935 | 0.056 | -1.922 | 0.043 | -0.846 | 0.000 |
| 435 | -1.370 | 0.147 | -0.374 | 0.040 | -0.951 | 0.000 |
| 440 | -0.965 | 0.226 | -2316.420 | 0.080 | -0.800 | 0.000 |
| 451 | -1.462 | 0.174 | -0.342 | 0.342 | -0.693 | 0.000 |
| 462 | -1.136 | 0.111 | -290.686 | 0.005 | -0.950 | 0.000 |
| 465 | -0.568 | 0.333 | -0.146 | 0.082 | -0.951 | 0.000 |
| 466 | -1.132 | 0.175 | -2316.910 | 0.051 | -0.784 | 0.000 |
| 496 | -1.034 | 0.130 | -0.210 | 0.031 | -0.946 | 0.000 |
| 497 | -1.331 | 0.073 | -1.061 | 0.003 | -0.944 | 0.000 |
| 500 | -1.612 | 0.208 | -1.601 | 0.080 | -0.727 | 0.000 |
| 501 | -1.111 | 0.113 | -0.418 | 0.014 | -0.947 | 0.000 |
| 504 | -1.388 | 0.111 | -0.589 | 0.025 | -0.906 | 0.000 |
| 505 | 0.020 | 0.432 | -2317.040 | 0.055 | -0.855 | 0.000 |
| 507 | -1.606 | 0.147 | -0.506 | 0.046 | -0.943 | 0.000 |
| 515 | -1.147 | 0.162 | -1.064 | 0.049 | -0.796 | 0.000 |
| 516 | -1.843 | 0.139 | -17.073 | 0.020 | -0.711 | 0.000 |
| 538 | -0.555 | 0.410 | -1.008 | 0.060 | -0.795 | 0.000 |
| 539 | -1.281 | 0.172 | -0.943 | 0.170 | -0.663 | 0.000 |
| 553 | -1.111 | 0.111 | -0.565 | 0.006 | -0.952 | 0.000 |
| 554 | -0.791 | 0.356 | -2295.550 | 0.081 | -0.613 | 0.000 |
| 555 | -2.411 | 0.024 | -0.661 | 0.014 | -0.951 | 0.000 |
| 568 | -1.684 | 0.141 | -0.476 | 0.047 | -0.943 | 0.000 |
| 580 | -1.183 | 0.217 | -2308.290 | 0.083 | -0.733 | 0.000 |
| 581 | -1.499 | 0.057 | -0.732 | 0.005 | -0.926 | 0.000 |
| 584 | -0.630 | 0.379 | -1.017 | 0.056 | -0.800 | 0.000 |
| 606 | -1.400 | 0.065 | -0.863 | 0.009 | -0.933 | 0.000 |
| 610 | -1.174 | 0.158 | -0.395 | 0.037 | -0.953 | 0.000 |
| 613 | -1.111 | 0.111 | -0.561 | 0.007 | -0.952 | 0.000 |
| 614 | -1.258 | 0.277 | -0.867 | 0.089 | -0.890 | 0.000 |
| 621 | -1.380 | 0.117 | -0.541 | 0.048 | -0.851 | 0.000 |
| 624 | -0.949 | 0.227 | -0.645 | 0.049 | -0.904 | 0.000 |
| 625 | -1.302 | 0.126 | -0.412 | 0.085 | -0.818 | 0.000 |
| 631 | -1.448 | 0.333 | -0.506 | 0.066 | -0.836 | 0.000 |
| 653 | -2.193 | 0.108 | -0.916 | 0.101 | -0.720 | 0.000 |
| 659 | -0.847 | 0.258 | -0.659 | 0.007 | -0.945 | 0.000 |
| 664 | -1.659 | 0.183 | -0.237 | 0.598 | -0.629 | 0.000 |
| 665 | -0.018 | 0.635 | -19.315 | 0.065 | -0.613 | 0.000 |
| 667 | -1.111 | 0.121 | -0.150 | 0.091 | -0.947 | 0.000 |
| 668 | -2.498 | 0.022 | -0.915 | 0.003 | -0.952 | 0.000 |
| 669 | -0.838 | 0.260 | -0.711 | 0.008 | -0.933 | 0.000 |
| 670 | -1.111 | 0.111 | -0.501 | 0.014 | -0.951 | 0.000 |
| 671 | -0.835 | 0.275 | -0.799 | 0.012 | -0.926 | 0.000 |
| 673 | -0.890 | 0.240 | -0.586 | 0.064 | -0.903 | 0.000 |
| 679 | -0.808 | 0.364 | -2316.520 | 0.078 | -0.618 | 0.000 |
| 682 | -1.170 | 0.158 | -0.442 | 0.038 | -0.952 | 0.000 |
| 699 | -1.340 | 0.147 | -0.320 | 0.042 | -0.952 | 0.000 |
| 700 | -1.055 | 0.127 | -0.364 | 0.010 | -0.948 | 0.000 |
| 709 | -0.873 | 0.180 | -0.449 | 0.019 | -0.937 | 0.000 |
| 712 | -1.603 | 0.115 | -0.484 | 0.030 | -0.953 | 0.000 |
| 713 | -0.981 | 0.276 | -39.509 | 0.059 | -0.616 | 0.000 |
| 737 | -1.150 | 0.263 | -0.713 | 0.100 | -0.911 | 0.000 |
| 740 | -1.662 | 0.050 | -65.907 | 0.001 | -0.949 | 0.000 |
| 743 | -1.390 | 0.112 | -15.835 | 0.000 | -0.945 | 0.000 |
| 747 | -1.380 | 0.129 | -1.221 | 0.002 | -0.925 | 0.000 |
| 754 | -1.165 | 0.169 | -0.402 | 0.176 | -0.796 | 0.000 |
| 756 | -1.111 | 0.111 | -0.385 | 0.010 | -0.952 | 0.000 |
| 757 | -1.096 | 0.249 | -1.387 | 0.028 | -0.834 | 0.000 |
| 763 | -0.896 | 0.333 | -5.520 | 0.041 | -0.843 | 0.000 |
| 767 | -0.896 | 0.333 | -27.999 | 0.041 | -0.843 | 0.000 |

**Multiple sequence alignment for sequences from group 1.** Positions predicted to be under positive or negative selection by at least two methods are indicated in the first two rows.

10 20 30 40 50 60 70 80 90 100

....|....|....|....|....|....|....|....|....|....|....|....|....|....|....|....|....|....|....|....|

**Positive_selection**  **---------------------------------------------------------------------P------------------------------** 1

**Negative_selection**  **-------------------------------------N--N--NN-N--N-----N---N--N--N--------------------------N-N-NN--** 14

**GmrSD|gi|218559591|E** **MIVLNQINSSLLYNVAYRYTMKSETLTVQQLFQDRRQYCVPFYQRAYVWTQ--QDQWSALLEDILEKVQSRL--------------SGTKPTP-HFLGAV** 83

**GmrSD|gi|28867280|P_** **--------------------MQPSKTTVTGLFNSPHQYQIPIFQRGYVWTL--EKQVGPLWADIQDRADAVL---EQHSLSSTGTNAGLKPLQKHFLGSI** 75

**GmrSD|gi|327188766|R** **--------------------MRADAYPLEIVLSERQQWVVPVYQRHYEWETGEDRQLPKFWADLEDEAVDRL--------------EGRSPFP-HYFGAI** 65

**GmrSD|gi|344045394|C** **--------------------MEADTKALKKIMTVEGRYVIPTFQRDYEWTR--EGQWELLFEDLDSVAGRLGAARVEAEATGSSQARAERSVAPHFLGAV** 78

**GmrS|gi|21327769|Gmr** **--------------------MKSETLTIQQIFQNQRQYRVPFYQRAYVWTQ--RNQWSALLEDIFEKAQSRL--------------SGTKPTP-HFLGAV** 63

110 120 130 140 150 160 170 180 190 200

....|....|....|....|....|....|....|....|....|....|....|....|....|....|....|....|....|....|....|....|

**Positive_selection/1** **-------------------------------------------------------------------------------------------------P--** 2

**Negative_selection/1** **N------------------NN-NN-NNNN--N--------------------N------N--------------N-------------------------** 27

**GmrSD|gi|218559591|E** **VLEPQSKKGLLGVDSIHIIDGQQRLTTLQYVLASIRLALRATDLSS---LEALISPCLKNSNEDTMRNKEVERFKLWPTFRDQTHFIQSFNVENIDDLRD** 180

**GmrSD|gi|28867280|P_** **VMTPVP-NSFGRVAAFEVIDGQQRSTTLQLLLLAFRQAALELPGSP---IPQMLNGLVRNPGPY---QCAEDHYKIWPTQAGQDEMRRLSDAATDAQVIC** 168

**GmrSD|gi|327188766|R** **IFSEPKGQAFGVVRKRYLVDGQQRITTFQLVLAAIREVARRREVAR---LVDVLNAYLFNEKSASMLDPDRERLKLWPSSYDRKLYQDIAENTPAKIRSL** 162

**GmrSD|gi|344045394|C** **VCDQLP-SPTGGLTLSAVIDGQQRLTTLQLLVRGVLDVLQETGSGR---ARQVKR-LLENPEDVI--DHPHERYKLWPRRKDRDVWPVAMSDEVPAYG--** 169

**GmrS|gi|21327769|Gmr** **VLEPQLKNSLLGVDTIHIIDGQQRLTTLQYILASLASIRLSLRATGLSELEGLVLTCLKNTNEATMRNKKVECFKLWPTFRDQTHFIQSLNVDNIDDLRN** 163

210 220 230 240 250 260 270 280 290 300

....|....|....|....|....|....|....|....|....|....|....|....|....|....|....|....|....|....|....|....|

**Positive_selection/1** **----------------------------------------------------------------------------------------------------** 3

**Negative_selection/1** **-------------N--------------------------------------------------------------------N-----N---------N-** 31

**GmrSD|gi|218559591|E** **VFSDSFTQHGTLRKHFNHPPSLEALCFFTEAFIKWIKIE----------------------------------NHSPQENAVALIEAVLTDLKLVSIFLE** 246

**GmrSD|gi|28867280|P_** **NGYPVRDGRV----RIERPLMIQTYLYLYHACLAYMRGVDLTDAVNPASDATLGDTLIADIRRHNDVLPLQAGLPLISSRGEALYMALSDLMQIMTLTLE** 264

**GmrSD|gi|327188766|R** **QSKFFYKNGALI--KGQAPNLLRAYMFLLDAIDEFVTERGTLQNETP------------------------------EAVLSAVVAGFLSGFQIVVIQLD** 230

**GmrSD|gi|344045394|C** **--------------TEDHLYL-QARRFFADSVRNSLVDDQGRD------------------------------------RIDDFVDALLDLFKLVVIDLD** 218

**GmrS|gi|21327769|Gmr** **VFSDSFTQHGTLRKHFNHPPSLEALWFFTEAFIKWIKIE----------------------------------NHSPQENAVALIEAVLTDLKLVSIFLE** 229

310 320 330 340 350 360 370 380 390 400

....|....|....|....|....|....|....|....|....|....|....|....|....|....|....|....|....|....|....|....|

**Positive_selection/1** **----------------------------------------------------------------------------------------------------** 3

**Negative_selection/1** **--NN-N-NN---N--N----N-NN--N--N--N----------N-------N-----------------NN---N---------N---------------** 50

**GmrSD|gi|218559591|E** **AEDDAQIIFETLNGRGAELHATDLIRNYIFMCAEH-ENINAIELYENEWKSFE-----------DKYWSEKQRRGRINKPRMEWLVHATLQSERQREIDL** 334

**GmrSD|gi|28867280|P_** **AEDDPQIIFETLNARGEPLLASDLVRNYVFLEAAR-QGKDVPTLYESYWSPFDLVANSGKGVSTNAYWREKERQGRLTYPRIDLFFYNYATLRSQEVTLA** 363

**GmrSD|gi|327188766|R** **ENDDAQEIFASLNGLGKPLSPFDLIRNDVFHRARK-TGEDDQKLFDERWKLFE-----------QPFWTEQVRQGRFKRARADHLVSHVVIAETGREANI** 318

**GmrSD|gi|344045394|C** **DNDDAQVIFEVLNGRQTPLSASDLVKNLLFLRGELADEQELEELYDAYWAEFD-----------EPWWKKQIGVGHAARARRDVLLSVWLTAVSGTEANV** 307

**GmrS|gi|21327769|Gmr** **AEDDAQIIFETLNGRGAELHATDLIRNYIFMCAEH-ENINAIELYENEWKIFE-----------DKYWSEKQRRGRINKPRMEWLVHATLQSERQQ----** 313

410 420 430 440 450 460 470 480 490 500

....|....|....|....|....|....|....|....|....|....|....|....|....|....|....|....|....|....|....|....|

**Positive_selection/1** **----------------------------------------------------------------------------------------------------** 3

**Negative_selection/1** **---------------------------------NN--------------------------N---N-----------------------------NN---** 56

**GmrSD|gi|218559591|E** **SRLYNEYRDYVSKDLSSQRADLQVKRLKQYASQYKEL--VDGFGTTPISHFGYRIADYDVTTLYPLALFISIANI---ADDEKAAMYNDLVSYVVRRAVC** 429

**GmrSD|gi|28867280|P_** **SHVFQSFKTWWQKQP--RDLEAELGRLVASSTHFKEL--VSPEGTGYLAEFARLVRSLDVSTVTPVYLALRERLDK--DSTELKQALSDLASYLTRRTVC** 457

**GmrSD|gi|327188766|R** **GKIATEYQRYARERGF-QTVGEELDVLIGHAATYKDM--ERLTPGTLTHRIATVLRIWDLSTFHPLILAINGLTL---EDERKTELFKLLESYIVRREIC** 412

**GmrSD|gi|344045394|C** **GRLYGEVRQYLAGGD--HKTKDILIELSEYRQAYKAIYGVLDSGSPRLAQSYRNLVILKLQTAVPLLAWLRTLPAGRLSLADHERAAGAVESWVLRRMIL** 405

**GmrS|gi|21327769|Gmr** **-------------------------------------------------------------------------------------MYNDLVSYVVRRSVC** 328

510 520 530 540 550 560 570 580 590 600

....|....|....|....|....|....|....|....|....|....|....|....|....|....|....|....|....|....|....|....|

**Positive_selection/1** **--------------------------------------------------------------P--------------------------P----------** 5

**Negative_selection/1** **N--N--N-------NN------------------------------------N-N------------N------------N-------------------** 65

**GmrSD|gi|218559591|E** **GLTPKNYNNVFMNVLRHLAKTEISSV----------ELRNILNNLNGEASRWPGDSEFLNACINAPLYPGRLDAPKMRSMLTELERELCRQVKTEKPDVP** 519

**GmrSD|gi|28867280|P_** **GSTTKGYNRFFMRVLQSVSAASIDPHL---------ALRETLLAATGTSEIWPDDASFSDKWCHRAVYKELRPVK-TCGVLRALEYAARGS-QQASNHVP** 546

**GmrSD|gi|327188766|R** **GLTTKNYNKVVIGCVKEVRKEGDPVA----------ALRKHISELTGEASRMPTDLEVAEAFARRRSYGVIPTPR-LRYILEQLEYGSRTK-F--DEVTV** 498

**GmrSD|gi|344045394|C** **GANTRGYNAAFLGVLKSAQAAARTPEANIAD-----AVVAALAAS-PNSLAWPSDDEITSAFTQDRFYGRFTQER-IRLILGAIDAQMRVNNPKTEPAVF** 498

**GmrS|gi|21327769|Gmr** **GLTPKNYNNVFMNVLRHLSKTEISSV----------ELRNILNSLNGEASRWPGDSEFLNACINAPLYPGRLDAPKMRSMLTELERELCRQVKTEKPDVP** 418

610 620 630 640 650 660 670 680 690 700

....|....|....|....|....|....|....|....|....|....|....|....|....|....|....|....|....|....|....|....|

**Positive_selection/1** **----------------------------------------------------------------------------------------------------** 6

**Negative_selection/1** **-----N---N--N-------N---N-----------------------------------------NN-N-----------N----------------NN** 76

**GmrSD|gi|218559591|E** **NL-SNLDIDHLMPQS-WY--SCWPLENGRMVTNSDATVLNQIVLSGTDLTPEQLLVRKRQQAISTLGNLTLLNLSVNRSVQNAVFLK-----------KR** 604

**GmrSD|gi|28867280|P_** **AQ-SDLTVEHVLPQS-WKNLSYYQIESMT-----------------------EGQSQLRDIAVHGFGNLTLLTQPLNSSISNGPFTDTIGVDGELVLGKR** 621

**GmrSD|gi|327188766|R** **AT-DNLTVEHVMPRK-WA--PHWPLPNGTTVPV--ESTFEATIGNHSLTDEQKALMDTRQRSIDTLGNLTLLTEALNPSIGNGKWEA-----------KR** 581

**GmrSD|gi|344045394|C** **DY-AQLQIEHLMPQR-WE--PHWPLPASVG----------------EDPAQRELATSERSAAVDQIGNLTLVTSAFNQGVSNLGWDQ-----------KR** 567

**GmrS|gi|21327769|Gmr** **NL-SNLDIDHLMPQSW-Y--SCWPLENGHMVTNSDATVMNQIVLSGTDLTPEQLLVRKRQQAIATLGNLTLLNLSVNRSVQNAVFLK-----------KR** 503

710 720 730 740 750 760 770 780

....|....|....|....|....|....|....|....|....|....|....|....|....|....|....|....|....|...

**Positive_selection/1** **----------------------------------------------------------------------------------------** 6

**Negative_selection/1** **--------N--N---------------------------N--N---N--------N--------------------------------** 82

**GmrSD|gi|218559591|E** **DALIVHTNLRLNIPLIVKDK--------------WDEDEILERGKKLGEIALKVWPKHD-----------------------------** 649

**GmrSD|gi|28867280|P_** **SRLGQSALLL-NTYFHQSALAS------------WDDVAIENRAKALLKAALLVWPKPVDSTASPVAVASAISRCPDRIVRLFDKEGV** 696

**GmrSD|gi|327188766|R** **EKIA-KSLLALNREIAAVDA--------------WSETEIGARATRLSVTSNRIWPRGQQAPAVTSALSSG-----------------** 637

**GmrSD|gi|344045394|C** **PELAAQSALQLNLPIATTEH--------------WDEETITARGEALADIACRIWPR-------------------------------** 610

**GmrS|gi|21327769|Gmr** **DALIVHTNLRLNIPLILKDK--------------WDESEIQERGKKLGEIALKVWPKYD-----------------------------** 548

**Table 3.**

**Positively selected sites reported by at least one method for sequence group 2.**

| **Codon** | **SLAC dN-dS** | **SLAC p-value** | **FEL dN-dS** | **FEL p-value** | **REL dN-dS** | **REL Bayes Factor** | **MEME ω^+^** | **MEME p-value** |
| --- | --- | --- | --- | --- | --- | --- | --- | --- |
| 60 | 0.321 | 0.631 | 0.143 | 0.409 | -0.720 | 0.087 | >100 | 0.056 |
| 195 | 0.603 | 0.384 | 1.161 | 0.078 | -0.036 | 27.728 | >100 | 0.113 |
| 331 | 0.033 | 0.633 | 537.385 | 1.000 | 0.132 | 376.207 | >100 | 0.651 |
| 340 | 0.486 | 0.478 | 0.315 | 0.219 | -0.633 | 0.911 | >100 | 0.084 |
| 378 | -0.577 | 0.945 | -1.608 | 0.446 | -0.621 | 1.037 | >100 | 0.072 |
| 420 | 0.338 | 0.510 | 4.987 | 0.191 | -0.122 | 16.639 | >100 | 0.019 |
| 458 | 0.165 | 0.607 | 0.246 | 0.512 | -0.714 | 0.128 | >100 | 0.097 |
| 484 | 0.403 | 0.410 | 3.048 | 0.323 | 0.057 | 63.814 | >100 | 0.239 |
| 496 | 0.093 | 0.654 | 0.362 | 0.344 | -0.676 | 0.470 | >100 | 0.037 |
| 533 | 1.128 | 0.308 | 1.966 | 0.565 | 0.088 | 99.636 | 0.003 | 0.670 |
| 534 | 1.665 | 0.155 | 2.153 | 0.064 | 0.009 | 39.272 | >100 | 0.227 |
| 535 | 1.071 | 0.332 | 2.116 | 0.477 | 0.045 | 55.518 | >100 | 0.190 |
| 624 | 0.116 | 0.648 | 0.223 | 0.395 | -0.703 | 0.239 | >100 | 0.075 |
| 638 | -0.030 | 0.704 | -0.886 | 0.376 | -0.685 | 0.388 | >100 | 0.079 |
| 641 | 0.068 | 0.641 | 1031.110 | 1.000 | 0.105 | 140.532 | 0.208 | 0.670 |
| 734 | 0.819 | 0.189 | 956.834 | 1.000 | 0.126 | 277.278 | 0.051 | 0.670 |
| 764 | 0.612 | 0.199 | 0.351 | 0.230 | -0.650 | 0.730 | >100 | 0.269 |

**Table 4.**

**Negatively selected sites reported by at least one method for sequence group 2.**

| Codon | SLAC dN-dS | SLAC p-value | FEL dN-dS | FEL p-value | REL dN-dS | REL Bayes Factor |
| --- | --- | --- | --- | --- | --- | --- |
| 43 | 0.000 | 0.000 | 0.000 | 1.000 | -0.976 | 15770.600 |
| 45 | -0.681 | 0.111 | -0.924 | 0.005 | -0.963 | 2107.090 |
| 47 | -0.596 | 0.363 | -1699.940 | 0.041 | -0.702 | 4.114 |
| 50 | 0.000 | 0.000 | 0.000 | 1.000 | -0.914 | 208.967 |
| 61 | -1.301 | 0.041 | -0.893 | 0.019 | -0.952 | 2039.110 |
| 62 | -1.243 | 0.033 | -1.098 | 0.006 | -0.952 | 2463.830 |
| 64 | -0.353 | 0.447 | -1708.750 | 0.014 | -0.719 | 11.769 |
| 65 | -0.681 | 0.111 | -4.514 | 0.009 | -0.970 | 5052.780 |
| 67 | -0.608 | 0.219 | -0.372 | 0.072 | -0.925 | 757.137 |
| 68 | -0.628 | 0.209 | -0.988 | 0.031 | -0.952 | 2057.640 |
| 69 | -0.840 | 0.114 | -1.718 | 0.001 | -0.953 | 545.293 |
| 71 | -0.608 | 0.219 | -0.372 | 0.072 | -0.925 | 757.137 |
| 73 | 0.000 | 0.000 | 0.000 | 1.000 | -0.939 | 8051.270 |
| 76 | -1.003 | 0.160 | -0.817 | 0.219 | -0.713 | 7.055 |
| 78 | -0.544 | 0.327 | -0.453 | 0.242 | -0.779 | 277.011 |
| 80 | 0.163 | 0.199 | 0.066 | 0.493 | -0.763 | 64.527 |
| 81 | -0.460 | 0.296 | -1.582 | 0.003 | -0.920 | 112.306 |
| 82 | 0.000 | 0.000 | 0.000 | 1.000 | -0.939 | 8051.270 |
| 83 | -0.816 | 0.190 | -0.546 | 0.279 | -0.723 | 17.890 |
| 84 | 0.000 | 0.000 | 0.000 | 1.000 | -0.957 | 1291.740 |
| 87 | -1.010 | 0.079 | -0.987 | 0.087 | -0.758 | 18.520 |
| 89 | -0.654 | 0.287 | -1476.420 | 0.095 | -0.628 | 1.045 |
| 95 | -1.624 | 0.094 | -1704.080 | 0.112 | -0.554 | 0.545 |
| 97 | -0.608 | 0.186 | -0.372 | 0.050 | -0.966 | 2717.650 |
| 98 | -0.608 | 0.186 | -0.372 | 0.061 | -0.950 | 2497.860 |
| 100 | 0.000 | 0.000 | 0.000 | 1.000 | -0.956 | 1758.950 |
| 101 | -0.681 | 0.118 | -0.860 | 0.009 | -0.943 | 1984.640 |
| 103 | -0.253 | 0.485 | -1.288 | 0.079 | -0.734 | 8.492 |
| 104 | 0.170 | 0.240 | 0.084 | 0.448 | -0.748 | 59.391 |
| 121 | -0.678 | 0.182 | -0.722 | 0.069 | -0.757 | 52.240 |
| 122 | -1.282 | 0.031 | -1.069 | 0.005 | -0.961 | 1506.900 |
| 123 | -0.340 | 0.333 | -0.186 | 0.098 | -0.956 | 1724.420 |
| 124 | -1.300 | 0.041 | -1.016 | 0.007 | -0.952 | 1982.030 |
| 125 | -0.631 | 0.208 | -0.721 | 0.046 | -0.952 | 2108.410 |
| 126 | -0.862 | 0.062 | -1.679 | 0.002 | -0.952 | 558.148 |
| 128 | -0.681 | 0.111 | -0.726 | 0.010 | -0.963 | 2009.990 |
| 129 | -0.681 | 0.111 | -4.515 | 0.011 | -0.963 | 2082.580 |
| 132 | -0.768 | 0.088 | -1.710 | 0.003 | -0.916 | 144.357 |
| 133 | -1.182 | 0.035 | -0.687 | 0.016 | -0.901 | 128.557 |
| 136 | -0.681 | 0.111 | -4.512 | 0.003 | -0.963 | 2194.160 |
| 137 | -0.895 | 0.079 | -0.540 | 0.021 | -0.897 | 133.251 |
| 141 | -0.735 | 0.100 | -4.040 | 0.001 | -0.919 | 157.242 |
| 153 | -0.781 | 0.206 | -0.625 | 0.361 | -0.712 | 6.502 |
| 157 | -1.000 | 0.162 | -0.865 | 0.165 | -0.715 | 8.024 |
| 158 | -0.480 | 0.322 | -1708.450 | 0.011 | -0.711 | 4.193 |
| 160 | -0.943 | 0.159 | -15.753 | 0.025 | -0.720 | 11.536 |
| 162 | -1.629 | 0.030 | -83.759 | 0.001 | -0.925 | 829.508 |
| 163 | -0.768 | 0.088 | -3.647 | 0.002 | -0.915 | 155.042 |
| 165 | -0.616 | 0.184 | -0.786 | 0.039 | -0.959 | 1432.930 |
| 175 | -0.657 | 0.303 | -0.788 | 0.231 | -0.748 | 50.423 |
| 177 | -0.709 | 0.188 | -1.160 | 0.007 | -0.904 | 98.816 |
| 179 | -0.972 | 0.048 | -345.945 | 0.000 | -0.914 | 98.594 |
| 183 | -0.598 | 0.190 | -0.739 | 0.041 | -0.958 | 1298.020 |
| 199 | -1.225 | 0.163 | -1708.670 | 0.024 | -0.701 | 4.214 |
| 214 | 0.140 | 0.178 | 0.063 | 0.497 | -0.765 | 69.198 |
| 215 | -1.243 | 0.033 | -1.098 | 0.006 | -0.952 | 2463.830 |
| 219 | -0.853 | 0.131 | -0.995 | 0.054 | -0.752 | 29.466 |
| 222 | -0.988 | 0.237 | -12.503 | 0.178 | -0.487 | 0.356 |
| 226 | -1.070 | 0.177 | -0.701 | 0.430 | -0.663 | 1.675 |
| 227 | -0.884 | 0.080 | -0.502 | 0.028 | -0.899 | 160.596 |
| 232 | -0.422 | 0.450 | -1708.750 | 0.053 | -0.645 | 1.272 |
| 233 | -0.665 | 0.228 | -3.939 | 0.061 | -0.714 | 7.244 |
| 236 | -0.509 | 0.392 | -1708.920 | 0.018 | -0.742 | 14.355 |
| 237 | -1.025 | 0.039 | -1.223 | 0.005 | -0.914 | 143.034 |
| 238 | -1.883 | 0.217 | -1707.170 | 0.379 | -0.672 | 1.964 |
| 239 | -0.921 | 0.153 | -1.642 | 0.023 | -0.753 | 41.535 |
| 240 | -1.021 | 0.037 | -1.001 | 0.004 | -0.931 | 361.723 |
| 242 | -1.251 | 0.098 | -2.193 | 0.049 | -0.737 | 12.031 |
| 244 | -0.761 | 0.095 | -1.603 | 0.003 | -0.914 | 116.652 |
| 247 | -0.434 | 0.379 | -1703.910 | 0.049 | -0.677 | 2.160 |
| 249 | -0.608 | 0.186 | -0.372 | 0.055 | -0.958 | 1288.530 |
| 250 | -0.965 | 0.155 | -0.943 | 0.118 | -0.724 | 17.933 |
| 254 | -0.425 | 0.381 | -1.533 | 0.041 | -0.751 | 38.133 |
| 255 | -0.608 | 0.186 | -0.372 | 0.061 | -0.950 | 2497.860 |
| 256 | -0.651 | 0.184 | -0.660 | 0.047 | -0.955 | 1088.880 |
| 257 | -1.282 | 0.031 | -1.069 | 0.004 | -0.974 | 10761.400 |
| 258 | -0.330 | 0.243 | -0.112 | 0.807 | -0.723 | 16.906 |
| 259 | 0.000 | 0.000 | 0.000 | 1.000 | -0.966 | 2230.830 |
| 260 | -0.735 | 0.199 | -0.656 | 0.185 | -0.719 | 11.324 |
| 261 | -0.573 | 0.229 | -1.035 | 0.074 | -0.768 | 28.698 |
| 262 | -0.340 | 0.333 | -0.171 | 0.103 | -0.956 | 1734.020 |
| 265 | -1.015 | 0.092 | -1.289 | 0.004 | -0.905 | 98.772 |
| 267 | -1.301 | 0.041 | -0.893 | 0.019 | -0.952 | 2039.110 |
| 269 | -0.608 | 0.186 | -0.372 | 0.055 | -0.958 | 1288.530 |
| 270 | -0.873 | 0.073 | -0.430 | 0.033 | -0.905 | 159.341 |
| 272 | -1.365 | 0.016 | -1.730 | 0.002 | -0.951 | 430.761 |
| 273 | -0.819 | 0.139 | -0.876 | 0.033 | -0.963 | 1661.830 |
| 274 | -0.916 | 0.193 | -0.841 | 0.259 | -0.703 | 4.423 |
| 275 | -0.721 | 0.247 | -0.959 | 0.156 | -0.712 | 6.566 |
| 277 | 0.000 | 0.000 | 0.000 | 1.000 | -0.976 | 15770.600 |
| 285 | -1.300 | 0.041 | -1.016 | 0.007 | -0.952 | 1982.030 |
| 287 | -0.662 | 0.363 | -1708.350 | 0.066 | -0.598 | 0.775 |
| 293 | 0.000 | 0.000 | 0.000 | 1.000 | -0.939 | 8051.270 |
| 296 | 0.000 | 0.000 | 0.000 | 1.000 | -0.976 | 15770.600 |
| 297 | -1.294 | 0.034 | -1.072 | 0.006 | -0.954 | 1046.040 |
| 300 | -1.243 | 0.033 | -1.098 | 0.006 | -0.952 | 2463.830 |
| 313 | -0.598 | 0.190 | -0.739 | 0.047 | -0.950 | 2481.840 |
| 314 | -1.094 | 0.089 | -0.902 | 0.091 | -0.772 | 32.454 |
| 315 | -0.935 | 0.194 | -1703.840 | 0.206 | -0.533 | 0.469 |
| 316 | -1.243 | 0.033 | -1.098 | 0.006 | -0.952 | 2463.830 |
| 317 | -0.689 | 0.126 | -0.955 | 0.184 | -0.779 | 59.044 |
| 318 | -1.099 | 0.042 | -1.132 | 0.001 | -0.951 | 573.969 |
| 320 | -1.629 | 0.030 | -83.759 | 0.001 | -0.925 | 829.508 |
| 321 | -0.254 | 0.447 | -0.112 | 0.195 | -0.923 | 293.620 |
| 322 | -0.340 | 0.333 | -0.194 | 0.092 | -0.962 | 1981.550 |
| 326 | -0.430 | 0.374 | -1.058 | 0.099 | -0.721 | 13.794 |
| 329 | -1.021 | 0.037 | -321.834 | 0.001 | -0.970 | 5028.160 |
| 333 | -1.000 | 0.158 | -0.751 | 0.221 | -0.704 | 4.517 |
| 334 | -0.884 | 0.103 | -0.926 | 0.046 | -0.747 | 22.340 |
| 335 | -0.608 | 0.219 | -0.372 | 0.072 | -0.925 | 757.137 |
| 337 | -0.886 | 0.103 | -0.957 | 0.038 | -0.770 | 34.423 |
| 338 | -0.819 | 0.139 | -0.876 | 0.036 | -0.956 | 3099.350 |
| 339 | -0.628 | 0.192 | -0.988 | 0.030 | -0.954 | 1038.570 |
| 341 | -1.113 | 0.112 | -0.981 | 0.073 | -0.747 | 43.956 |
| 352 | -1.042 | 0.107 | -1.081 | 0.330 | -0.696 | 3.449 |
| 356 | -0.978 | 0.110 | -18.496 | 0.010 | -0.698 | 3.728 |
| 358 | -0.469 | 0.338 | -0.305 | 0.282 | -0.773 | 77.534 |
| 359 | -0.122 | 0.602 | -1.970 | 0.088 | -0.728 | 7.045 |
| 361 | -1.243 | 0.141 | -1.794 | 0.337 | -0.676 | 2.096 |
| 363 | -0.870 | 0.205 | -1376.520 | 0.015 | -0.704 | 4.564 |
| 371 | -0.526 | 0.307 | -10.952 | 0.047 | -0.699 | 3.816 |
| 379 | -0.198 | 0.536 | -1.415 | 0.094 | -0.729 | 8.619 |
| 383 | -0.721 | 0.225 | -0.650 | 0.254 | -0.721 | 13.646 |
| 386 | 0.550 | 0.915 | -1706.570 | 0.006 | -0.606 | 0.826 |
| 389 | -0.417 | 0.360 | -1.207 | 0.016 | -0.895 | 96.836 |
| 390 | -1.109 | 0.096 | -18.916 | 0.007 | -0.764 | 39.101 |
| 396 | -0.681 | 0.111 | -0.670 | 0.008 | -0.970 | 4891.720 |
| 397 | -0.440 | 0.354 | -0.207 | 0.374 | -0.763 | 51.003 |
| 398 | -1.208 | 0.033 | -1.349 | 0.004 | -0.899 | 136.841 |
| 399 | -0.485 | 0.220 | -460.615 | 0.005 | -0.912 | 171.940 |
| 401 | -0.467 | 0.286 | -0.656 | 0.087 | -0.746 | 19.235 |
| 404 | -0.779 | 0.146 | -0.569 | 0.045 | -0.963 | 1570.280 |
| 409 | -0.257 | 0.481 | -1.187 | 0.073 | -0.741 | 18.911 |
| 415 | -0.531 | 0.311 | -0.350 | 0.320 | -0.767 | 126.018 |
| 418 | -0.801 | 0.194 | -1.035 | 0.118 | -0.722 | 15.099 |
| 423 | -1.185 | 0.148 | -2.909 | 0.161 | -0.679 | 2.218 |
| 424 | -0.396 | 0.375 | -1647.120 | 0.020 | -0.713 | 6.948 |
| 425 | -1.078 | 0.113 | -0.997 | 0.077 | -0.747 | 50.925 |
| 428 | -1.025 | 0.046 | -1.656 | 0.006 | -0.950 | 461.082 |
| 429 | -1.361 | 0.016 | -2.026 | 0.000 | -0.952 | 414.630 |
| 430 | -1.021 | 0.037 | -1.008 | 0.002 | -0.963 | 2061.530 |
| 432 | -1.084 | 0.150 | -1701.030 | 0.015 | -0.726 | 34.678 |
| 436 | -0.340 | 0.333 | -0.158 | 0.105 | -0.963 | 1937.980 |
| 439 | -0.669 | 0.115 | -0.499 | 0.020 | -0.935 | 468.473 |
| 440 | -0.779 | 0.146 | -0.569 | 0.045 | -0.963 | 1514.610 |
| 441 | -1.301 | 0.034 | -0.893 | 0.018 | -0.954 | 1039.210 |
| 443 | -0.608 | 0.186 | -0.372 | 0.061 | -0.950 | 2497.860 |
| 450 | -0.399 | 0.308 | -1162.360 | 0.045 | -0.722 | 15.435 |
| 452 | -0.925 | 0.034 | -181.008 | 0.003 | -0.974 | 2639.920 |
| 455 | -0.757 | 0.115 | -972.055 | 0.036 | -0.762 | 78.447 |
| 457 | -0.506 | 0.157 | -0.671 | 0.070 | -0.753 | 21.890 |
| 459 | -0.724 | 0.172 | -1164.340 | 0.010 | -0.729 | 54.518 |
| 460 | -0.577 | 0.197 | -0.661 | 0.488 | -0.695 | 3.414 |
| 461 | -1.068 | 0.083 | -6.170 | 0.059 | -0.702 | 4.395 |
| 464 | -0.162 | 0.505 | -0.137 | 0.538 | -0.754 | 78.749 |
| 474 | -0.315 | 0.396 | -1.874 | 0.066 | -0.713 | 6.763 |
| 475 | -0.548 | 0.147 | -0.751 | 0.040 | -0.975 | 9087.860 |
| 476 | -0.484 | 0.111 | -0.494 | 0.006 | -0.983 | 12024.400 |
| 477 | -0.823 | 0.157 | -2.525 | 0.137 | -0.691 | 3.035 |
| 478 | -0.778 | 0.089 | -0.511 | 0.142 | -0.808 | 56.767 |
| 479 | -0.454 | 0.296 | -1.183 | 0.042 | -0.779 | 58.196 |
| 480 | -0.467 | 0.299 | -1.134 | 0.057 | -0.786 | 71.832 |
| 481 | -0.876 | 0.034 | -0.552 | 0.030 | -0.974 | 8728.120 |
| 489 | -0.270 | 0.437 | -0.486 | 0.270 | -0.731 | 51.018 |
| 491 | -0.482 | 0.200 | -0.552 | 0.038 | -0.954 | 2567.790 |
| 502 | -0.948 | 0.037 | -0.842 | 0.028 | -0.781 | 27.936 |
| 503 | -0.849 | 0.030 | -0.708 | 0.006 | -0.932 | 275.978 |
| 504 | -0.463 | 0.183 | -0.235 | 0.063 | -0.974 | 3097.510 |
| 513 | -0.446 | 0.190 | -0.566 | 0.030 | -0.974 | 2914.960 |
| 514 | -0.532 | 0.215 | -0.895 | 0.173 | -0.683 | 2.481 |
| 517 | -0.767 | 0.224 | -1.820 | 0.220 | -0.672 | 1.939 |
| 523 | -3.410 | 0.185 | -1.237 | 0.052 | -0.855 | 51.327 |
| 524 | -3.832 | 0.156 | -0.737 | 0.055 | -0.874 | 121.944 |
| 525 | -6.453 | 0.034 | -11.661 | 0.004 | -0.854 | 46.495 |
| 526 | 0.000 | 0.000 | 0.000 | 1.000 | -0.882 | 186.763 |
| 527 | -5.381 | 0.037 | -278.048 | 0.001 | -0.872 | 125.801 |
| 528 | -1.140 | 0.205 | -0.588 | 0.038 | -0.948 | 2174.770 |
| 529 | -2.173 | 0.136 | -99.784 | 0.022 | -0.713 | 7.238 |
| 531 | -2.505 | 0.147 | -28.690 | 0.051 | -0.834 | 5.821 |
| 532 | -1.430 | 0.125 | -1.026 | 0.037 | -0.749 | 34.024 |
| 536 | 0.000 | 0.000 | 0.000 | 1.000 | -0.933 | 6488.700 |
| 540 | -0.479 | 0.438 | -0.153 | 0.173 | -0.900 | 180.976 |
| 541 | -1.211 | 0.112 | -0.594 | 0.017 | -0.952 | 1668.210 |
| 553 | -1.537 | 0.147 | -1.084 | 0.142 | -0.700 | 3.881 |
| 554 | -1.153 | 0.271 | -0.704 | 0.161 | -0.773 | 88.702 |
| 557 | -0.361 | 0.523 | -2333.930 | 0.031 | -0.689 | 2.797 |
| 558 | -1.272 | 0.175 | -1.170 | 0.005 | -0.901 | 116.943 |
| 559 | -2.267 | 0.032 | -1.184 | 0.004 | -0.965 | 3433.140 |
| 560 | -1.211 | 0.111 | -0.686 | 0.011 | -0.959 | 2159.110 |
| 562 | -1.918 | 0.113 | -88.546 | 0.009 | -0.910 | 450.663 |
| 563 | -1.374 | 0.165 | -0.740 | 0.041 | -0.918 | 433.668 |
| 564 | -0.648 | 0.351 | -0.194 | 0.152 | -0.890 | 194.821 |
| 565 | -1.364 | 0.111 | -0.690 | 0.015 | -0.920 | 676.996 |
| 566 | -0.991 | 0.217 | -0.438 | 0.060 | -0.866 | 65.632 |
| 567 | -2.047 | 0.037 | -1.335 | 0.003 | -0.920 | 662.899 |
| 568 | -1.362 | 0.211 | -1.004 | 0.158 | -0.696 | 3.420 |
| 570 | -1.586 | 0.143 | -0.540 | 0.050 | -0.923 | 623.757 |
| 572 | -0.749 | 0.467 | -3308.630 | 0.044 | -0.548 | 0.522 |
| 573 | -0.519 | 0.491 | -3305.320 | 0.038 | -0.655 | 1.473 |
| 574 | -0.656 | 0.420 | -0.155 | 0.510 | -0.745 | 62.982 |
| 575 | -1.132 | 0.357 | -3303.880 | 0.079 | -0.718 | 10.071 |
| 577 | -0.763 | 0.384 | -3296.520 | 0.056 | -0.699 | 3.802 |
| 578 | -1.385 | 0.164 | -0.710 | 0.046 | -0.908 | 570.296 |
| 580 | -1.309 | 0.183 | -0.788 | 0.040 | -0.910 | 324.344 |
| 581 | -1.285 | 0.188 | -0.712 | 0.045 | -0.909 | 321.123 |
| 582 | -1.309 | 0.257 | -2.403 | 0.027 | -0.730 | 10.008 |
| 597 | -1.849 | 0.239 | -1.826 | 0.297 | -0.614 | 0.898 |
| 599 | -1.023 | 0.259 | -5.033 | 0.055 | -0.739 | 23.893 |
| 600 | -2.749 | 0.072 | -1.627 | 0.066 | -0.733 | 18.113 |
| 603 | -1.586 | 0.143 | -0.540 | 0.044 | -0.942 | 2956.710 |
| 604 | -0.774 | 0.317 | -2.266 | 0.057 | -0.726 | 18.643 |
| 605 | -0.916 | 0.038 | -1.932 | 0.002 | -0.932 | 247.950 |
| 607 | -0.684 | 0.130 | -1.314 | 0.002 | -0.949 | 341.896 |
| 608 | -0.345 | 0.375 | -0.173 | 0.352 | -0.795 | 94.050 |
| 611 | -0.667 | 0.091 | -2.027 | 0.003 | -0.937 | 265.478 |
| 617 | 0.295 | 0.152 | -0.078 | 0.957 | -0.730 | 167.356 |
| 619 | -0.428 | 0.341 | -0.576 | 0.160 | -0.794 | 102.793 |
| 622 | -0.438 | 0.224 | -0.655 | 0.037 | -0.968 | 1779.740 |
| 625 | -0.433 | 0.254 | -3.827 | 0.000 | -0.975 | 834.085 |
| 626 | -0.420 | 0.259 | -0.388 | 0.077 | -0.834 | 93.525 |
| 629 | -0.956 | 0.033 | -3.019 | 0.001 | -0.939 | 231.114 |
| 630 | -0.700 | 0.111 | -0.683 | 0.030 | -0.834 | 39.352 |
| 633 | -0.840 | 0.037 | -0.929 | 0.001 | -0.980 | 2601.170 |
| 637 | 0.285 | 0.084 | 0.119 | 0.784 | -0.730 | 215.726 |
| 640 | -1.120 | 0.012 | -173.597 | 0.002 | -0.984 | 6332.680 |
| 643 | -0.356 | 0.344 | -2.839 | 0.045 | -0.711 | 6.235 |
| 646 | -0.530 | 0.185 | -0.537 | 0.033 | -0.975 | 1732.370 |
| 649 | -1.099 | 0.096 | -1401.630 | 0.025 | -0.725 | 5.052 |
| 651 | -0.654 | 0.217 | -0.493 | 0.064 | -0.913 | 157.562 |
| 656 | 0.000 | 0.000 | 0.000 | 1.000 | -0.938 | 59.492 |
| 661 | -0.993 | 0.163 | -12.216 | 0.045 | -0.684 | 2.459 |
| 680 | -0.711 | 0.073 | -0.672 | 0.007 | -0.939 | 241.834 |
| 682 | -0.549 | 0.277 | -11.065 | 0.041 | -0.719 | 11.503 |
| 705 | -0.545 | 0.218 | -0.567 | 0.149 | -0.720 | 10.498 |
| 711 | -0.677 | 0.164 | -1.163 | 0.177 | -0.683 | 2.433 |
| 718 | -0.208 | 0.449 | -0.103 | 0.173 | -0.954 | 414.733 |
| 723 | -0.543 | 0.259 | -0.805 | 0.068 | -0.752 | 11.256 |
| 738 | -0.960 | 0.055 | -0.916 | 0.032 | -0.784 | 40.395 |
| 741 | -0.721 | 0.188 | -0.657 | 0.129 | -0.726 | 21.642 |
| 746 | -0.384 | 0.339 | -0.264 | 0.269 | -0.818 | 74.537 |
| 747 | -0.544 | 0.290 | -1.555 | 0.061 | -0.771 | 56.931 |
| 749 | -0.499 | 0.187 | -0.318 | 0.052 | -0.978 | 1933.520 |
| 750 | -0.510 | 0.205 | -0.457 | 0.116 | -0.767 | 28.253 |
| 762 | -0.592 | 0.201 | -0.450 | 0.056 | -0.885 | 33.676 |
| 763 | -0.865 | 0.148 | -0.579 | 0.533 | -0.676 | 2.102 |
| 766 | -0.581 | 0.166 | -1.222 | 0.048 | -0.750 | 12.908 |
| 767 | -1.172 | 0.059 | -21.097 | 0.009 | -0.720 | 9.712 |
| 775 | -0.391 | 0.337 | -0.703 | 0.119 | -0.821 | 65.094 |
| 786 | 0.000 | 0.000 | 0.000 | 1.000 | -0.977 | 1939.040 |
| 791 | -0.531 | 0.333 | -0.802 | 0.048 | -0.860 | 14.453 |
| 794 | -0.667 | 0.377 | -13.474 | 0.095 | -0.682 | 2.126 |
| 796 | -0.565 | 0.258 | -0.716 | 0.091 | -0.743 | 7.604 |
| 804 | -2.787 | 0.182 | -0.453 | 0.056 | -0.938 | 94.892 |
| 805 | -2.657 | 0.203 | -0.849 | 0.136 | -0.722 | 5.187 |
| 820 | -3.943 | 0.077 | -5.267 | 0.009 | -0.880 | 29.974 |
| 823 | -2.672 | 0.209 | -142.953 | 0.006 | -0.938 | 159.758 |
| 835 | -4.657 | 0.089 | -0.645 | 0.156 | -0.745 | 8.628 |

**Multiple sequence alignment for sequences from group 2.** Positions predicted to be under positive selection by at least two methods or under negative selection by all 3 methods are indicated in the first two rows.

10 20 30 40 50 60 70 80 90 100

....|....|....|....|....|....|....|....|....|....|....|....|....|....|....|....|....|....|....|....|

**Positive_selection/1** **----------------------------------------------------------------------------------------------------** 1

**Negative_selection/1** **--------------------------------------------N---------------NN--N---N---------------------------NN--** 7

**GmrSD|gi|108562912|H** **------------------------------------------MKAGEATLLEFFEQNQTNQFVIPIYQRLYSWGKEQCKQLWDDIIKIGGNDKMNGHFIG** 58

**GmrSD|gi|15611529|H_** **------------------------------------------MKAEATTLLGFFEENQNNQFVIPIYQRVYSWEKEQCEQLWDDIIKIGGNDKINGHFIG** 58

**GmrSD|gi|218703037|E** **MGYAGRMWCNGESPSRNDGIYMSPRQIRGYYCEIIASRKDVRMKATEARLLDFLKR--SQQFVIPIYQRTYSWTGQQCRQLWDDIIRAGKRDDISAHFIG** 98

**GmrSD|gi|298675128|M** **------------------------------------------MIAKETNLHTFLSG--PQQFIVPIYQRTYSWDLKHCKKLWDDVERLLSGQYVN-HFLG** 55

**GmrSD|gi|325283700|D** **------------------------------------------MKAEQTTLHRMMSGP-NQQFEMPLYQRRYSWTDKHRWQLWRDVLRASELRGESKHFTG** 57

110 120 130 140 150 160 170 180 190 200

....|....|....|....|....|....|....|....|....|....|....|....|....|....|....|....|....|....|....|....|

**Positive_selection/1** **----------------------------------------------------------------------------------------------------** 1

**Negative_selection/1** **N-------------------NN-N-N-NN--NN--NN---N--------------------NN-N-----------N-----N-----------------** 24

**GmrSD|gi|108562912|H** **SILYVR---VDDTHSSPLLIIDGQQRLTTITLLFIALRDHLNDEDE--FLEKFSCQKIQNRYLINSDEK-GDKKFKLFLSEPDRDTLLSLIDKDRRKPSE** 152

**GmrSD|gi|15611529|H_** **SILYVLDG---NTPSSPLLIIDGQQRLTTITLLCIALRSHLSDEVK--------REEIES-YLINSDKD-GDKKFRLILSESDKDTLLSLIDKNKRKPSE** 145

**GmrSD|gi|218703037|E** **SVVYIEQGLYQVSGISPLLVIDGQQRLTTAMLLIEALSRHLGEEEV---FDGFSAMKLRNYYLLNPYES-GEKGYKLLLTETDKDSLLALIKQRPMPEN-** 193

**GmrSD|gi|298675128|M** **SFVYINSDIYQVSSVPQLHIIDGQQRLTTISLLLQALIDVLQNQDV--KVKGVSSNKIRNYYLINTLEEEEELRYKLVLNRNDSETYTNLLENRELNDN-** 152

**GmrSD|gi|325283700|D** **SVVSAPVM-TIPGSVQHYRLVDGQQRMTTVHLLIWALAEHLENNPRAGDELGLKARQLRDQYLFNEGET-RDDRYKLRLNYADNPLWQRIMDGKFQGKAA** 155

210 220 230 240 250 260 270 280 290 300

....|....|....|....|....|....|....|....|....|....|....|....|....|....|....|....|....|....|....|....|

**Positive_selection/1** **----------------------------------------------------------------------------------------------------** 1

**Negative_selection/1** **--------------N-----------N---------N--N---N----N-----NNN-------N-N-NN-NN-----------N-----------N--N** 42

**GmrSD|gi|108562912|H** **---PSLKIMENFKLFEEWIRKN-TDKLETIFKGLEKLMIVWIALKKEKDDPQLIFESMNSKGIELTQADLIRNYIIMETEV-EKQEDFYNQYWRAMEEDF** 247

**GmrSD|gi|15611529|H_** **---PSVKIMENFKLFEKWISKN-TDKLETIFKGLEKLMIVYIALDKEKDDPQLIFESMNSKGIELTQTDLIRNYIIMETETEEKQKDFYNGYWRAMEEDF** 241

**GmrSD|gi|218703037|E** **---YSHRIMENFTFFDEQIAKL-GDDLIPLCRGLAKLLIVDVALNRGQDNPQLIFESMNSTGKALSQADLVRNFILMGLEP-EHQTRLYEDHWRPMEVAF** 288

**GmrSD|gi|298675128|M** **---YSPNVYKNYKFFKDKIEGYEPEYLSEILKTLQKLMIVDISLNPNEDNPQLIFESLNSTGLDLSQSDLIRNYLLMGQDH-QSQKELYNNYWFKMEQSF** 248

**GmrSD|gi|325283700|D** **RGERETELMRGVRFFRDRFAEP-GLDLAAVWEGFRRLEVVGILLQEGVDDMGVVFESLNSTGKALAQADLIRNNVLMSKSL-DDQRDITEQHWHPMEQRF** 253

310 320 330 340 350 360 370 380 390 400

....|....|....|....|....|....|....|....|....|....|....|....|....|....|....|....|....|....|....|....|

**Positive_selection/1** **----------------------------------------------------------------------------------------------------** 1

**Negative_selection/1** **------------N--N-N-N--------N--------NN-N------------------------------------------------------N-N--** 52

**GmrSD|gi|108562912|H** **KQSKKQSKKQDLFDKFVRHYLTIKTGKIPNEKRVYEAFKDYRQKKG---IEIEDLLKDLQKYCGYFCQIAFKKEADKDLNKALGFLVDLEMDVIYPLLLE** 344

**GmrSD|gi|15611529|H_** **------KQNETLFNRFVRHYLTIKTGKIPNIKKVYGAFKDYQQKEG---IEIEDLLKDLQKYCGYFCQIAFKKEANKDLNKALSFLVDLEMDVVYPLLLE** 332

**GmrSD|gi|218703037|E** **----GQQGYSEYFDSFMRHYLTVKTGEIPRTDEVYEAFKLHARSPSVAEKGVDRLVEDIHIYAEYYCAMALGKESDKSLATAFQDLRELKVDVAYPFLLA** 384

**GmrSD|gi|298675128|M** **----GHEKYQKRFDNFMRDYLTIKLSRIPVFDEIYEEFKQYYKKSG---ITTEDVINDIYKYSKLYVKIAFNMEKDEDLNNKFNEITTLKVDVAYPFLLQ** 341

**GmrSD|gi|325283700|D** **-----AEVEEGQFDRFMRDYLTLRTRILPNEDEVYLAFKQYREGKN-----VLSVVRNIDETSKLYLELLQPTHSAPAVQVALRDIAALKLSIVNPFLLE** 343

410 420 430 440 450 460 470 480 490 500

....|....|....|....|....|....|....|....|....|....|....|....|....|....|....|....|....|....|....|....|

**Positive_selection/1** **----------------------------------------------------------------------------------------------------** 1

**Negative_selection/1** **---N--------------------N--NNN--------NNN-N--------N----------------------NN----N---------N---------** 66

**GmrSD|gi|108562912|H** **LYSDYSDGVLSKQDFIPIIYLTESYICRRAVCGLGTNSLNKVFPFVTKKINKDQY-LESIKVHFGYL--TEKQRFPNNDEFKELFITIDFYHFQKREYFF** 441

**GmrSD|gi|15611529|H_** **LYSDYSDGVLSDQDFTTISTLTESYLCRRAVCGLGTNSLNKIFPFFTKKIDKKQY-LKSVEKHFGSL--KGKQRFPNNDEFKDSFITIDFYKFKKNKYFL** 429

**GmrSD|gi|218703037|E** **LYHDYKNGVLSQEDFLNIIRLIESYVFRRAVCAIQTNSLNKTFATFYKVINKEKY-LESIQVHFLNL--PSYRRFPNDDEFKRELKVRDLYNFRSRSYWL** 481

**GmrSD|gi|298675128|M** **VYDDYQENVIEKNDFIKILDTVISYVFRRAICGIPTNSLNKTFSSLMDEVDKNKY-VESVKAALSLK--KSYRKFPSDDEFYYKLISIDVYNLRNCKYLL** 438

**GmrSD|gi|325283700|D** **LLEDREQNLLTDEQLEKALRVVESFLVRRAVLKERTSPLNKLFATLGRELDKDSDYVRSLERALVRFQDNKTDGFPDDVAFEQALREQKLYGRPVCKPLL** 443

510 520 530 540 550 560 570 580 590 600

....|....|....|....|....|....|....|....|....|....|....|....|....|....|....|....|....|....|....|....|

**Positive_selection/1** **---------------------------------P------------------------------------------------------------------** 1

**Negative_selection/1** **--NN--------N---------NNN-NN------------N----------------NNN-NN-N-N--N-------N-NN-------------------** 86

**GmrSD|gi|108562912|H** **ERLENFER---KERVYTHEYTTEHIMPQT--LEEEWQRDLGE------NFEAIHEKYLHTIGNLTLTGYNSEYSNNSFQEKRDM------------EKGF** 518

**GmrSD|gi|15611529|H_** **ERLEKFDT---NEPVDTQKCEIEHIMPQT--LNPKWEGDLGE------NFEAIHEKYLHTIGNLTLTGYNAKYSNKSFQEKRDM------------EKGF** 506

**GmrSD|gi|218703037|E** **RRLENNKR---RERVD-DEFTIEHIMPQNENLSVKWREELGS------DWQRVHKELLHTLGNLTLTRYNSRYSDRSFAEKRDI------------EDGF** 559

**GmrSD|gi|298675128|M** **SKLENSHKWKIKEPINVDRYSIEHIMPQNPNLPDSWKKRLGR------DYKQVQNQYLHTLGNLTLTVHNSELGDMDFIEKKE---------------AY** 517

**GmrSD|gi|325283700|D** **VRLERGLNP--KETFG-GVLTIEHIMPQK--PGPSWQAALGKTPDDDKTWREDHERLLHTLGNLTLTGYNSELGNLSFEEKKHKPIRAGDSEETSIPKGY** 538

610 620 630 640 650 660 670 680 690 700

....|....|....|....|....|....|....|....|....|....|....|....|....|....|....|....|....|....|....|....|

**Positive_selection/1** **----------------------------------------------------------------------------------------------------** 1

**Negative_selection/1** **--N-N-N---N-------N--N------N---N------N-----N---------------------------------N--------------------** 97

**GmrSD|gi|108562912|H** **KQSPLRLNQGLRDLESFGEEEIEKRANDLADLALKIWTYPK-LDAETLEKYKP---KKEKK---------------AYDL-SS-----YKFGSH------** 587

**GmrSD|gi|15611529|H_** **KQSSLKLNKNLKDLEPFGEKEIEKRASDLVDWALKIWTYPI-LEAETLEEYKPKKEKKEKKEKKEKEEYKLKKEKKVYDL-SS-----YKFSPH------** 593

**GmrSD|gi|218703037|E** **KHSPLYLNIGLGQCEKWDEAAIRARADRLADLAVQVWQAPS-LPEEVLAVYRGKPE-NKTS----------------YSL-SD-----YPFLADG-----** 630

**GmrSD|gi|298675128|M** **DKSPLYLNDSLKGYDTWNEETIVERTEELAKKALEVWAYPY-LAPETLEFYKQQFENTASS---------------SKDL-NL-----PEVVSE------** 589

**GmrSD|gi|325283700|D** **KYSHLLLTRELVDKSEWNQGEIEARAKRLAKRATELFQMPSFT-PEEVE-----------------------------ALRAEGRQRARANTVERHLEEA** 608

710 720 730 740 750 760 770 780 790 800

....|....|....|....|....|....|....|....|....|....|....|....|....|....|....|....|....|....|....|....|

**Positive_selection/1** **---------------------------------P------------------------------------------------------------------** 2

**Negative_selection/1** **------------------------------------------------N---------------------------------------------------** 98

**GmrSD|gi|108562912|H** **---SRELFDILRKEIKALDERITENF-------VKTYIAYKFKT-NFVDIVVQTKD-----LKLYLKMELNELQDEIKE-KLKIRDVSNIGRPCVGN---** 667

**GmrSD|gi|15611529|H_** **---SRELFDILRKEIKTLDERVTENF-------NSKYIAYKFFKINFVDIVVQNKV-----LKLYLKMEFNELQDEIKE-KLKIRDVSNIGCPCSED---** 674

**GmrSD|gi|218703037|E** **-SHSRELFDHLRDEVMRLDAGITQEV-------LKLYIAFKAET-NFVDVVPQKSR-----LRLSLNMQFHELVDP----KGIAKDVTNVGRWGNGD---** 709

**GmrSD|gi|298675128|M** **---KAKNSEMEPVNVKGLKTRYPTVYPEDLEIGDKLILDYGETA-DIVDIELKGQK------TYAITFELDKTGE---------IDMIN-----------** 659

**GmrSD|gi|325283700|D** **SLQLRQLFEELDCHLKALGDE---EGVTVKRVINQQYIAYKAGS-NFCDVKVM---ASQNVLKCWLNIPVIQLDD----PLGLVRDMSQ-----TGHASN** 692

810 820 830 840 850 860 870 880 890 900

....|....|....|....|....|....|....|....|....|....|....|....|....|....|....|....|....|....|....|....|

**Positive_selection/1 ---------------------------------------------------------------------------------------------------- 2**

**Negative_selection/1 ---------------------------------------------------------------------------------------------------- 98**

**GmrSD|gi|108562912|H** **--MEVEL-ETKENIPYCLGLIKQILEK----QMGGRNG--------------------------------------------------------------** 698

**GmrSD|gi|15611529|H_** **--MEVEL-ETKENIPYCLGLIRQALEK----QMGGRNRQ-------------------------------------------------------------** 706

**GmrSD|gi|218703037|E** **--VEIGF-SDLAQLPYIMGLIRQAFEK----QMESALV--------------------------------------------------------------** 740

**GmrSD|gi|298675128|M** **--------KLETTLVTIL----------------------------------------------------------------------------------** 669

**GmrSD|gi|325283700|D** **APAEVTLT-LDSDMESFLELAEQALQHQVSLRATSSASGDSESGETVNFSVLPAEAQDVLARLSKWAADVGAKVKDTQNYRALRDRRSFVELYPRQRENG** 791

910 920 930 940 950 960 970 980 990 1000

....|....|....|....|....|....|....|....|....|....|....|....|....|....|....|....|....|....|....|....|

**Positive_selection/1 ---------------------------------------------------------------------------------------------------- 2**

**Negative_selection/1 ---------------------------------------------------------------------------------------------------- 98**

**GmrSD|gi|108562912|H** **----------------------------------------------------------------------------------------------------** 698

**GmrSD|gi|15611529|H_** **----------------------------------------------------------------------------------------------------** 706

**GmrSD|gi|218703037|E** **----------------------------------------------------------------------------------------------------** 740

**GmrSD|gi|298675128|M** **----------------------------------------------------------------------------------------------------** 669

**GmrSD|gi|325283700|D** **LILALKLPADEVGELTEGQLGGWLRRDSWLSKVIRTPEELEKAWPLIEAAHAYQKPSGVLSSAQGIELRDFYQKVLQLAEEEFTPQALARQNKQTLLRLP** 891

1010 1020 1030 1040 1050 1060 1070 1080

....|....|....|....|....|....|....|....|....|....|....|....|....|....|....|....|....|

**Positive_selection/1 ------------------------------------------------------------------------------------- 2**

**Negative_selection/1 ------------------------------------------------------------------------------------- 98**

**GmrSD|gi|108562912|H** **-------------------------------------------------------------------------------------** 698

**GmrSD|gi|15611529|H_** **-------------------------------------------------------------------------------------** 706

**GmrSD|gi|218703037|E** **-------------------------------------------------------------------------------------** 740

**GmrSD|gi|298675128|M** **-------------------------------------------------------------------------------------** 669

**GmrSD|gi|325283700|D** **ESEEYIARINMVKQKGSVEVLMDIPFEDVVDFTGRGFARMEHSSLSRGHYSTFLKPGEEDMARQLLRHLNRHFQAQAAATPGEQS** 976

**Table 5.**

**Positively selected sites reported by at least one method for sequence group 3.**

| **Codon** | **SLAC dN-dS** | **SLAC p-value** | **FEL dN-dS** | **FEL p-value** | **REL dN-dS** | **REL Bayes Factor** | **MEME ω^+^** | **MEME p-value** |
| --- | --- | --- | --- | --- | --- | --- | --- | --- |
| 212 | 0.464 | 0.377 | 0.214 | 0.062 | -0.826 | 1.000 | 23.198 | 0.670 |
| 284 | -0.010 | 0.694 | 0.016 | 0.902 | -0.799 | 1.000 | >100 | 0.050 |
| 288 | 0.529 | 0.165 | 0.329 | 0.189 | -0.867 | 1.000 | 14.890 | 0.670 |
| 435 | 0.296 | 0.481 | 0.178 | 0.308 | -0.819 | 1.000 | >100 | 0.081 |
| 484 | 0.330 | 0.293 | 0.236 | 0.049 | -0.699 | 1.000 | >100 | 0.080 |
| 494 | 0.535 | 0.197 | 0.861 | 0.722 | -1.211 | 1.000 | 19.176 | 0.670 |
| 497 | 0.597 | 0.140 | 0.293 | 0.082 | -0.811 | 1.000 | 11.880 | 0.670 |
| 507 | 0.516 | 0.080 | 0.177 | 0.021 | -0.787 | 1.000 | 6.799 | 0.663 |
| 725 | 0.375 | 0.219 | 0.081 | 0.749 | -0.722 | 1.000 | 2.664 | 0.622 |
| 729 | 0.104 | 0.572 | -0.001 | 0.996 | -0.851 | 1.000 | >100 | 0.100 |
| 734 | -0.139 | 0.784 | 0.019 | 0.922 | -0.725 | 1.000 | >100 | 0.037 |
| 745 | 0.361 | 0.212 | 0.118 | 0.174 | -0.347 | 1.000 | 6.409 | 0.281 |
| 832 | 0.525 | 0.149 | 0.133 | 0.098 | -0.705 | 1.000 | 0.144 | 0.670 |
| 897 | 0.461 | 0.193 | 0.111 | 0.194 | -0.680 | 1.000 | >100 | 0.136 |
| 970 | 0.460 | 0.088 | 0.299 | 0.024 | -0.573 | 1.000 | >100 | 0.493 |
| 978 | 0.405 | 0.191 | 0.227 | 0.062 | -0.893 | 1.000 | 0.016 | 0.670 |
| 1007 | 0.514 | 0.217 | 0.401 | 0.377 | -0.896 | 1.000 | >100 | 0.114 |
| 1056 | 0.485 | 0.225 | 0.257 | 0.285 | -0.866 | 1.000 | >100 | 0.489 |
| 1066 | 0.170 | 0.387 | -0.050 | 0.703 | -0.828 | 1.000 | 7.538 | 0.031 |
| 1107 | 0.322 | 0.415 | 0.133 | 0.046 | -0.656 | 1.000 | 1.665 | 0.670 |
| 1187 | 0.150 | 0.481 | 0.107 | 0.333 | -0.310 | 1.000 | 11.010 | 0.026 |
| 1200 | 0.082 | 0.518 | 0.373 | 0.084 | -0.820 | 1.000 | >100 | 0.128 |
| 1263 | 0.478 | 0.219 | 0.100 | 0.426 | -0.929 | 1.000 | 1.269 | 0.670 |

**Table 6.**

**Negatively selected sites reported by at least one method for sequence group 3.**

| **Codon** | **SLAC dN-dS** | **SLAC p-value** | **FEL dN-dS** | **FEL p-value** | **REL dN-dS** | **REL Bayes Factor** |
| --- | --- | --- | --- | --- | --- | --- |
| 4 | -1.809 | 0.038 | -1.289 | 0.124 | -0.967 | 0.000 |
| 16 | -0.330 | 0.212 | -0.392 | 0.150 | -0.983 | 0.000 |
| 21 | -0.494 | 0.131 | -724.778 | 0.040 | -1.156 | 0.000 |
| 28 | -0.445 | 0.248 | -0.111 | 0.745 | -0.841 | 0.000 |
| 30 | -0.682 | 0.053 | -0.367 | 0.027 | -0.999 | 0.000 |
| 31 | -0.428 | 0.148 | 2.041 | 1.000 | -1.069 | 0.000 |
| 32 | -0.259 | 0.213 | -725.204 | 0.021 | -1.135 | 0.000 |
| 33 | -0.629 | 0.001 | -0.892 | 0.000 | -1.183 | 0.000 |
| 34 | -0.362 | 0.164 | -720.960 | 0.040 | -1.326 | 0.000 |
| 35 | -1.095 | 0.001 | -2.158 | 0.000 | -1.318 | 0.000 |
| 36 | -1.085 | 0.000 | -23.581 | 0.000 | -1.089 | 0.000 |
| 37 | -0.517 | 0.007 | -0.155 | 0.000 | -0.864 | 0.000 |
| 38 | -0.364 | 0.196 | -0.973 | 0.382 | -1.155 | 0.000 |
| 40 | -0.472 | 0.073 | -0.497 | 0.027 | -1.064 | 0.000 |
| 43 | -0.500 | 0.128 | -0.277 | 0.098 | -0.915 | 0.000 |
| 45 | -0.533 | 0.123 | -0.146 | 0.218 | -0.938 | 0.000 |
| 46 | -0.612 | 0.017 | -0.341 | 0.003 | -1.023 | 0.000 |
| 49 | -0.391 | 0.075 | -0.896 | 0.000 | -1.352 | 0.000 |
| 50 | -0.600 | 0.011 | -0.383 | 0.001 | -1.159 | 0.000 |
| 52 | -0.543 | 0.017 | -0.151 | 0.002 | -0.894 | 0.000 |
| 53 | -0.654 | 0.017 | -0.110 | 0.036 | -0.494 | 0.000 |
| 57 | -0.924 | 0.040 | -1.732 | 0.046 | -1.126 | 0.000 |
| 69 | -0.327 | 0.219 | -0.099 | 0.454 | -0.829 | 0.000 |
| 71 | -1.178 | 0.002 | -0.382 | 0.002 | -1.058 | 0.000 |
| 73 | -0.489 | 0.048 | -0.326 | 0.010 | -0.985 | 0.000 |
| 74 | -0.524 | 0.005 | -0.150 | 0.001 | -0.985 | 0.000 |
| 76 | -0.536 | 0.086 | -724.259 | 0.035 | -1.196 | 0.000 |
| 77 | -0.420 | 0.103 | -0.305 | 0.036 | -1.044 | 0.000 |
| 95 | -0.942 | 0.014 | -0.887 | 0.003 | -1.238 | 0.000 |
| 98 | -0.436 | 0.082 | -724.648 | 0.009 | -1.104 | 0.000 |
| 99 | -0.275 | 0.127 | -0.077 | 0.023 | -0.614 | 0.000 |
| 100 | -0.734 | 0.000 | -6.315 | 0.000 | -1.128 | 0.000 |
| 101 | -0.641 | 0.006 | -0.139 | 0.001 | -0.904 | 0.000 |
| 102 | -0.708 | 0.005 | -0.382 | 0.000 | -1.059 | 0.000 |
| 103 | -0.681 | 0.002 | -0.609 | 0.000 | -1.189 | 0.000 |
| 105 | -0.318 | 0.099 | -0.058 | 0.088 | -0.758 | 0.000 |
| 107 | -0.637 | 0.002 | -0.933 | 0.000 | -1.278 | 0.000 |
| 109 | -0.610 | 0.029 | -0.186 | 0.005 | -0.328 | 0.000 |
| 114 | -0.315 | 0.180 | -725.187 | 0.010 | -1.249 | 0.000 |
| 131 | -1.103 | 0.227 | 66.694 | 1.000 | -0.919 | 0.000 |
| 179 | -0.454 | 0.143 | -725.224 | 0.002 | -1.409 | 0.000 |
| 181 | -0.453 | 0.176 | -724.653 | 0.019 | -1.248 | 0.000 |
| 184 | -0.609 | 0.005 | -10.205 | 0.000 | -1.282 | 0.000 |
| 185 | -0.433 | 0.183 | -0.211 | 0.549 | -0.897 | 0.000 |
| 191 | -0.630 | 0.016 | -0.182 | 0.005 | -0.310 | 0.000 |
| 206 | -0.546 | 0.241 | -0.796 | 0.316 | -0.959 | 0.000 |
| 214 | -0.756 | 0.172 | -0.290 | 0.307 | -0.927 | 0.000 |
| 238 | -0.746 | 0.128 | -15.982 | 0.033 | -0.993 | 0.000 |
| 263 | -0.362 | 0.194 | -10.894 | 0.102 | -1.028 | 0.000 |
| 267 | -0.176 | 0.371 | -725.145 | 0.081 | -1.106 | 0.000 |
| 282 | -0.609 | 0.236 | 0.157 | 0.722 | -0.880 | 0.000 |
| 283 | -0.920 | 0.068 | -721.411 | 0.403 | -1.002 | 0.000 |
| 290 | -0.318 | 0.239 | -725.106 | 0.566 | -0.936 | 0.000 |
| 298 | -0.898 | 0.050 | -0.375 | 0.060 | -1.020 | 0.000 |
| 299 | -0.187 | 0.337 | -723.013 | 0.054 | -1.133 | 0.000 |
| 303 | -0.479 | 0.041 | -0.291 | 0.010 | -1.020 | 0.000 |
| 304 | -0.482 | 0.191 | -725.141 | 0.192 | -1.168 | 0.000 |
| 305 | -0.401 | 0.189 | -0.229 | 0.165 | -0.799 | 0.000 |
| 306 | -0.428 | 0.113 | -0.489 | 0.113 | -1.096 | 0.000 |
| 307 | -0.351 | 0.203 | -725.037 | 0.180 | -0.989 | 0.000 |
| 309 | -0.349 | 0.232 | -725.214 | 0.081 | -1.043 | 0.000 |
| 316 | -0.445 | 0.066 | -0.204 | 0.020 | -0.903 | 0.000 |
| 320 | -0.785 | 0.007 | -725.209 | 0.000 | -1.240 | 0.000 |
| 321 | -0.539 | 0.055 | -0.258 | 0.012 | -0.801 | 0.000 |
| 322 | -1.000 | 0.010 | -0.187 | 0.025 | -0.835 | 0.000 |
| 323 | -0.317 | 0.207 | -0.266 | 0.054 | -0.736 | 0.000 |
| 325 | -0.230 | 0.243 | -0.057 | 0.160 | -0.541 | 0.000 |
| 326 | -0.768 | 0.034 | -725.208 | 0.003 | -1.315 | 0.000 |
| 327 | -0.469 | 0.130 | -724.471 | 0.314 | -1.228 | 0.000 |
| 328 | -0.718 | 0.145 | -707.466 | 0.038 | -1.227 | 0.000 |
| 329 | -0.270 | 0.129 | -0.061 | 0.029 | -0.607 | 0.000 |
| 331 | -0.647 | 0.044 | -725.150 | 0.000 | -1.510 | 0.000 |
| 332 | -0.524 | 0.004 | -0.144 | 0.000 | -0.972 | 0.000 |
| 333 | -0.278 | 0.293 | -0.841 | 0.048 | -1.100 | 0.000 |
| 334 | -0.543 | 0.088 | -0.381 | 0.032 | -0.999 | 0.000 |
| 335 | -0.723 | 0.009 | -5.315 | 0.000 | -1.510 | 0.000 |
| 338 | -0.243 | 0.323 | -724.992 | 0.026 | -1.219 | 0.000 |
| 340 | -0.245 | 0.203 | -0.039 | 0.331 | -0.464 | 0.000 |
| 341 | -0.270 | 0.222 | -0.075 | 0.215 | -0.491 | 0.000 |
| 342 | -0.475 | 0.028 | -0.207 | 0.002 | -0.957 | 0.000 |
| 343 | -0.314 | 0.102 | -0.083 | 0.045 | -0.810 | 0.000 |
| 344 | -0.559 | 0.109 | -1.859 | 0.038 | -1.264 | 0.000 |
| 345 | -0.277 | 0.201 | -725.216 | 0.000 | -1.462 | 0.000 |
| 346 | -0.646 | 0.075 | -0.523 | 0.018 | -1.131 | 0.000 |
| 349 | -0.635 | 0.053 | -725.145 | 0.028 | -1.202 | 0.000 |
| 351 | -0.662 | 0.071 | -720.868 | 0.082 | -1.046 | 0.000 |
| 359 | -0.365 | 0.252 | -724.524 | 0.098 | -1.126 | 0.000 |
| 360 | -0.324 | 0.236 | -1.068 | 0.192 | -0.969 | 0.000 |
| 364 | -0.488 | 0.038 | -0.315 | 0.010 | -1.022 | 0.000 |
| 366 | -0.406 | 0.136 | -0.508 | 0.039 | -1.043 | 0.000 |
| 369 | -0.301 | 0.194 | -2.447 | 0.015 | -1.132 | 0.000 |
| 370 | -0.353 | 0.221 | -0.167 | 0.080 | -0.813 | 0.000 |
| 385 | -0.268 | 0.306 | -725.097 | 0.067 | -1.149 | 0.000 |
| 389 | -0.262 | 0.353 | -724.174 | 0.044 | -1.204 | 0.000 |
| 403 | -1.452 | 0.108 | -724.275 | 0.551 | -0.975 | 0.000 |
| 422 | -0.770 | 0.051 | -0.322 | 0.045 | -0.974 | 0.000 |
| 496 | -0.796 | 0.008 | -0.394 | 0.004 | -1.104 | 0.000 |
| 500 | -0.372 | 0.212 | -0.117 | 0.466 | -0.814 | 0.000 |
| 693 | -0.448 | 0.214 | -0.466 | 0.200 | -0.956 | 0.000 |
| 710 | -0.395 | 0.081 | -0.096 | 0.094 | -0.685 | 0.000 |
| 711 | -0.442 | 0.044 | -0.106 | 0.042 | -0.706 | 0.000 |
| 713 | -0.819 | 0.002 | -0.714 | 0.000 | -1.118 | 0.000 |
| 714 | -0.405 | 0.088 | -0.170 | 0.013 | -0.916 | 0.000 |
| 716 | -0.375 | 0.149 | -0.151 | 0.079 | -0.924 | 0.000 |
| 717 | -0.504 | 0.025 | -0.203 | 0.000 | -0.520 | 0.000 |
| 718 | -0.392 | 0.206 | -0.062 | 0.387 | -0.181 | 0.000 |
| 719 | -0.463 | 0.084 | -0.173 | 0.031 | -0.913 | 0.000 |
| 722 | -0.553 | 0.082 | -0.371 | 0.019 | -1.042 | 0.000 |
| 723 | -0.154 | 0.365 | -725.146 | 0.037 | -1.129 | 0.000 |
| 751 | -0.641 | 0.095 | -725.171 | 0.334 | -1.022 | 0.000 |
| 763 | -0.642 | 0.128 | -152.804 | 0.034 | -1.012 | 0.000 |
| 765 | -0.247 | 0.333 | -45.889 | 0.072 | -0.973 | 0.000 |
| 768 | -0.185 | 0.237 | -674.347 | 0.543 | -0.943 | 0.000 |
| 774 | -0.370 | 0.230 | -1.622 | 0.061 | -1.117 | 0.000 |
| 777 | -0.472 | 0.066 | -0.982 | 0.015 | -1.109 | 0.000 |
| 780 | -0.818 | 0.002 | -57.718 | 0.000 | -1.216 | 0.000 |
| 784 | -0.443 | 0.072 | -725.015 | 0.000 | -1.433 | 0.000 |
| 785 | -0.273 | 0.234 | -0.106 | 0.157 | -0.121 | 0.000 |
| 786 | -0.641 | 0.122 | -0.189 | 0.391 | -0.759 | 0.000 |
| 788 | -0.432 | 0.062 | -0.428 | 0.000 | -1.013 | 0.000 |
| 789 | -0.384 | 0.170 | -0.080 | 0.483 | -0.614 | 0.000 |
| 790 | -0.320 | 0.125 | -0.076 | 0.117 | -0.726 | 0.000 |
| 791 | -0.466 | 0.084 | -0.098 | 0.150 | -0.696 | 0.000 |
| 792 | -0.819 | 0.002 | -0.714 | 0.000 | -1.118 | 0.000 |
| 796 | -0.583 | 0.034 | -0.319 | 0.015 | -1.006 | 0.000 |
| 813 | -0.174 | 0.401 | -725.239 | 0.079 | -1.018 | 0.000 |
| 830 | -0.473 | 0.117 | -3.161 | 0.002 | -1.311 | 0.000 |
| 842 | -0.337 | 0.268 | -0.549 | 0.074 | -1.067 | 0.000 |
| 848 | -0.549 | 0.122 | -0.171 | 0.455 | -0.867 | 0.000 |
| 851 | -0.435 | 0.193 | 0.339 | 0.964 | -0.955 | 0.000 |
| 868 | -0.610 | 0.173 | -20.453 | 0.046 | -0.986 | 0.000 |
| 869 | -3.721 | 0.074 | -0.103 | 0.942 | -0.883 | 0.000 |
| 876 | -1.086 | 0.227 | 0.248 | 0.867 | -0.918 | 0.000 |
| 882 | -1.610 | 0.018 | -725.169 | 0.020 | -1.221 | 0.000 |
| 884 | -0.833 | 0.015 | -1.409 | 0.001 | -1.239 | 0.000 |
| 886 | -0.728 | 0.266 | -725.046 | 0.035 | -1.242 | 0.000 |
| 888 | -0.399 | 0.247 | -0.323 | 0.176 | -0.874 | 0.000 |
| 889 | -0.420 | 0.093 | -0.529 | 0.017 | -1.145 | 0.000 |
| 890 | -0.219 | 0.247 | -0.048 | 0.225 | -0.767 | 0.000 |
| 916 | -0.534 | 0.196 | -0.029 | 0.924 | -0.811 | 0.000 |
| 928 | -0.524 | 0.230 | 1.443 | 0.930 | -0.937 | 0.000 |
| 929 | -0.689 | 0.166 | 30.787 | 1.000 | -1.048 | 0.000 |
| 962 | -0.506 | 0.170 | -0.302 | 0.167 | -0.934 | 0.000 |
| 966 | -0.655 | 0.122 | -725.190 | 0.099 | -1.058 | 0.000 |
| 969 | -0.336 | 0.248 | -0.268 | 0.416 | -0.876 | 0.000 |
| 996 | -0.196 | 0.410 | -0.400 | 0.099 | -0.922 | 0.000 |
| 997 | 0.000 | 0.000 | -3.548 | 0.047 | -0.998 | 0.000 |
| 1010 | -0.627 | 0.151 | -0.376 | 0.563 | -0.980 | 0.000 |
| 1043 | -0.503 | 0.102 | -0.376 | 0.664 | -0.928 | 0.000 |
| 1051 | -0.603 | 0.164 | 0.831 | 0.908 | -1.071 | 0.000 |
| 1062 | -0.363 | 0.126 | -725.116 | 0.010 | -1.198 | 0.000 |
| 1064 | -0.457 | 0.157 | -0.244 | 0.331 | -0.828 | 0.000 |
| 1067 | -0.594 | 0.102 | -0.764 | 0.006 | -1.214 | 0.000 |
| 1069 | 0.049 | 0.652 | -722.899 | 0.063 | -1.296 | 0.000 |
| 1070 | -0.543 | 0.017 | -0.160 | 0.002 | -0.862 | 0.000 |
| 1071 | -0.324 | 0.275 | -723.559 | 0.095 | -1.031 | 0.000 |
| 1073 | -0.319 | 0.152 | -0.115 | 0.051 | -0.728 | 0.000 |
| 1083 | -0.369 | 0.247 | -723.628 | 0.137 | -1.061 | 0.000 |
| 1099 | -0.890 | 0.087 | -0.018 | 0.981 | -0.902 | 0.000 |
| 1100 | -0.620 | 0.177 | 57.434 | 1.000 | -1.002 | 0.000 |
| 1122 | -4.733 | 0.071 | 10.377 | 1.000 | -0.926 | 0.000 |
| 1123 | -0.849 | 0.074 | 8.888 | 1.000 | -0.957 | 0.000 |
| 1126 | -2.222 | 0.138 | 0.145 | 0.990 | -0.917 | 0.000 |
| 1141 | -0.752 | 0.017 | -0.927 | 0.003 | -1.111 | 0.000 |
| 1147 | -0.578 | 0.081 | -2.133 | 0.015 | -1.159 | 0.000 |
| 1148 | -0.524 | 0.004 | -0.105 | 0.001 | -0.913 | 0.000 |
| 1149 | -0.297 | 0.085 | -0.051 | 0.065 | -0.756 | 0.000 |
| 1150 | -1.056 | 0.000 | -24.763 | 0.000 | -1.230 | 0.000 |
| 1151 | -0.270 | 0.129 | -0.060 | 0.029 | -0.630 | 0.000 |
| 1154 | -0.472 | 0.018 | -0.171 | 0.002 | -0.974 | 0.000 |
| 1155 | -0.980 | 0.001 | -0.237 | 0.003 | -0.847 | 0.000 |
| 1181 | -0.976 | 0.007 | -0.625 | 0.004 | -1.127 | 0.000 |
| 1183 | -0.825 | 0.007 | -0.310 | 0.002 | -0.938 | 0.000 |
| 1184 | -0.629 | 0.002 | -0.179 | 0.000 | -0.971 | 0.000 |
| 1185 | -0.818 | 0.002 | -113.172 | 0.000 | -1.187 | 0.000 |
| 1186 | -0.595 | 0.003 | -0.452 | 0.000 | -1.105 | 0.000 |
| 1188 | -0.561 | 0.070 | -723.723 | 0.001 | -1.453 | 0.000 |
| 1189 | 0.062 | 0.723 | -725.206 | 0.065 | -1.162 | 0.000 |
| 1190 | -0.310 | 0.237 | -725.121 | 0.197 | -1.103 | 0.000 |
| 1193 | -0.394 | 0.238 | 0.383 | 0.187 | -0.871 | 0.000 |
| 1194 | -0.819 | 0.002 | -0.204 | 0.001 | -0.897 | 0.000 |
| 1195 | -0.734 | 0.002 | -0.680 | 0.000 | -1.006 | 0.000 |
| 1198 | -0.925 | 0.040 | -725.182 | 0.016 | -1.233 | 0.000 |
| 1199 | -0.513 | 0.044 | -0.193 | 0.008 | -0.894 | 0.000 |
| 1205 | -0.432 | 0.131 | -42.621 | 0.012 | -1.057 | 0.000 |
| 1218 | -0.377 | 0.196 | -0.205 | 0.305 | -0.913 | 0.000 |
| 1227 | -0.352 | 0.110 | -0.062 | 0.248 | -0.624 | 0.000 |
| 1229 | -0.314 | 0.199 | -1.271 | 0.027 | -1.201 | 0.000 |
| 1230 | -0.308 | 0.248 | -0.414 | 0.179 | -0.933 | 0.000 |
| 1231 | -0.015 | 0.578 | -725.024 | 0.076 | -1.294 | 0.000 |
| 1243 | -0.477 | 0.149 | -725.137 | 0.033 | -1.072 | 0.000 |
| 1262 | -0.435 | 0.218 | -0.241 | 0.646 | -0.874 | 0.000 |
| 1269 | -0.184 | 0.390 | -723.704 | 0.094 | -1.100 | 0.000 |
| 1270 | -0.639 | 0.100 | -0.354 | 0.093 | -1.006 | 0.000 |
| 1272 | -0.498 | 0.047 | -0.230 | 0.011 | -1.012 | 0.000 |
| 1276 | -0.713 | 0.236 | -1.019 | 0.025 | -1.214 | 0.000 |
| 1277 | -0.025 | 0.573 | -3.261 | 0.047 | -1.183 | 0.000 |
| 1278 | -0.468 | 0.201 | -11.204 | 0.343 | -1.095 | 0.000 |
| 1280 | -0.531 | 0.076 | -0.627 | 0.014 | -1.185 | 0.000 |
| 1284 | -0.640 | 0.088 | -0.647 | 0.075 | -1.030 | 0.000 |

**Multiple sequence alignment for sequences from group 3.** Positions predicted to be under positive or negative selection by at least two methods are indicated in the first two rows.

10 20 30 40 50 60 70 80 90 100

....|....|....|....|....|....|....|....|....|....|....|....|....|....|....|....|....|....|....|....|

**Positive_selection/1** **----------------------------------------------------------------------------------------------------** 1

**Negative_selection/1** **--------------------N--------N--NNNNN--N--N--N--NN-NN---N-------------N-NN-NN-----------------N--NNN** 24

**GmrSD|gi|145633202|H** **----MSNAKEYVKTLTVSDLF-DNENKCNYIIPIYQRNYAWGDDEISSLLQDIKNACEKN----KEQNKNYYIGSLVVYCR---------ENGDFEVIDG** 82

**GmrSD|gi|147675278|V** **---MGDHLSVQTELLTLEKIY---TDNYQFSIPSYQRPYVWSDDDVLLLFRDIKEACRLKE-------PNYFIGTILSSRIEQ------DGERIYELIDG** 81

**GmrSD|gi|319955102|C** **-------MEVKYLNKSIREIF-----QSNYVVPLYQRNYAWADDEIHLLLRDIYENYSK------NPKGFYFIGTLVVLKR---------KNGDFEVIDG** 73

**GmrSD|gi|322691502|B** **MSKLVTELHIIGENG---NMFD---TDMEYIIPLYQRAYAWEDKQLTQLIEDINDVAE---------DSNYYIGSLIVSK----------QFGRYEVVDG** 75

**GmrSD|gi|347536664|F** **---------MEQNTNKFTPSTV---GNLEFSIPLYQRLFEWETSQINQLLNDLYENFEQN----EPENKPYYIGMFTVYNEKN--------NNQYSLVDG** 76

**GmrSD|gi|380567916|C** **---MENNSTNNQNQADVKNISN--INSIFFEIPPYQRLYEWNKEQIQTLLNDIKTKFEENK------NKEYFIGNVVVSKKNN---KNNQQDVKYLLIDG** 86

**GmrSD|gi|386866338|B** **------MMTGNPTILSIAELFN---EECVYRVPIYQRAYAWGTDEIKTLLDDLKRAAG----------TAYHLGSLVVAGR-----GDDQNRTVYEVVDG** 76

**GmrSD|gi|389857445|S** **--------MTKTATLTVTKLL----TEDEYIVPLYQRNFSWTYDEIEQLLIDIADAYNERE-----KRPDYYIGTLVVHQ----------KGNMYHIIDG** 73

**GmrSD|gi|411028915|G** **-------------------------------MPLYQRLYVWGDEQISTLLNDIANACERQD-------AQFFLGGILVVETTT-EEERRAKRRTFDLIDG** 61

**GmrS|gi|384207706|Gm** **-----------------------------MVIPEYQRFYVWDKENVDDLLNDLKDFFLNN------ENEEYYLGTVILHND--------KTNKKYNIIDG** 57

110 120 130 140 150 160 170 180 190 200

....|....|....|....|....|....|....|....|....|....|....|....|....|....|....|....|....|....|....|....|

**Positive_selection/1** **----------------------------------------------------------------------------------------------------** 1

**Negative_selection/1** **NNN-N-N-N----N----------------------------------------------------------------N-N--N------N---------** 35

**GmrSD|gi|145633202|H** **QQRLTTLTLIMHHLGKL-----------------------------------------GF-R---------------NVYFEHRDESQQAL---------** 116

**GmrSD|gi|147675278|V** **QQRTTTLMLMTIAFKYAGIKSDLAGLAVYTSSDGEDKP---------------------------------------RLQFSIREQVQQLL---------** 133

**GmrSD|gi|319955102|C** **QQRLTTLSLIAKKLDSS-----------------------------------------L---------------HTSKLQYDSRPEVESFL---------** 108

**GmrSD|gi|322691502|B** **QQRLTSLYLLLN---------------------------------------------------------CLGIKTKPTLTFACREKSNYTL---------** 109

**GmrSD|gi|347536664|F** **QQRFTVLTLMAIAFKTE----------------------------E--------WLHFLKINDNP------------RLSFFARKKDTDYL---------** 119

**GmrSD|gi|380567916|C** **QQRLTTLFLIGFYLSYK----------------------------IKNPNNDSNWKEFIMQGDKL------------RISMPIREKEEKAL---------** 137

**GmrSD|gi|386866338|B** **QQRLTTLYLLMAVLCHGD----------------------------------------D------DRAAGALTAWNMQLEYECRAESYGVLLERLRASGE** 130

**GmrSD|gi|389857445|S** **QQRTTALTLLALVLRNE-----------------------------------------Y----------HIQIPDLKLLNFEARKQSNASL---------** 113

**GmrSD|gi|411028915|G** **QQRFTTLFLLSQSPGWRKELSTFGRVERNGGFTS-------------------------------------------RLNFSVRPDVNRYF---------** 109

**GmrS|gi|384207706|Gm** **QQRILTLLIILYVFEQQ--------------------------------------KYNYVF-----------EQQKYNIEGKF-NN--------------** 93

210 220 230 240 250 260 270 280 290 300

....|....|....|....|....|....|....|....|....|....|....|....|....|....|....|....|....|....|....|....|

**Positive_selection/1** **----------------------------------------------------------------------------------------------------** 1

**Negative_selection/1** **-------------------------------------N-----------------------------------------------------------N--** 37

**GmrSD|gi|145633202|H** **---------------S-----------------------------NLNSGKLPSNFLQALKTIKKVI------------------DEWGNNKDEIVKFLL** 154

**GmrSD|gi|147675278|V** **----------------GGLAG--------------LKNYQVPSKETIADNAYLKQMGVALDVLTKEV--------------EKLKSDDGVSAEAMGDYLY** 189

**GmrSD|gi|319955102|C** **----------STYYQRGEV-----------------------------ATTTTNHLVSHFNEAIEYIETVDLKVKEPNETLRFIDFLKEDGITGFKEYFF** 169

**GmrSD|gi|322691502|B** **----RNIEELLLENRSKID-----------------------------MDRIEPGIQRGIRILSHELT------------------KDHFDRESFME-KL** 157

**GmrSD|gi|347536664|F** **---------------------------------------KYKIDGLSDPLYENVKMANGIKTITDFI----------------ENSERVERKKEFINYIY** 164

**GmrSD|gi|380567916|C** **--------------------KEFAKFCDEANKDNKSENNLFLQEIKKIPQDICQNIPRALETIANWF-----------------KDDTNDDIAIFSNFIY** 200

**GmrSD|gi|386866338|B** **PNQAEDVVVQDGPSDQENE----------------------------EMETMCRPLLDGYQIIRQYIA------------TSEKTSEKTSENRIDLEKLR** 190

**GmrSD|gi|389857445|S** **----------QQLFENSSA-----------------------------EFENMDEIIRGYQNTKLALN-------------KILSEQFKMEATVYAEYLF** 161

**GmrSD|gi|411028915|G** **---------------EAILQ---------------------APQNALATVPDTQCMQNVRQVMTGFR---------------EKRALDEAGIGKISRYIY** 158

**GmrS|gi|384207706|Gm** **-------------------------------------------------KITMQNIRNNYKYVKEYLDN--------------------NQNNKI--TLL** 122

310 320 330 340 350 360 370 380 390 400

....|....|....|....|....|....|....|....|....|....|....|....|....|....|....|....|....|....|....|....|

**Positive_selection/1** **----------------------------------------------------------------------------------------------------** 1

**Negative_selection/1** **--N------------N---NNN---N-NN-NN-NN------NNN-N--N-N------------N-N--N-------------------------------** 58

**GmrSD|gi|145633202|H** **DKVEIIRTEV-----PEGTDLNHYFEIMNIRGEQLEKHEVLKARLMKKLSGD-----IEKSLFAKIWDACS-------------DMSRYAVMGF---DSK** 228

**GmrSD|gi|147675278|V** **RQVQWVNNIV-----PTQMDLNRLFATMNTAGIQLEQADILKAKLFKHIHT-------DKAQYDAMWVACE-------------HLENYFERNV------** 258

**GmrSD|gi|319955102|C** **NQVQLVKVEI-----PSDIDVAHYFEVMNNRGEQLEEHEIVKARLLDKIKD----NKSGTAQFAKIWDACS-------------QMNKPIQRLFKK-DRA** 246

**GmrSD|gi|322691502|B** **AKIIVYRIEV-----PENTDLNRYYEIMNTRGEQLEQHDILKATLMSYLSD-----DSEKGLFAKIWDACS-------------DMTGYVQMHFIS-KNN** 233

**GmrSD|gi|347536664|F** **NKATFFISELP--KNYQMQDLNRYFEAMNATGRGLENHEILKVEILKKVPN------DKKVFYTKIWNAIS-------------EMDKCLIRQKTWSNEG** 243

**GmrSD|gi|380567916|C** **SNVKFVFVEL-----AQNTDLNRFFIRMNNRGKQLEKHEILKARLLKNISDK-----EERIVYAHIWDICS-------------QMDNYIFQKA------** 271

**GmrSD|gi|386866338|B** **TNVRVVRDEL-----PPGTDLNRYFEIMNTRGVQLRPQDIIKARLMSAVSS-----AADRETIGRIWDVCS-------------DMDHYMQCLATP-DER** 266

**GmrSD|gi|389857445|S** **QHVIIFRNML-----PTDLDLNLYFERFNSRGEQLEAHEIIKAQLMAKLDD------DEAAKFAKIWDACA-------------DFEKPVVKSFQMRKKR** 237

**GmrSD|gi|411028915|G** **ENVRFVRTEV-----PRRMDLNKLFEVINARGVQLQHHEILKARLLHAIPAE------ERQRYAALWDACA-------------DMKGFVDQTL------** 228

**GmrS|gi|384207706|Gm** **NNIIFTEIET-----DSEDEAFIFFDTQNGRGVTLDAIDYLKSYHLRA-----IKNDNYRDIIAEDWDKNNKYDKKKKHNNLNILFNEILWKARRWTGRN** 212

410 420 430 440 450 460 470 480 490 500

....|....|....|....|....|....|....|....|....|....|....|....|....|....|....|....|....|....|....|....|

**Positive_selection/1** **-----------------------------------------------------------------------------------P------------P---** 2

**Negative_selection/1** **---------------------N-------------------------------------------------------------------------N----** 60

**GmrSD|gi|145633202|H** **IRGVI-----------------FSDKWSEKPKC-FMEIIQ------------------------DIEEYNKEIEKKNKDENRKSPIINIQVGVDGI-KIL** 285

**GmrSD|gi|147675278|V** **----------RKVFPNADWYHIEPEHLASFDAE--RF--------------------------------------------AAKDETSEALSGLSIAELA** 302

**GmrSD|gi|319955102|C** **HF-------------------FGENFNSYHFKADEDI-----------------------------------------------NEDDYKDTFLSIHQIL** 280

**GmrSD|gi|322691502|B** **AVREAI---------------FGSEWNSMPPNS-WQKYKK------------------------------------------AISGTTQETTGHKICDLI** 275

**GmrSD|gi|347536664|F** **-------------------------------MDVFNQRRK--------------------------EALFINSIDDLCKKCNDLVSFEINDNIDSIKNIA** 286

**GmrSD|gi|380567916|C** **------------------------------SDRKISKLKESNEN--------------------------------------------------TIDTII** 291

**GmrSD|gi|386866338|B** **TRW------------------FGEDWSSVPPID-WNQLVG---------------------------------------AAGSGDGDGSVKSFKSFKDVL** 308

**GmrSD|gi|389857445|S** **KETFKERENI-----------FGWHFSNYKLANIYRFI------------------------------------------------DNTNSSKKSILDTL** 278

**GmrSD|gi|411028915|G** **-------------------------------RQ-------------------GRDKLSGSALATLYTQQKLACAPSVRDLLARQEALQETDDSLSGYRSL** 278

**GmrS|gi|384207706|Gm** **IHYE-------------------------NIKEIKREFEKNTKHNDNDQCLY------------------------------------------------** 239

510 520 530 540 550 560 570 580 590 600

....|....|....|....|....|....|....|....|....|....|....|....|....|....|....|....|....|....|....|....|

**Positive_selection/1** **------P---------------------------------------------------------------------------------------------** 3

**Negative_selection/1** **----------------------------------------------------------------------------------------------------** 60

**GmrSD|gi|145633202|H** **---------DLINGS-------------------------------------------------------------------------------------** 291

**GmrSD|gi|147675278|V** **SQVKASSIPDNT----------------------------------------------------------------------------------------** 314

**GmrSD|gi|319955102|C** **S----GASILDIEE--------------------------------------------------------------------------------------** 290

**GmrSD|gi|322691502|B** **E----KNFKVEDDEG-------------------------------------------------------------------------------------** 286

**GmrSD|gi|347536664|F** **PSN-------------------------------------------------------------------------------------------------** 289

**GmrSD|gi|380567916|C** **-----NRSFSKP----------------------------------------------------------------------------------------** 298

**GmrSD|gi|386866338|B** **REALNEEPLQAERQTQ------------------------------------------------------------------------------------** 324

**GmrSD|gi|389857445|S** **E----DSSSLVFDD--------------------------------------------------------------------------------------** 288

**GmrSD|gi|411028915|G** **AQIADADHEKTM----------------------------------------------------------------------------------------** 290

**GmrS|gi|384207706|Gm** **----------------YTNIENIMYIDKNITKDINSLVFNIKEKTMPNKNQMYINKLFETFNNRGRQLKQHEILKSYLLSKVNNRDIYSRIWDSCSIMDN** 323

610 620 630 640 650 660 670 680 690 700

....|....|....|....|....|....|....|....|....|....|....|....|....|....|....|....|....|....|....|....|

**Positive_selection/1** **----------------------------------------------------------------------------------------------------** 3

**Negative_selection/1** **----------------------------------------------------------------------------------------------------** 60

**GmrSD|gi|145633202|H** **---------------------------------------------------------------------------------KNSGVYKNDFVDKYDG---** 307

**GmrSD|gi|147675278|V** **----------------------------------------------------------------------------------------EQEKFETYDLDV** 326

**GmrSD|gi|319955102|C** **-----------------------------------------------------------------------------------------SKDVDKKE---** 298

**GmrSD|gi|322691502|B** **-----------------------------------------------------------------------------------------YVDGDVRV---** 294

**GmrSD|gi|347536664|F** **---------------------------------------------------------------------------------------------KRPDKKL** 296

**GmrSD|gi|380567916|C** **-----------------------------------------------------------------------------------------------NQDEG** 303

**GmrSD|gi|386866338|B** **---------------------------------------------------------------------------------------PGVRSDDEVE---** 334

**GmrSD|gi|389857445|S** **-----------------------------------------------------------------------------------------IVDDKETG---** 296

**GmrSD|gi|411028915|G** **---------------------------------------------------------------------------------------------QKEQVEG** 297

**GmrS|gi|384207706|Gm** **YIEKNLKDILKIDNETLRKLVNANDRGKADEKDVGVSENIEEYLKSIIKETENQDNLKSKRGLDFYDILNLKRNSDFEDLD----NQFENDLYNDSESNY** 419

710 720 730 740 750 760 770 780 790 800

....|....|....|....|....|....|....|....|....|....|....|....|....|....|....|....|....|....|....|....|

**Positive_selection/1** **----------------------------------------------------------------------------------------------------** 3

**Negative_selection/1** **---------NN-NN-NN-N--N----------------------------------------N-------------N--N---N---N---N---N----** 75

**GmrSD|gi|145633202|H** **-----SFTPVIDFPNFLMHVLRIYLEMTDKCGDFTKIAPLDEKYLLNSFEGQI------------------KDDEAVRNFIYVLLICRYLFDCYVI--KS** 382

**GmrSD|gi|147675278|V** **ET--VYCRPIIKFPLLLIHAYRVYLALNDHNDIEPRLHSDRLLEIFDPLIN--------------------GDEQSVKLFIETLWQVRYQFDRWVV-KWV** 403

**GmrSD|gi|319955102|C** **-----DFEAIIDFPNFLMHVLKLYFSEVKDIPLNGDELLKRFEGL--------------------------EDEIDALKFINKLLFYRVVFDRFIV--TV** 365

**GmrSD|gi|322691502|B** **-----RFESVIEFPYFLLHTLKVYVSLYGVTHEIATSKIVDELLDDKKLLDAFNRVVSCGITDTGRIAD--SKEDFARGFIICLLRTRYLFDKYII--KR** 385

**GmrSD|gi|347536664|F** **AT--RNERAILSFTEFLLQVLWLQITQEEKSNSIDFFNTNKLLQTFKLY----------------------LNENKASTFIDNLLRYRVIFDHYIL--RI** 370

**GmrSD|gi|380567916|C** **KQ--EKFKSIVDFPTFLLHVYKICNKQDITIDKNKLLEIIKI-----------------------------EDSGKAKAFIENLLKCRILFDYFIV-KGK** 371

**GmrSD|gi|386866338|B** **-----QYRSIISFPAFLMHVLRIYCGGESDASGDAKQISLDDAELLRVKVARIFSKAPENSEDSGDSDDSASAEDNAKRFAATLLKCRFLFDNCII--KT** 427

**GmrSD|gi|389857445|S** **-----RYTTVINFETLLLYTVSIIKNISPQDVQLDDKKLLDVFMVSE------------------------KGRDWVIYFSETLLSIRHLFDNYVI--RN** 365

**GmrSD|gi|411028915|G** **DD--RWADSIIGFPLFLLHVLRIWLYENSRKDLDRILDRDLTKIFEEFFFSSETPQ---------------QQEQDVKGFVDLVWTIRVLWDATII-KWT** 379

**GmrS|gi|384207706|Gm** **EA--PKIRSIISFPMLLLHTLRIFLIENKIAKTVDVKEKELINIFKHIFDDD-------------------NISKKTSYFFKLLWKIRVLFDKYVIKFVS** 498

810 820 830 840 850 860 870 880 890 900

....|....|....|....|....|....|....|....|....|....|....|....|....|....|....|....|....|....|....|....|

**Positive_selection/1** **-------------------------------P--------------------------------------------------------------------** 4

**Negative_selection/1** **-----------------------------N-------------------------------------N-------------N-N----N-----------** 80

**GmrSD|gi|145633202|H** **NTIRTGEEN------------------WSLWAVMPNDSSYYYKNTFGNNT-----------ESKNDEELDNSGDTKTKIVVMLLSMFHVSNPSRIYKNWL** 453

**GmrSD|gi|147675278|V** **ECDDATDAQLGLTYQSRSK-------------SNDTYYINRTQKELTDIVL-------------------------------LQSVRNFTGERSAQYWLT** 459

**GmrSD|gi|319955102|C** **IED-EKSE-------DNTK--------WVLQKPKMYYYEKKKTKKLQYENTFKD---------------------LQESIIKCLSMLQVTFRTKKYKNYL** 428

**GmrSD|gi|322691502|B** **EYA-ND-S-------ADGE--------WSLKSLYVSGQQSKKKAYYGNSEFTRS------------GEWTCTNDWRTKTNIMMQSALRVSYTSPKVMHWI** 456

**GmrSD|gi|347536664|F** **SHDEQS------ITN------------YFINYTEDETEENKK-------------------------------------LIQFQSMLYVSISYYLWLTPL** 415

**GmrSD|gi|380567916|C** **DDEENS---------------------YKIRRLDEEGKKFSKDFDDLAM---------------------------------VQNYLRVARSGMSNNHHH** 417

**GmrSD|gi|386866338|B** **RMD-AAAPS------DATE--------WTLKCCKENKKERSYYLANAFSNDC------------------------QQELIMIQSMFQVTETGNNHKNAL** 488

**GmrSD|gi|389857445|S** **ASEDTSRG-------SKND--------WFLSKGTYYEYQRDNRKNTDYFVEEHFENNTFPD------------KAVNDTIIMLQSMFAVTFTSNRDSRWL** 438

**GmrSD|gi|411028915|G** **EAEIRSESRPDRIHMLCKTSVSGER-------TTRNLTRSINYQENAELSL-------------------------------LQNMLYHTQENTTQYWMT** 441

**GmrS|gi|384207706|Gm** **DNEDGEVLSICNIIYR----------------KNSRSIERRIDKNNKALSM-------------------------------LQSILYYTQESITQYWLT** 551

910 920 930 940 950 960 970 980 990 1000

....|....|....|....|....|....|....|....|....|....|....|....|....|....|....|....|....|....|....|....|

**Positive_selection/1** **---------------------------------------------------------------------P-------P----------------------** 6

**Negative_selection/1** **-----------------------------------------------------------------N----------------------------------** 81

**GmrSD|gi|145633202|H** **YAVLR--------------------------------------------------------------WLFNN----KDNITPDNYI----HFLEEL----** 479

**GmrSD|gi|147675278|V** **PFLSGLIRSSIKQDSVALELLENIDNKMSLS---------------------------VDTQKEASFALAEG----------------------------** 504

**GmrSD|gi|319955102|C** **QEILS---------------------------------------------------------------WFGD-A-EDLEIES----------KEFLKKLN** 453

**GmrSD|gi|322691502|B** **TKLLI-------------------------------------------------------------WLSEADCK-NIKNDAITKFD----KVAENI----** 486

**GmrSD|gi|347536664|F** **LN-----------------------------------------------------------EIKENCNNSKDF---SLLEF-----------LKNW----** 438

**GmrSD|gi|380567916|C** **WLTPFLKIILKFINNDKLEISYERVNNEDKPEELQIKIGDYNITTNFQNFLNTLNPGEELIEFLENLDTALAKEQASAEEIKKDLLGISNDILDQLKI--** 515

**GmrSD|gi|386866338|B** **YSMLQ----------------------------------------------------------------WLW---GVEAVDGFAFL----QRIREY----** 513

**GmrSD|gi|389857445|S** **YETLV-------------------------------------------------------------YLFENVEH-LQDEAFGHQFV----IFLEAL----** 468

**GmrSD|gi|411028915|G** **PYLYYVYKNQNSPSDYYLKYLRHLDNYLLGE--------------------------ETDEPLVKRTRSFMK----------------------------** 487

**GmrS|gi|384207706|Gm** **PFLYQCIKINDANELYQYLMKLDNLLYFYK----------------------IKKSKTLSEKTIEISAIDID----------------------------** 601

1010 1020 1030 1040 1050 1060 1070 1080 1090 1100

....|....|....|....|....|....|....|....|....|....|....|....|....|....|....|....|....|....|....|....|

**Positive_selection/1** **------------------------------------------------------------------------------------------------------** 6

**Negative_selection/1** **-------------------------------------------------------------N----N--N--N---------------------------** 85

**GmrSD|gi|145633202|H** **-CDKFYFGNNCQGKDITE-----------------------IILDEVEFELNSGHKENWDRGVNVPNFVFNRLDYQLWRF--------------------** 535

**GmrSD|gi|147675278|V** **------------------------------------------IEPNCQSWQSQSTYFAKSLGTSFEHYWFQKLEYLIWKKMKASESSLPSEE-------L** 555

**GmrSD|gi|319955102|C** **TLVINTFNENRNYDE-------------------------------------VVKEPHYNKGTSTPHFLFNFIDYLYWTENQ------------------** 498

**GmrSD|gi|322691502|B** **-AIDAVRENFFQVCSN----------------------------------------GVYAMGVNTPHIVFNYLDYLLWNDDRNGKK--------------** 531

**GmrSD|gi|347536664|F** **-DNERLKNKSISLNYRL-----------------------------------------------IDRYWFWRLDYYFWENRKNWF---------------** 475

**GmrSD|gi|380567916|C** **-------------------------------------KNDQIDAENIFFIEDENGISVLDQGTATPHYWFYRLEYYLWKYSKIKYY--------------** 564

**GmrSD|gi|386866338|B** **-ARERLERALGLE----------------------------SLKDAPTEKRQSRIREAVSRGVGTEHFIFNYLDYALWVLLAPVDGSAQGED------VP** 578

**GmrSD|gi|389857445|S** **-AVRYADGRLFTEDGQ-----------------------------------------EFRCYPNMPIYAFNFIDYVLWKNQNNLQE--------------** 512

**GmrSD|gi|411028915|G** **------------------------------------------HPWRTGQLTNFADYLRGKHGVHFRHYWFYKLDFVLWHSAQRSDAQ-------------** 532

**GmrS|gi|384207706|Gm** **-------------------------------------GYYHDNTKTKNMIKKLKEELQNLNYYGCNRYWFYKLEFILWYNYYNNIDSNIFNQNDDNINKK** 664

1110 1120 1130 1140 1150 1160 1170 1180 1190 1200

....|....|....|....|....|....|....|....|....|....|....|....|....|....|....|....|....|....|....|....|

**Positive_selection/1** **----------------------------------------------------------------------------------------------------** 6

**Negative_selection/1** **----------------------------------------N-----NNNNN--NN-------------------------N-NNNN-N-----NN--NN-** 103

**GmrSD|gi|145633202|H** **---PQSVEILSKNDDWLTDNTKDAIW------------KKFRFTFRSSVEHHYPQHPIDGH---TIK----------E-IDMFGNLYLLSQSKNSRLSNL** 606

**GmrSD|gi|147675278|V** **NKFKK-----------------------------------YRITSKNSVEHVHPQNDEYNS---RL---------DCETLNSFGNLVLLSPGENSSYSNQ** 608

**GmrSD|gi|319955102|C** **SN------------------------------------YSFEFKYRNSVEHHLPQSYRN--------ES------NIDVLDCLGNLCLVSKSGNSKMNDE** 548

**GmrSD|gi|322691502|B** **KK-Y----------------------------------SDFVFEFRNSVEHWYPQNPSE-----GTFEQ-W----KDGV-DQFGNLCIIQRNVNSKFSNM** 585

**GmrSD|gi|347536664|F** **-KDNKPLLDIA---------------------------DKYLFKANRSIEHISPQTIMEGD---TRRI-------SDEKLDTFGNLVMISSGQNSSLQNE** 537

**GmrSD|gi|380567916|C** **LDGKNLKEIKLKDKNFGDIA------------------NNFYFRNLNSIEHVQAQSKANEK--------DW----DDENIDNFGNLALISSSFNSSLSNL** 634

**GmrSD|gi|386866338|B** **VV-SIFGADEPPTIASDVRTQWWEYTAQSDESMQALDCKQFRFRYRNSVEHFSPRHPLG-----GGA--------PDN-VDDFGNLCLLTVQENSRRNNL** 663

**GmrSD|gi|389857445|S** **YF-FDS--------------------------------RNFRFTYRRSVEHWYPQNPNFED--SGMLRM------SDSLLHSFGNLCIITDSQNSKFGNS** 571

**GmrSD|gi|411028915|G** **EEWKN-----------------------------------FRFTAKNSVEHISPQQPQPTD---YNIV-------SPDNRDDFGNLVFLSGGINSKYSNR** 587

**GmrS|gi|384207706|Gm** **IKEILNK---------------------------------FRITSKNSIEHVYPQNDTQLSND-----DKWSDETKEECLNSFGNLVLISGSLNSELNNK** 726

1210 1220 1230 1240 1250 1260 1270 1280 1290 1300

....|....|....|....|....|....|....|....|....|....|....|....|....|....|....|....|....|....|....|....|

**Positive_selection/1** **----------------------------------------------------------------------------------------------------** 6

**Negative_selection/1** **----N-----------------------N-------------N--------------------------N-N-------N--------------------** 109

**GmrSD|gi|145633202|H** **TPVAKRDI---I------KTWKNCDSLKQVI-----------M---------------MSHDKWGEDEIKVHGEKMLTILNRPLSKTDS-----------** 660

**GmrSD|gi|147675278|V** **DVDKKRID---F------ERKSHFDALKLREIFGVKGQGL-----------------------WGKQQIDNHLEDMMAVFAQHYKHQ-------------** 663

**GmrSD|gi|319955102|C** **SPKGKADETGKY--------YKESLPAKQLI-----------M-----------YKETNQKHKWERGEILKHYYDVLELLERRNEILSL-----------** 607

**GmrSD|gi|322691502|B** **SPEAKKST---F------KDMIVKGSIKLRI-----------M-----SELTEKNGDKPASLYWKERAYKAHEEEMLGHLKKACNIE-------------** 647

**GmrSD|gi|347536664|F** **SFEVKKAH---I-KEFITGRNGTIESLKMLH-----------I---------------YNYDTWNDENLNNHNDVMVSILINSFSDNYIDIKKQLNNFIT** 607

**GmrSD|gi|380567916|C** **DTQDKYL----Y-----INKDNNIISLKLWL-----------I----------YALSEKNQLNWTFNKAQEHRRQMLEILKKSFNNGHDKA---------** 695

**GmrSD|gi|386866338|B** **DAMEKIRH---F--------NVGQQSLKFQF-----------M-----------VGQAQKENAWGKPQIEKQTELWVQLLD-ALSMASRSR---------** 720

**GmrSD|gi|389857445|S** **RPQAKYSQ---W------EKIFGNQSLKLQW-----------M----------AKLTGNSDDNWNSEVIRGHEHKILTLVKEFFESTKNI----------** 631

**GmrSD|gi|411028915|G** **PFNEKKIK---F---ENIKKHGRIESLKMDL--------------------------IYQNAHWNDEVMERHRESMIKSLENYYREDI------------** 643

**GmrS|gi|384207706|Gm** **SYKFSILR--------DKLKKGEVQSLKSLL-----------ILKNNK---------------WDENACKEHLNKVIELYKEYYEKYTK-----------** 781

...

**Positive_selection/1** **---** 6

**Negative_selection/1** **---** 109

**GmrSD|gi|145633202|H** **---** 660

**GmrSD|gi|147675278|V** **---** 663

**GmrSD|gi|319955102|C** **---** 607

**GmrSD|gi|322691502|B** **---** 647

**GmrSD|gi|347536664|F** **NKS** 610

**GmrSD|gi|380567916|C** **---** 695

**GmrSD|gi|386866338|B** **---** 720

**GmrSD|gi|389857445|S** **---** 631

**GmrSD|gi|411028915|G** **---** 643

**GmrS|gi|384207706|Gm** **---** 781

**References**

1. Kosakovsky Pond SL, Frost SD: **Not so different after all: a comparison of methods for detecting amino acid sites under selection**. *Molecular biology and evolution* 2005, **22**(5):1208-1222.

2. Murrell B, Wertheim JO, Moola S, Weighill T, Scheffler K, Kosakovsky Pond SL: **Detecting individual sites subject to episodic diversifying selection**. *PLoS genetics* 2012, **8**(7):e1002764.

3. Delport W, Poon AF, Frost SD, Kosakovsky Pond SL: **Datamonkey 2010: a suite of phylogenetic analysis tools for evolutionary biology**. *Bioinformatics* 2010, **26**(19):2455-2457.
