# Supplementary material for: Phylogenomics and sequence-structure-function relationships in the GmrSD family of Type IV restriction enzymes
Source: BMC Bioinformatics. 2015 Oct 23;16:336. doi: 10.1186/s12859-015-0773-z (PMC4619093; doi:10.1186/s12859-015-0773-z)
Supplement: Additional file 7: — Results of protein-DNA docking and electrostatic potential analysis for GmrS and GmrD domains. (PDF 671 kb) [file 12859_2015_773_MOESM7_ESM.pdf]

A.

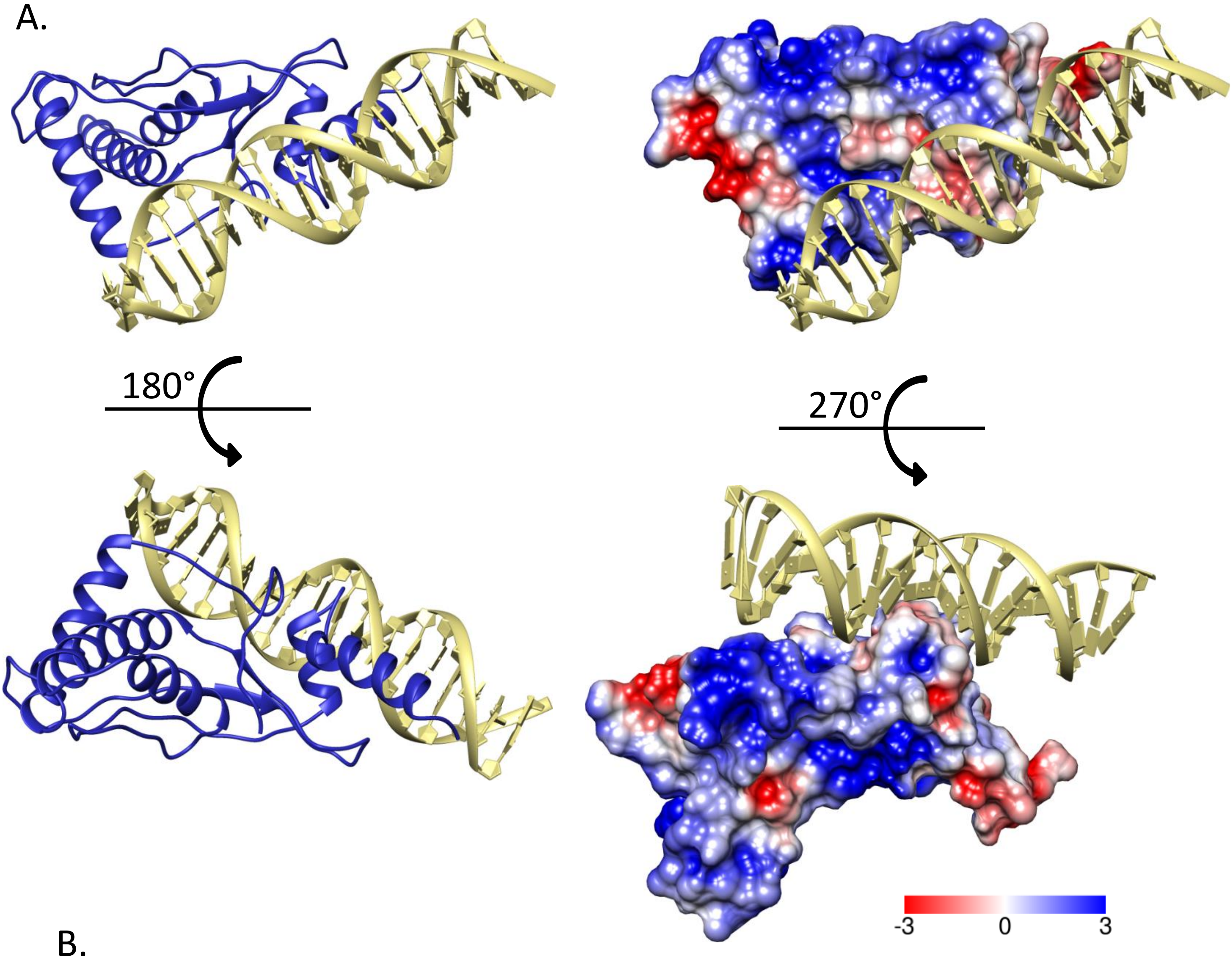

B.

>gi|21327769|gb|AAM11681.2|AF493796_1 GmrS [Escherichia coli]

MKSETLTIQQIFQNQRQYRVPFY**QRAY**VWT**Q**RN**Q**WSALLEDIFEKAQSRLSGTKPTPHFLGAVVLEPQLK
NSLLGVDTIHI**I**DGQQRLTTLQYILASLASIRLSLRATGLSELEGLVLTCLKNTNEATMRNKKVECFKLW
PTFRD**Q**T**H**F**IQSLNV**D**N**IDDLRNVFSDSFTQHGTLRKHFNHPPSLEALWFFTEAFIKWIKIENHSPQENA
VALIEAVLTDLKLVSIFLEAEDDAQIIFETLNGRGAELHATDLIRNYIFMCAEHENINAIELYENEWKIF
EDKYWSEKQRRGRINKPRMEWLVHATLQSERQQ

Supplementary Figure 1. **A.** The model of the GmrS domain in complex with DNA in the ribbon representation (left) and in the surface representation (right). The surface is colored according to the distribution of electrostatic potential, from red (negatively charged) to blue (positively charged. The structure of the complex was predicted using the NPdock server [1] with restraints on DNA-binding amino acid residues derived from sequence conservation and results from the RBscore server [2]. The distribution of electrostatic potential was calculated using the PDB2PQR server [3] and the APBS software package [4]. **B.** GmrS domain sequence with DNA-binding residues in bold and underlined. The DNA-binding residues were identified in the model using UCSF Chimera [5] and default contact criteria.

A.

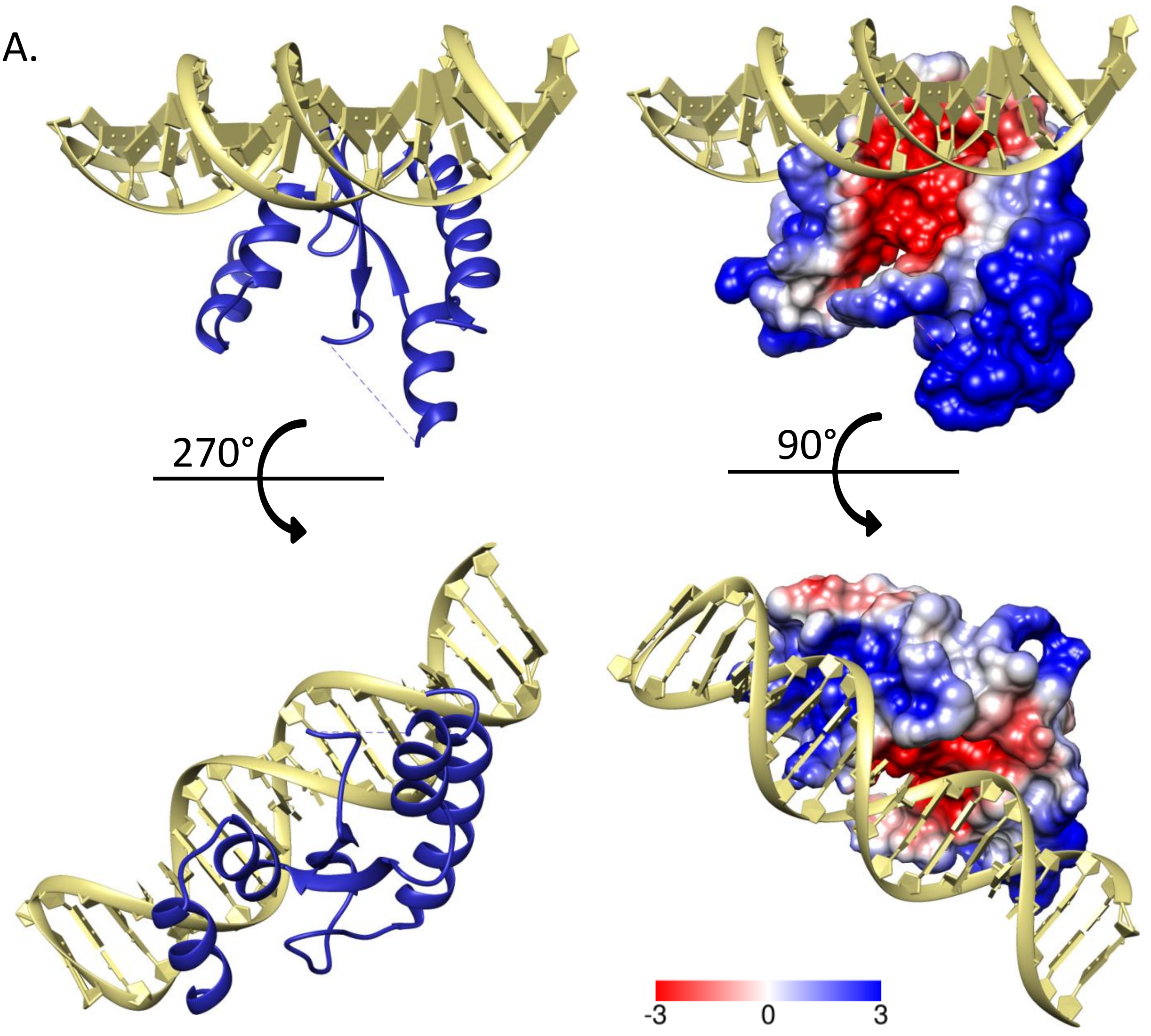

B.

>gi|21327771|gb|AAM11682.2|AF493797_1 GmrD [Escherichia coli]

MYNDLVSYVVRRSVCGLTPKNYNNVFMNVLRHLSKTEISSVELRNILNSLNGEASRWPGDSEFLNACINA

PLYPGRLDAPKM**R**SM**L**TELERELCRQVKTEKPDVPNL**SNLDI**DHLMPQSWYSCWPLENGHMVTNSDATVM

NQIVLSGTDLTPEQLLVRKRQQAIATLGNLTLL**NLS**VN**R**SV**Q**NAVFLKKRDALIV**H**TNLRLNIPLILKDK

WDESEIQERGKKLGEIALKVWPKYD

Supplementary Figure 2. **A.** The model of the GmrD domain in complex with DNA in the ribbon representation (left) and in the surface representation (right). The surface is colored according to the distribution of electrostatic potential, from red (negatively charged) to blue (positively charged. The structure of the complex was predicted using the NPdock server [1]. The distribution of electrostatic potential was calculated using the PDB2PQR server [3] and the APBS software package [4]. **B.** GmrD domain sequence with DNA-binding residues in bold and underlined. The DNA-binding residues were identified in the model using UCSF Chimera [5] and default contact criteria.

References:

1. Tuszynska I, Magnus M, Jonak K, Dawson W, Bujnicki JM: **NPDock: a web server for protein-nucleic acid docking**. *Nucleic acids research* 2015, **43**(W1):W425-430.
2. Miao Z, Westhof E: **Prediction of nucleic acid binding probability in proteins: a neighboring residue network based score**. *Nucleic acids research* 2015, **43**(11):5340-5351.
3. Dolinsky TJ, Nielsen JE, McCammon JA, Baker NA: **PDB2PQR: an automated pipeline for the setup of Poisson-Boltzmann electrostatics calculations**. *Nucleic acids research* 2004, **32**(Web Server issue):W665-667.
4. Baker NA, Sept D, Joseph S, Holst MJ, McCammon JA: **Electrostatics of nanosystems: application to microtubules and the ribosome**. *Proceedings of the National Academy of Sciences of the United States of America* 2001, **98**(18):10037-10041.
5. Pettersen EF, Goddard TD, Huang CC, Couch GS, Greenblatt DM, Meng EC, Ferrin TE: **UCSF Chimera--a visualization system for exploratory research and analysis**. *Journal of computational chemistry* 2004, **25**(13):1605-1612.
