# Supplementary material for: Phylogenomics and sequence-structure-function relationships in the GmrSD family of Type IV restriction enzymes
Source: BMC Bioinformatics. 2015 Oct 23;16:336. doi: 10.1186/s12859-015-0773-z (PMC4619093; doi:10.1186/s12859-015-0773-z)
Supplement: Additional file 9: — The phylogenetic tree of double-domain GmrSD sequences. Description of data: Phylogenetic tree calculated for sequences of double-domain GmrSD proteins and sigle-domain GmrS-GmrD pairs treated as one sequence. Clades with SH-like support values below 80 % were collapsed. The tree was calculated using approximated maximum-likelihood approach implemented in FastTree [67, 68]. (PDF 2577 kb) [file 12859_2015_773_MOESM9_ESM.pdf]

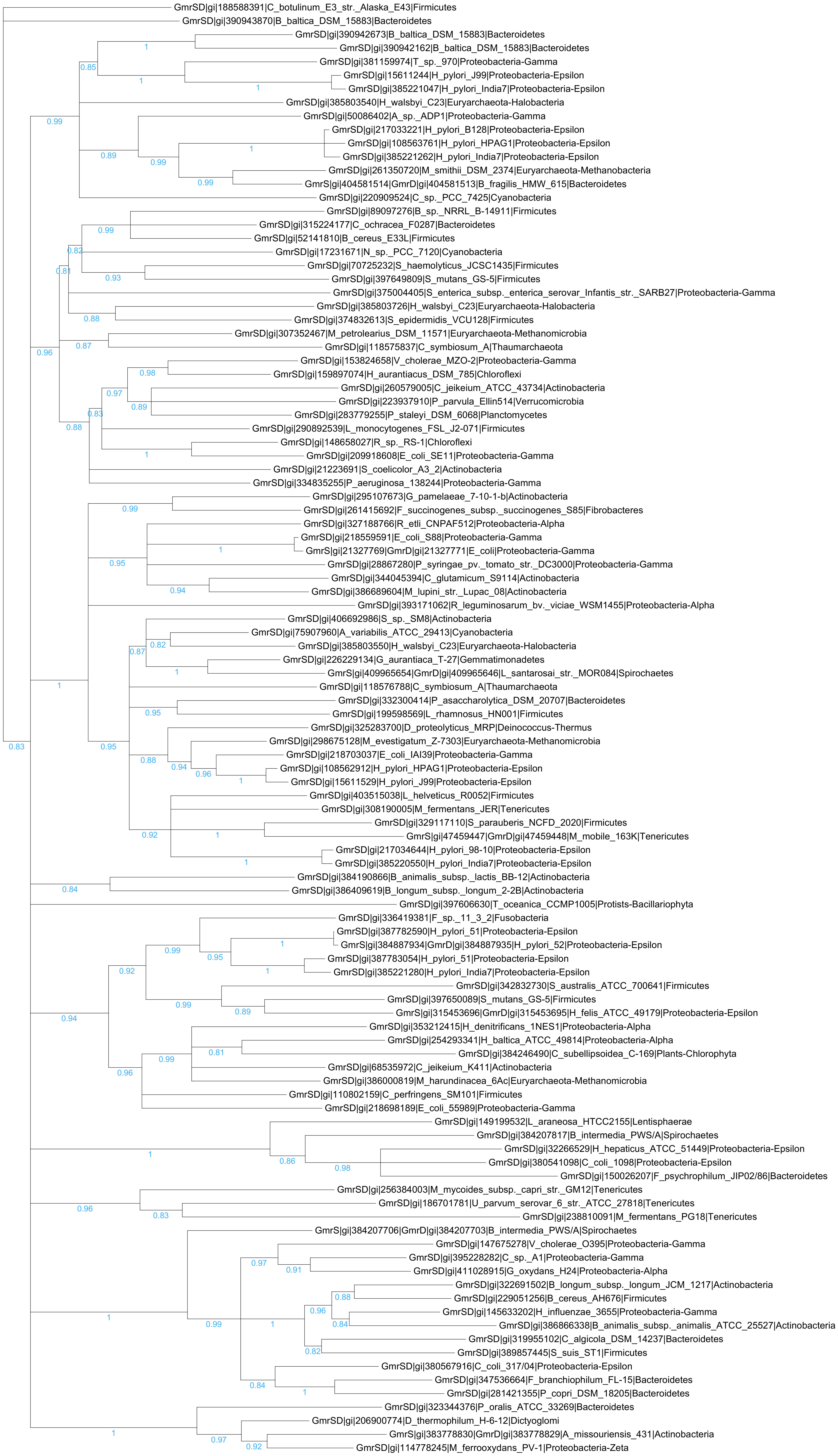

Supplementary Figure 3 The phylogenetic tree of double-domain GmrSD sequences

Phylogenetic tree calculated for sequences of double-domain GmrSD proteins and sigle-domain GmrS-GmrD pairs treated as one sequence. Clades with SH-like support values below 80% were collapsed. The tree was calculated using approximated maximum-likelihood approach implemented in FastTree .
